# General Anesthesia Disrupts Complex Cortical Dynamics in Response to Intracranial Electrical Stimulation in Rats

**DOI:** 10.1523/ENEURO.0343-20.2021

**Published:** 2021-08-04

**Authors:** A. Arena, R. Comolatti, S. Thon, A. G. Casali, J. F. Storm

**Affiliations:** 1 Department of Molecular Medicine, University of Oslo, Oslo 0372, Norway; 2 Institute of Science and Technology, Federal University of São Paulo, São José dos Campos, 12247-014, Brazil

**Keywords:** anesthesia, connectivity, consciousness, OFF period, perturbational complexity index, rat

## Abstract

The capacity of human brain to sustain complex cortical dynamics appears to be strongly associated with conscious experience and consistently drops when consciousness fades. For example, several recent studies in humans found a remarkable reduction of the spatiotemporal complexity of cortical responses to local stimulation during dreamless sleep, general anesthesia, and coma. However, this perturbational complexity has never been directly estimated in non-human animals *in vivo* previously, and the mechanisms that prevent neocortical neurons to engage in complex interactions are still unclear. Here, we quantify the complexity of electroencephalographic (EEG) responses to intracranial electrical stimulation in rats, comparing wakefulness to propofol, sevoflurane, and ketamine anesthesia. The evoked activity changed from highly complex in wakefulness to far simpler with propofol and sevoflurane. The reduced complexity was associated with a suppression of high frequencies that preceded a reduced phase-locking, and disruption of functional connectivity and pattern diversity. We then showed how these parameters dissociate with ketamine and depend on intensity and site of stimulation. Our results support the idea that brief periods of activity-dependent neuronal silence can interrupt complex interactions in neocortical circuits, and open the way for further mechanistic investigations of the neuronal basis for consciousness and loss of consciousness across species.

## Visual Abstract

**Figure:**
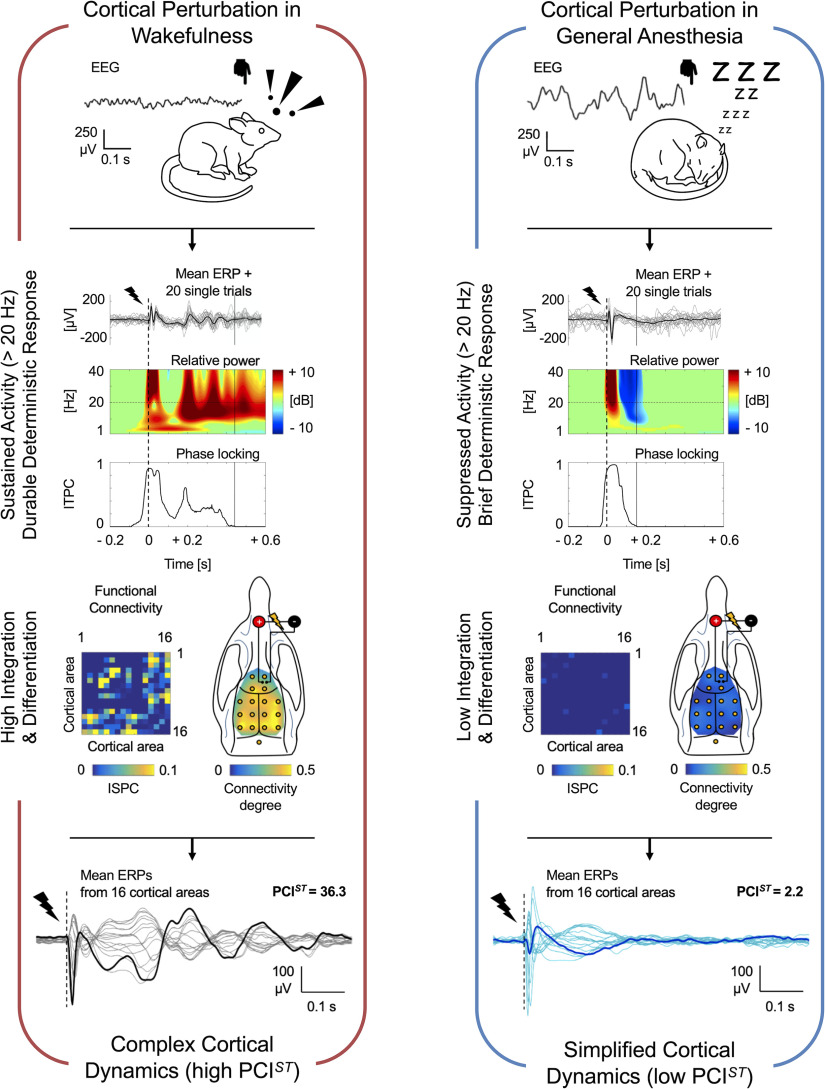

## Significance Statement

The perturbational complexity index (PCI) is an electrophysiological metric for the capacity of cortical circuits to integrate information. PCI proved to be a reliable, objective, report-independent index that discriminates between conscious and unconscious states in humans, with promising clinical implications in brain disorders. However, the neural mechanisms underlying PCI remain uncertain and difficult to test, because the method has never been directly applied to non-human species *in vivo* before. Here, we reproduce PCI in rats, thus setting the stage for invasive, mechanistic investigations. We show how PCI correlates with functional connectivity and pattern diversity, and collapses from wakefulness to general anesthesia. Finally, we provide evidence for the role of transient, sleep-like putative periods of neuronal silence in preventing complex cortical interactions.

## Introduction

A longstanding challenge in neuroscience has been the identification of robust and neuron-based measures of consciousness. Recently, brain complexity, defined as the combination of functional differentiation and integration in thalamocortical systems, has gained growing attention as promising candidate (Casali et al., 2013; Dehaene, 2014; Koch et al., 2016). For example, a reliable association between conscious states and complexity of global network dynamics has been demonstrated by means of both electroencephalography (EEG; Schartner et al., 2015) and imaging of spontaneous brain activity (Demertzi et al., 2019). A highly accurate method to assess complexity of causal, cortical dynamics is the perturbational complexity index (PCI), which was originally introduced and validated for discrimination between unconscious and conscious unresponsive patients (Casali et al., 2013; Casarotto et al., 2016). PCI is based on a perturbational approach: brief transcranial magnetic stimulation (TMS) or intracranial electrical stimulation are used to trigger cortical activity, and the spatiotemporal complexity of the EEG-recorded event-related potentials (ERPs) is quantified. Long-lasting responses that are both temporally differentiated and distributed among cortical areas (high PCI) are evoked whenever subjects have conscious experiences, such as during wakefulness, rapid eye movement (REM) sleep or ketamine anesthesia, when dreams or hallucinations occur (Casali et al., 2013; Sarasso et al., 2015; Casarotto et al., 2016; Rosanova et al., 2018; Comolatti et al., 2019). In contrast, during dreamless non-REM (NREM) sleep, general anesthesia, and unresponsive wakefulness syndrome (UWS; or “vegetative state”), the ERPs appear to be either local and short-lasting (less integrated), or global and stereotyped (less differentiated), yielding low PCI (Casali et al., 2013; Sarasso et al., 2015; Casarotto et al., 2016; Rosanova et al., 2018; Comolatti et al., 2019).

Although the PCI method has been thoroughly tested in humans, it has previously never been directly transferred to any non-human species *in vivo.* Therefore, it is not clear to what extent rodent brains can sustain the complex dynamics in response to brief direct stimulation that are characteristic of conscious brain states in humans. Developing animal models and extending the application of PCI is of paramount importance, since the neuronal mechanisms underlying the engagement or interruption of complex cortical activations are still uncertain, and mechanistic investigations may lead to intervention strategies for restoring complexity and awareness in brain-injured, unconscious patients. Here, we implement and test the PCI method in rats. By measuring the complexity of intracranial EEG responses to cortical stimulation, we show that the awake rat brain supports complex cortical activations, which turn into simplified, less integrated responses during general anesthesia. We further demonstrate that the disruption of long-lasting complex activations is associated with suppression of high-frequency (HF) EEG power and reduced phase-locking, supporting the hypothesis that neuronal hyperpolarization might prevent cortical neurons from engaging in durable, complex interactions (Pigorini et al., 2015; Tononi et al., 2016; Sanchez-Vives et al., 2017; Storm et al., 2017). The main results of this study have previously been presented in abstract form at conferences and as a preprint (Arena et al., 2018, 2020).

## Materials and Methods

### Animal model

Experimental and animal care procedures were conducted at the University of Oslo and were approved by the Norwegian Authority Mattilsynet (FOTS ID: 11812) in agreement with Norwegian law of animal handling. Experiments were conducted on adult male Sprague Dawley rats (∼300–500 g, *n* = 12). All efforts have been made to avoid/minimize animals’ distress and pain during the entire course of experimentation. Rats were caged in enriched environments, with *ad libitum* access to food and water and were exposed to 12/12 h light/dark cycle at 23°C constant room temperature. The experiments were conducted during the afternoon within the light phase of the cycle.

### Electrodes and surgical procedure

All coordinates for electrodes implantation are expressed referring to bregma position, x = medial-lateral axis (–, left hemisphere; +, right hemisphere), y = rostral-caudal axis (–, caudal to bregma; +, rostral to bregma), z = dorsal-ventral axis. ERPs were triggered by electrical stimulations delivered by a bipolar electrode chronically implanted in right secondary motor cortex (M2), composed by two insulated tungsten wires (50-μm caliber; ∼500-μm distance between wires), and recorded by 16 stainless steel screws (1.2-mm caliber) electrodes. The bipolar electrode was inserted perpendicularly to the cortical surface, along the coronal plane and the coordinates (in mm) for implantation were: x = +1.2 left wire/+1.7 right wire; y = +3.7; z = +1.9. Recording electrodes were in contact with the dura and were organized in a grid, symmetric along the sagittal suture, and were placed at the following coordinates (in mm): x = ±1.5, y = +5 (M2); x = ±1.5, y = +2 (M2); x = ±1.5, y = –1 (primary motor cortex; M1); x = ±4.5, y = –1 (primary somatosensory cortex; S1); x = ±1.5, y = –4 (retrosplenial cortex; RS); x = ±4.5, y = –4 (parietal associative cortex; PA); x = ±1.5, y = –7 (secondary visual cortex; V2); x = ±4.5, y = –7 (primary visual cortex; V1); x = 0, y = –10 (cerebellum, ground; GND). Impedances of all electrodes were measured *in situ* at 1 kHz at the beginning of each recording session, for each rat. The averaged impedance across all sessions, channels and animals was 0.17 ± 0.03 MΩ for stimulating electrodes and 7.12 ± 0.42 kΩ for recording electrodes (mean ± SEM).

Surgical implantation of electrodes was performed under controlled, deep anesthesia with sevoflurane 2.5–5% (Baxter) in oxygen concentrated (O_2_ >85%), humidified room air (constant rate: 0.5 l/min). Body temperature was maintained at 36.5–37.5°C by a heating pad, and subcutaneous injections of butorphanol (2 mg/kg) and dexamethasone (0.2 mg/kg) were given. Absence of response to painful stimulation was ensured and standard sterile procedures were used throughout the surgical operation. Holes were drilled through the exposed skull at the desired coordinates, with stereotaxic guidance. Electrodes were positioned and two machine screws were also upside-down mounted over the caudal part of the skull for subsequent head restriction. Dental acrylic was applied over the entire exposed skull, sealing the wound margins and securing electrodes in place. After surgery, buprenorphine (0.1 mg/kg) and meloxicam (1 mg/kg) were subcutaneously injected. For the following 3- to 4-d rats were checked for possible signs of distress, infection or damages to electrode implantation. Buprenorphine (0.1–0.05 mg/kg) and dexamethasone (0.2–0.1 mg/kg) were also subcutaneously injected once a day.

### Experimental procedures

After recovery, rats were gradually habituated in three consecutive days to body and head restriction in a custom-made recording setup, consisting of a horizontally oriented, elevated transparent acrylic cylinder, in which the rat was introduced (with only the head and the tail outside). Right above the tube, two clamps fixed a head-bar that was connected to the machine screws that were chronically implanted on the skull of the rat, over the cerebellum. The head-bar was adjusted and clumped to sustain and fix the head of the rat in a natural and stable position so that no objects interfered with the natural whisking behavior during wakefulness. A heating blanket also wrapped the bottom half of the acrylic tube to keep the body temperature of the rat at 36.5–37.5°C during general anesthesia. After one week from surgery, rats underwent several electrophysiological stimulation/recording sessions during wakefulness, followed by general anesthesia. Recording sessions were interleaved with a resting period of at least 48 h. Three different general anesthetics were tested (sevoflurane, propofol and ketamine) in randomized different days and most of the rats were exposed to all anesthetics. At the end of the last recording session, during general anesthesia, an electrolytic lesion was performed by applying 30 μA for 30 s to the poles of the stimulating electrode. Rats were then killed with a lethal dose of pentobarbital (140 mg/kg, i.v.) and, after the suppression of corneal reflex, intracardially perfused with PBS (phosphate buffer solution with heparin 5000 IU/l) and 4% paraformaldehyde in PBS at 4°C for tissue fixation. Brains were then extracted and processed for histologic Nissl staining.

During the recording session the tail vein was cannulated (26-G catheter) for allowing intravenous infusion of propofol or ketamine, when needed and the stimulating electrode was connected to an isolated current stimulator (Isolator HG203, High Medical) triggered by a voltage pulse generator (2100, AM Systems). Recording electrodes were connected to a 16-channel unipolar amplifier board with common reference shorted to ground (RHD2132, Intan Technologies), controlled by Open Ephys system (Siegle et al., 2017), and the epidural EEG signal was acquired and digitized at 10 or 30 kHz, 16-bit resolution. Stimulation/recording was conducted in darkness after ∼45 min of acclimatization in the setup. Depending on the randomized recording session, rats were exposed to several electrical monophasic current pulses (duration 1 ms) of 50 μA or of various intensities (40, 60, 80, and 100 μA, organized in randomized blocks) delivered at 0.1 Hz during wakefulness. Stimulations were repeated during subsequent general anesthesia within the same animal. General anesthesia was induced either by exposure to sevoflurane 5%, or by the intravenous bolus injection of propofol 10 mg/kg (B-Braun) or ketamine 30 mg/kg (Vetoquinol), then maintained at the initial constant dosage of either sevoflurane 2.5% (by a gas mask, in humidified air, O_2_ > 85%, at 0.5 l/min), propofol 1 mg/kg/min or ketamine 1.75 mg/kg/min (by a syringe pump). Subcutaneous injection of glycopyrrolate 0.01 mg/kg was also performed during ketamine anesthesia to reduce the induced increase in salivation. Eye ointment was applied to keep eyes moist and body temperature was kept at 36.5–37.5°C by a heating blanket system. The initial dosages were chosen in accordance with the literature (Idvall et al., 1980; Brammer et al., 1993; Benito et al., 2010; Arena et al., 2017) and adjusted in a set of pilot experiments on five rats, in which the loss of righting reflex was evaluated by gently laying down the rats on their backs, on a flat surface, without any physical constraint. Any muscular movements toward acquiring a normal, upright position were considered as a sign of reflex maintenance. The final initial dosages were the minimal for maintaining loss of righting reflex in 100% of the tested animals in the pilot experiments. At these dosages, reactions to pain stimulations were still present. Thus, for the experiments presented here, all rats were checked for reaction to pain stimulations (pinching the tail by a forceps) after 10 min from induction, and the concentration (for sevoflurane) or infusion rates (for propofol and ketamine) were increased in a stepwise manner, in steps of 4% of the initial dosage with 3 min between each increment, until the pain response was absent. The resulting averaged experimental dosages across rats and sessions were: sevoflurane 2.58 ± 0.03%, propofol 1.06 ± 0.02 mg/kg/min and ketamine 1.83 ± 0.03 mg/kg/min (mean ± SEM). We did not detect any spontaneous body movements during general anesthesia, by visual inspection through the wall of the transparent acrylic tube used to maintain the rat in position.

A subset of animals was exposed to a final stimulation/recording session with the purpose to detect putative movements of whiskers induced by electrical stimulation of M2. Whiskers of the left mystacial pad were clipped, except for the most caudal vibrissa of the third row (C1), which was tracked by a high-speed camera (500 frame/s, Blackfly S. Mono 0.4 MP USB3, FLIR System). Experimental area was constantly illuminated only by dim red light (wavelength 655 nm, LED, Quadica), which is not detectable by rat retina (Jacobs et al., 2001; De Farias Rocha et al., 2016). A second LED 655 nm was triggered by the stimulation system, signaling the onset of current pulses to the camera. During wakefulness, head and body restrained rats were exposed to several monophasic current pulses of 1 ms at either 50 or 100 μA, or to train stimulations as positive control (Brecht et al., 2004; train duration 0.3 s, composed by 11 single monophasic current pulses of 1 ms and 50 μA, rate of pulse 33 Hz). Different stimulations were grouped in randomized blocks and delivered at a rate of 0.2 Hz. In order to avoid saturation artefacts in the amplification system during train stimulation, the epidural EEG activity was recorded by a bipolar amplifier board (RHD2216, Intan Technologies) controlled by Open Ephys system (Siegle et al., 2017), and the frontal-occipital (M2-V2) derivation, ipsilateral to the stimulation, was adopted for only this session. At the end of stimulation/recording, rats were deeply anaesthetized (sevoflurane 5%) and killed with a lethal dose of pentobarbital (140 mg/kg, i.v).

### Analysis of electrophysiological signal

Analysis of electrophysiological data were performed in MATLAB2016a (The MathWorks) and Origin 9.1 (OriginLab). Raw epidural EEG recordings were visually inspected to remove channels containing noise artefacts or having impedance >1 MΩ. EEG signals from all electrodes were re-referenced to the common average across channels for analyzing ERPs, while a bipolar frontal-occipital derivation (M2-V2 right) was chosen for analyzing spontaneous activity. Stimulus artefacts were removed and signal was spline interpolated in a time window from 0 to 0.005 s from stimulus onset. EEG signal was band pass filtered from 0.5 to 80 Hz (Butterworth filter, 3rd order) and down-sampled to 500 Hz. ERP epochs from −5 to 5 s centered at the stimulus onset (0 s) were then extracted for each channel. All epochs were offset corrected by subtracting the average voltage of their respective baseline (from −1 to 0 s). Trials with high voltage artefacts in their baseline were removed. Threshold for rejection was set at the averaged root mean square (rms) of baseline (from −1 to 0 s) across trials +3 SDs. The first *n* = 90 consecutive trials of preprocessed signal were used for analysis of evoked responses to electrical perturbations of 50 μA and spontaneous EEG activity was quantified from baseline epochs of 4 s (from −5 to −1 s) from the corresponding trials (with the exception of one animal in one wakefulness condition, for which 80 trials were used in analysis instead). Whereas *n* = 56 consecutive ERPs were used for analysis of evoked responses to current simulations of increasing intensities.

In order to quantify the different states of brain activity induced by general anesthetics in relation to wakefulness, fast Fourier transform (FFT) was performed on *n* = 90 epochs of spontaneous EEG activity and normalized by the number of samples N. The squared modules of the normalized FFTs were computed and the resulting power spectrums were averaged across trials. The 1/f relation of the averaged periodogram was then linearly fitted in Log-Log coordinates, in the frequency range from 20 to 40 Hz. The slope of the obtained linear function was considered to be the spectral exponent of the 1/f function and was used to quantify the (re)distribution of frequency powers in the spontaneous EEG activity (Colombo et al., 2019).

The amplitude of the evoked response was quantified by the rms amplitude of the first deflection of the mean ERP (0.006–0.05 s from stimulus onset) from each electrode and then averaged across channels.

The slow component (SC) of the ERP was quantified from low-pass filtered responses (<4 Hz, Butterworth filter, 3rd order). To test any dependency on the spontaneous slow activity, we computed the rms amplitude of the spontaneous SC in a long baseline period (−2–0 s) for each single trial and then averaged across trials and channels, and compared with the rms amplitude of the evoked SC (0–0.3 s), also computed for each single trial, then averaged across trials and channels. The maximal amplitude and latency of the evoked SC were obtained for each channel, from the maximal absolute peak of the ensemble average across trials (SC max), within the time range 0–0.6 s.

Morlet wavelet convolution was performed on each trial for all channels to extract both spectral powers and phases of ERPs. A total of 40 wavelets (three cycles) linearly spanning from 1 to 40 Hz were adopted. Powers from each channel, trial and frequency were normalized over the averaged power across trials in baseline window (−0.5 to −0.2 s) for each respective frequency and channel. Relative powers were then averaged across trials for each channel and frequency, and converted in dB. To identify only the significant positive and negative variations of dB with respect to baseline, bootstrap statistic was performed (500 permutations; positive and negative thresholds, α = 0.05) and not significant values were set to 0. The spectral content of the ERP was quantified by averaging the relative powers in frequency bands: δ (1–4 Hz), θ (5–7 Hz), α (8–14 Hz), β (15–25 Hz), and γ (26–40 Hz), then across time (from 0 to 0.5 s) and across channels. The putative “OFF periods” were detected as power suppression (<0 dB) in the HF range (20–40 Hz) after stimulation (Pigorini et al., 2015; Rosanova et al., 2018). Starting and end time points of periods of HF suppression were identified in a time window from 0 to 0.3 s as first downward and last upward zero crossing of the resulting dB signal. Minimum dB peak in the same time window was detected, and the HF power was quantified by averaging the relative power in the frequency range of 20–40 Hz, from 0.08 to 0.18 s. The starting point of the time window used to quantify HF power was empirically determined and was the mean starting point of HF suppression across anesthetic conditions. Potential later increments of HF power (>0 dB) were detected in a time window from 0.08 to 0.8 s. We also quantified increments of low-frequency (LF) activity by averaging the relative power in range 1–4 Hz and by detecting the maximal positive dB peak in time range 0–0.6 s.

The deterministic effect that the electrical stimulation had on the EEG response was measured as the duration of increased phase-locking of subsequent ERPs compared with baseline (Pigorini et al., 2015; Rosanova et al., 2018). Phase-locking at each channel, frequency and time point was computed across trials as intertrial phase clustering (ITPC; Cohen, 2014), with the following formula:

$$ITPC_{tf}=\left|n^{-1}\overset{n}{\underset{r=1}{\sum }}e^{ik_{tfr}}\right|.$$

Where *n* is the number of trials and *e^ik^* is the complex polar representation of the phase *k* from the trial *r* at time-frequency point *tf*. ITPC can assume values from 0 (no phase-locking) to 1 (maximal phase-locking), and bootstrap statistic (500 permutations; threshold, α = 0.01) was performed for each frequency and channel, to conserve only the statistically significant increments of ITPC with respect to baseline (from −0.5 to −0.2 s). Significant ITPC values were averaged across a broad band frequency range (8–40 Hz; Pigorini et al., 2015; Rosanova et al., 2018), and the “ITPC drop” was defined as the time point of the last significant mean ITPC value in time window 0–0.8 s. The latency of the maximal ITPC value in LF range (1–4 Hz, LF ITPC max) was also computed for each channel and then averaged.

A similar phase-based approach was adopted to quantify increments in functional connectivity across channels following the stimulation compared with baseline. For each trial, phase differences across channels at each frequency and time point were computed and the clustering over trials of resulting phase differences was defined as intersite phase clustering (ISPC; Cohen, 2014) and calculated with the following formula:

$$ISPC_{tf}=\left|n^{-1}\overset{n}{\underset{r=1}{\sum }}e^{i{\left(k_{x}-k_{y}\right)}_{tfr}}\right|.$$

Where *n* is the number of trials and $e^{i(k_{x}-k_{y})}$ is the complex polar representation of the difference between the phases *k_x_*and *k_y_* from the channels *x* and *y*, for the trial *r* at time-frequency point *tf.* Therefore, ISPC represents the consistency of the phase configuration between the activity from two channels across trials, at each time-frequency point. ISPC of each channel pair and frequency is then baseline corrected by subtracting the corresponding average value in the time window from −0.5 to −0.2 s and bootstrap statistic (500 permutations; positive and negative thresholds, α = 0.05) was performed to maintain only the statistically significant ISPC variations from baseline. All ISPC scores that could be explained by volume conduction (clustering of phase difference around 0 or π) were excluded from the analysis and the resulting ISPC values from each channel pair were averaged in the frequency range 5–14 Hz in two time windows, during the period of HF suppression (0.08–0.18 s) and immediately later (0.18–0.3 s). The ISPC scores > 0 represented significant functional connections between channel pairs and the connectivity degree (CD) was defined as the number of significant functional connections for each electrode, normalized over the number of channels minus one (to exclude autocorrelation). Rats with more than two removed channels (broken or noisy electrodes) were excluded from the connectivity analysis.

The spatiotemporal complexity of EEG responses to electrical stimulations was calculated using the recently introduced PCI state-transition (PCI*^ST^*; Comolatti et al., 2019), which quantifies the number of state transitions (NST) present in the principal components of the signal’s response. Briefly, the principal components accounting for 99% of the variance present in the response are obtained through singular value decomposition and then selected based on a minimum signal-to-noise ratio (SNR_min_). Then, for each component the number of significant state transition (ΔNST), a metric derived from recurrent quantification analysis (Marwan et al., 2007), is computed. PCI*^ST^* is the product between the number of principal components surviving dimensionality reduction and the average NST across components (Comolatti et al., 2019). Hence, PCI*^ST^* is high when a response displays multiple, linearly independent components (spatial differentiation), each contributing with significant amounts of state transitions (temporal complexity). In order to minimize the amount of baseline-like oscillations (noise) that contributed to the PCI*^ST^* value, SNR_min_ was chosen using a bootstrap procedure in the following way: for each signal, PCI*^ST^* was calculated on 16 surrogates for which complexity should be zero, generated using two random non-response 0.5 s segments (*t* < 0 s or *t* > 1.5 s, where 0 s is the stimulation onset) as baseline and response. SNR_min_ was then set to 1.8 for all analyses, the smallest value for which the median PCI*^ST^* across all surrogates was zero. The baseline and response window were defined as from −0.5 to −0.005 s and from 0 to 0.6 s form the stimulus onset, respectively, in accordance with previous experiments with intracranial stimulation in humans (Comolatti et al., 2019). To assess how the complexity of the EEG responses varied in time, PCI*^ST^* was calculated in shorter 0.1 s sliding windows from stimulus onset (0.02-s shift, until 1.1 s) and in the time range 0.08–0.6 s from the stimulus onset. PCI*^ST^* was computed using the available code at https://github.com/renzocom/PCIst and further parameters were set as previously reported (Comolatti et al., 2019).

### Analysis of whisker tracking

The video recording of the whisker movements in response to electrical stimulation was initially analyzed in Bonsai software (Lopes et al., 2015). The centroid of the whisker was tracked offline, and the relative space coordinates in the Cartesian plane were extracted for each frame and imported in MATLAB. Whisker positions were converted into degrees in the polar plane, obtaining angular oscillations in time that were analyzed similarly to the voltage signal. From the continuous signal, *n* = 21 consecutive motor responses centered on the stimulus onset (0 s) were extracted (from −2.5 to 2.5 s). Each motor trial was offset corrected by subtracting the respective mean angle of the baseline (time window from −1 to 0 s) and all the analyses were performed at the level of single trials for each rat. The magnitude of the whisker oscillation in response to the stimulus was quantified by the rms amplitude in a time window of 0.25 s following the stimulus offset and compared with the rms of the baseline (from −0.5 to −0.25 s). The mean spectral power of the whisker oscillation was also computed. A three-cycle Morlet wavelet convolution was performed with a family of 100 wavelets spanning linearly from 1 to 100 Hz. The powers of each frequency from all trials were extracted and normalized over the corresponding mean power across trials in the baseline (from −0.8 to −0.3 s). The relative powers of each frequency were then averaged over trials and converted into dB. Bootstrap statistic (500 trial-based permutations, thresholds α = 0.05) was performed and the non-significant angle variations with respect to the baseline were set to 0. The resulting relative powers were then averaged in a broad band frequency range (from 5 to 100 Hz), in the first 0.25 s after the stimulus offset.

### Histologic staining

After fixation, brains were exposed to increasing concentrations of sucrose (10%, 20%, 30%) in PBS solutions at 4°C for 4 d. Brains were quickly frozen in sucrose 30% in PBS and sliced in coronal sections of 50-μm thickness with a sliding microtome. Coronal sections were then prepared for Nissl staining. Sections were first dehydrated in increasing concentration of ethanol (70%, 95%, 100%) and immersed in xylene (VWR). Slices were rehydrated with decreasing concentration of ethanol (100%, 95%, 70%, 50%) and stained with Cresyl echt violet solution (incubation at 60°C for 13 min, Abcam). Sections were then rinsed in H_2_O_dd_, dehydrated in ethanol and then mounted and secured with coverslip on microscope slides. Brain sections were scanned at 5× with an AxioScanZ1 slide scanning microscope (Carl Zeiss) and estimation of electrode positions was conducted using ZEN imaging light software (Carl Zeiss).

### Statistics

All results are expressed as mean ± SEM, error bars represent SEM and nonparametric statistics were adopted. In a repeated measure design with dependent variables having more than two levels, principal effect of the variable was tested with Friedmans test. Group comparisons in repeated measures design were tested with Wilcoxon signed-rank (S-R) test, otherwise Mann–Whitney test was adopted. Estimation statistics were also performed with the web application https://www.estimationstats.com for group comparisons, and the effect size is reported in the figures as bootstrap resampling distribution of mean difference (bias-corrected and accelerated, 5000 resamples), with 95% confidence interval (CI) represented by a bold black line below the distribution. In the text, the effect size is reported as mean difference [95% CI: lower bound, upper bound of the interval] (Ho et al., 2019). Linear fitting was performed with the least-square method and error bars were used as weights when averages across rats were fitted. To evaluate correlations and goodness of fit, the coefficient of determination *R*^2^ was computed and *t* test was performed to test the null hypothesis of slope = 0, establishing a *p* value. Gaussian v test was used to test volume conduction in connectivity analysis (Cohen, 2014). All statistics are two-tailed. The statistical significance in figures is represented as follows: *p <* *0.05, **p <* *0.01, ***p <* *0.001, *p *≥* *0.05 ns (not significant).

### Data and materials availability

The code for computation of PCI*^ST^* is available in GitHub at the following link: github.com/renzocom/PCIst. Electrophysiological data used for analysis are available in EBRAINS at the following DOIs: https://doi.org/10.25493/S0DM-BK5 and https://doi.org/10.25493/5ZJY-PHB.

## Results

### Single pulse electrical stimulation triggered complex ERPs during wakefulness but not during propofol anesthesia

**Figure 1.**
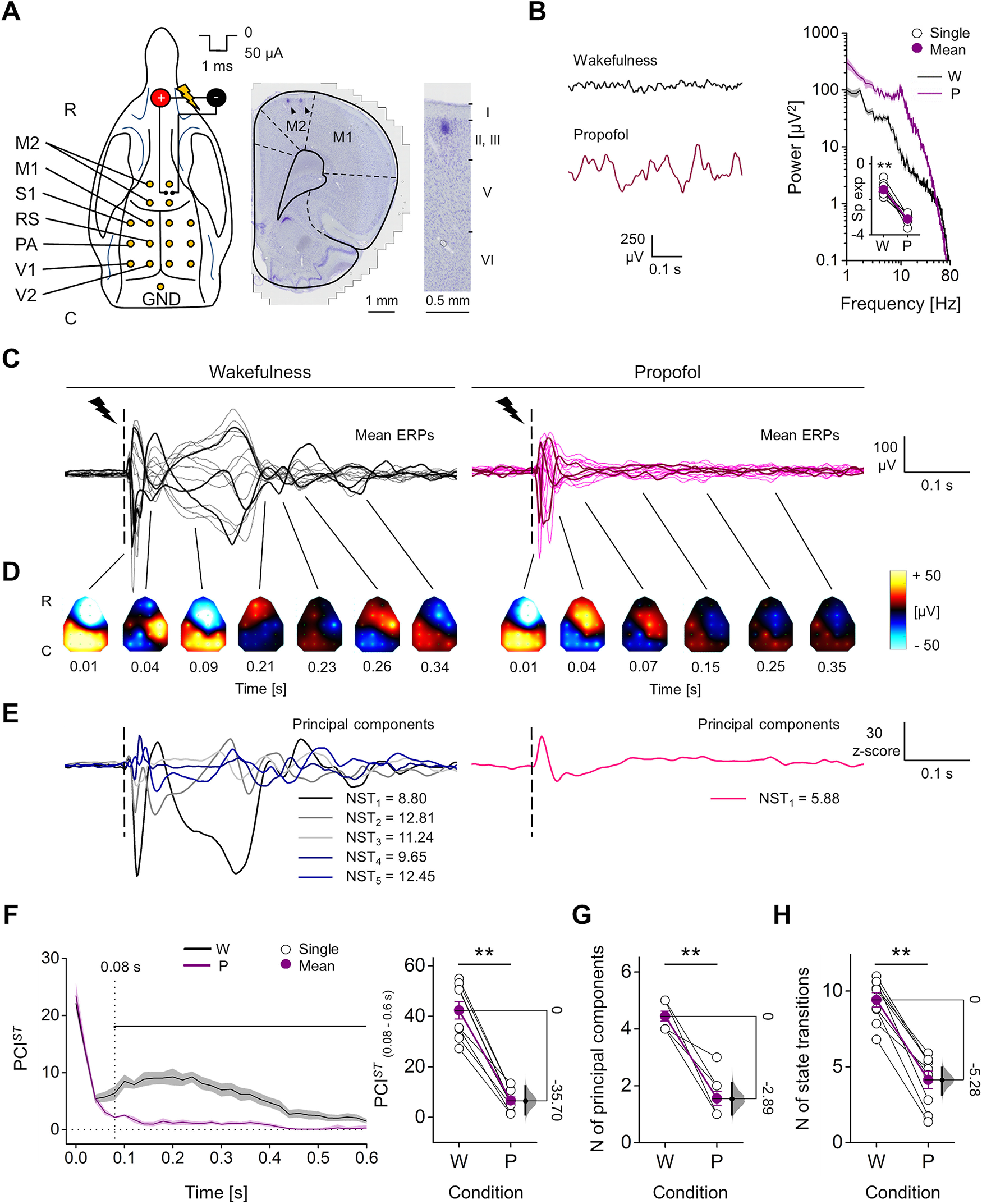
Spatiotemporal dynamics of evoked responses to electrical stimulation of M2 during wakefulness and propofol anesthesia. ***A***, left, Positions in the rat skull of the 16 screw electrodes (yellow dots) and bipolar stimulating electrode used for recording EEG and triggering ERPs (R-C: rostral-caudal). Right, Coronal brain section (Nissl staining) showing the location of stimulating electrode in right M2. Black arrowheads indicate marks from the two poles of the bipolar electrode. Far right, Magnified view showing the site of one pole relative to cortical layers. ***B***, left, Spontaneous EEG from one rat during wakefulness (W) and propofol (P) anesthesia. Right, Mean periodograms of spontaneous activity from one animal in the same conditions (shades represent SEM), and spectral exponents from all rats (inset). ***C–E***, EEG responses to single pulse electrical stimulation (1 ms, 50 μA; dashed line) from one rat during wakefulness and propofol anesthesia. Butterfly plots show superimposed mean ERPs from all recording electrodes (ERPs from three channels are in bold for clarity; ***C***) and their spatial distributions at different time points (interpolated ERPs, color-coded; ***D***). ***E***, Derived principal components (from the same data as in ***C***, ***D***) with corresponding NST. ***F***, left, Time course of PCI*^ST^* averaged from nine rats in wakefulness and propofol anesthesia (0 s: stimulus onset; shades represent SEM; horizontal line indicates statistical difference, *p *<* *0.05). Right, PCI*^ST^* quantified within the time window 0.08–0.60 s. Number of principal components (***G***) and average NST (***H***) across conditions for all rats. The floating axis on the right of each simple group comparison (panels ***F***, right, ***G***, ***H***) shows the mean difference between conditions. The effect size is reported as bootstrap resampling distribution of mean difference, with 95% CI represented by the bold black vertical line. See Extended Data Figure 1-1 for examination of possible whisker deflections induced by electrical stimulation. See Extended Data Figure 1-2 for assessment of the reproducibility of ERPs in time. See Extended Data Figure 1-3 for examination of possible correlation of ERP amplitude and length with stimulus location. See Extended Data Figure 1-4 for explanation of PCI*^ST^* decomposition in number of principal components and state transitions.

We recorded epidural EEG activity from 16 screw electrodes chronically implanted through the skull in head-restrained and body-restrained male, adult rats. Recording electrodes were in contact with the dura and organized in a symmetric grid, covering most of the cortex in both hemispheres (M2; M1; S1; RS; PA; V1; V2; GND). We stimulated right M2 by single monophasic, electrical current pulses (typically: 1-ms duration, 50-μA amplitude, at 0.1 Hz) via a chronically implanted bipolar electrode, located 4.38 ± 0.26 mm rostral from bregma, 0.47 ± 0.09 mm below the cortical surface (based on histology after recording, eight rats; Fig. 1*A*). The tips of the bipolar electrode were mainly located within Layer II/III across rats. However, one rat had one of the tips of the bipolar electrode placed in Layer I (0.05 mm from cortical surface), while another rat had one tip of the bipolar electrode placed in the upper part of Layer V (0.92 mm from cortical surface). Pulse trains delivered at similar rostral-caudal coordinates triggered coordinated whisker deflections (Brecht et al., 2004), whereas EEG responses following single stimuli were not measurably contaminated by movements (Extended Data Fig. 1-1) and were reproducible throughout recording sessions and across days (Extended Data Fig. 1-2). No correlation between the stimulating electrode locations and ERP amplitude or duration was found (Extended Data Fig. 1-3).

10.1523/ENEURO.0343-20.2021.f1-1 Extended Data Figure 1-1 Single pulse electrical stimulation of M2 did not trigger detectable whisker movements. ***A***, up, Example of five consecutive ERPs (grey traces) with ensemble average (black) during wakefulness, in response to two different electrical stimulations of right M2 (left, train of pulses of 1 ms, 50 μA, rate 33 Hz, train duration 0.3 s; right, single pulse, 1 ms, 50 μA; 21 stimulations delivered at 0.2 Hz) with relative spectrogram below. Middle, Tracking of the angular movement of left C1 whisker from the same rat, in response to stimulations (five consecutive single motor responses in grey and ensemble average in black) with relative spectrogram. Bottom, View from above of the rat’s snout and C1 whisker (R-C: rostral-caudal) at three time points. White arrowheads indicate whisker positions, while the white dot in the bottom-left side represents the onset of the electrical pulse. Only the train stimulation is able to trigger a detectable motor response of the left C1 vibrissae, spanning in a broad frequency range. ***B***, The rms amplitude of the angular motor response to the train stimulation and to the single pulse stimulation at 50 and 100 μA is quantified at the level of each animal (*n* = 4 rats). The rms amplitudes of 0.25 s after the stimulus offset (post) were compared with those obtained from 0.25 s before the onset of stimulation (pre; baseline, from –0.5 to –0.25 s) for all the 21 evoked responses (Wilcoxon S-R test; Train stimulation, for all rats: *p *=* *5.957 × 10^−5^; single pulse 50 μA, for rat 1: *p *=* *0.715, for rat 2: *p *=* *0.114, for rat 3: *p *=* *0.170, for rat 4: *p *=* *0.068; single pulse 100 μA, for rat 1: *p *=* *0.931, for rat 2 and rat 3: *p *=* *0.664, for rat 4: *p *=* *0.339). Averaged rms values across rats have been also calculated. Train stimulation: prestimulus 0.94 ± 0.17°, poststimulus 8.25 ± 1.43°; single pulse 50 μA: prestimulus 0.46 ± 0.16°, poststimulus 0.62 ± 0.26°; single pulse 100 μA, prestimulus 0.50 ± 0.12°, poststimulus 0.56 ± 0.16°). ***C***, In order to be able to detect also putative small and not phase-locked oscillations of left C1 vibrissae induced by electrical stimulation, a wavelet convolution on whisker tracking was performed. Only the increments or decrements in power for each frequency from 5 to 100 Hz that were statistically different from baseline (*p *<* *0.05) have been considered and averaged in a time window of 0.25 s after the offset of stimulation across rats (*n* = 4). The train of pulses induced a clear increase of power in the broad frequency range (12.49 ± 0.38 dB), but the single pulse stimulations did not trigger any clear power increase as it was close to baseline values (0 ± 0 and 0.07 ± 0.07 dB in response to single pulses of 50 and 100 μA, respectively). Download Figure 1-1, TIF file.

**Figure 2.**
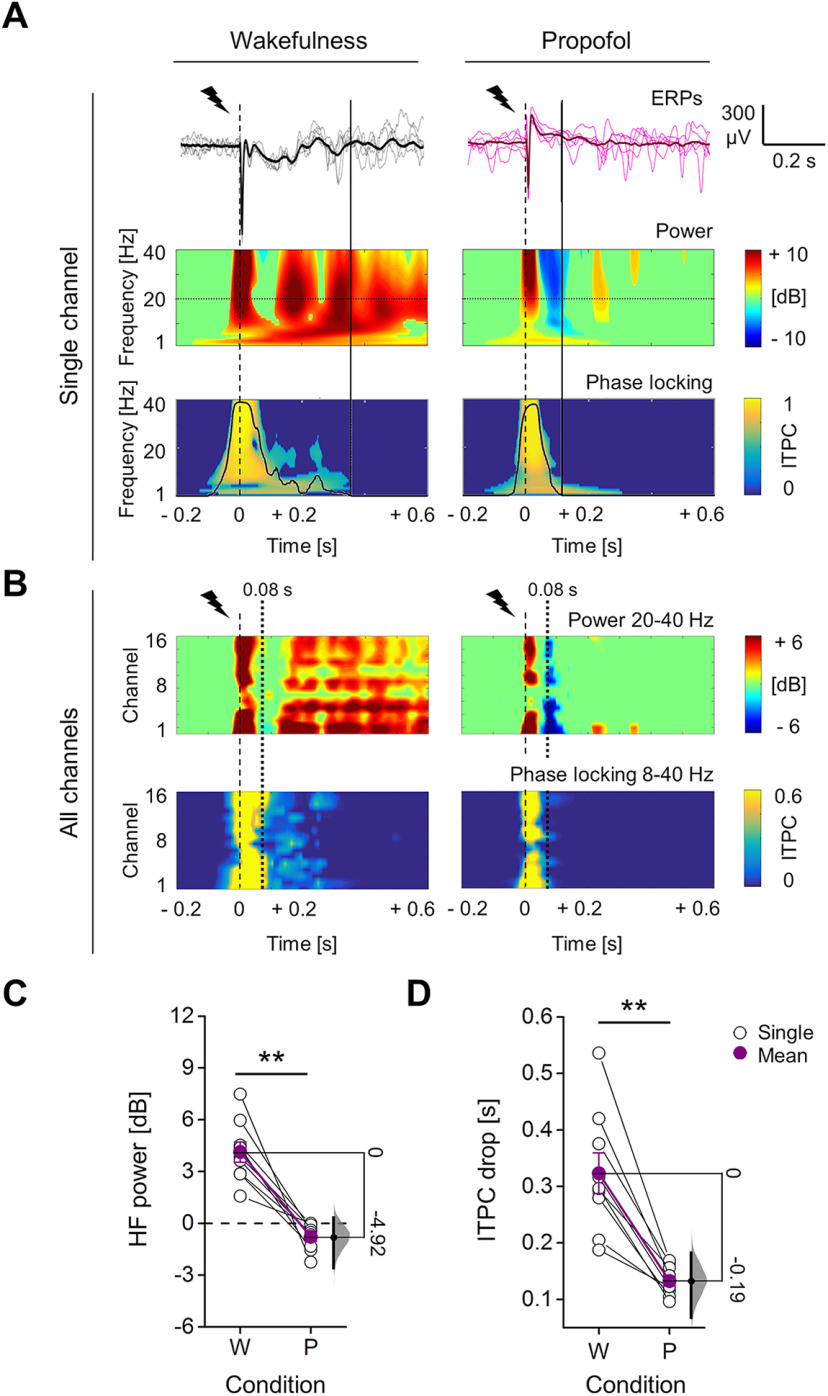
Propofol anesthesia induced suppression of high frequencies and reduced phase-locking in response to electrical stimulation, compared with wakefulness. ***A***, Example of epidural EEG response to single pulse electrical stimulation (1 ms, 50 μA; dashed line) from the same rat during wakefulness (left) and propofol anesthesia (right). The mean ERPs (bold) and five consecutive single trials from the same frontal channel (M2) are shown in both conditions (top) with relative spectrogram (middle) and ITPC (below) for all frequencies in range 1–40 Hz. The temporal dynamic of averaged ITPC in range 8–40 Hz is represented by a black superimposed curve that goes from 0 to 1 (ordinate axis on the right, same values of color map). The continuous vertical lines indicate the time point of the drop of averaged ITPC in range 8–40 Hz. ***B***, Time course of the average HF power in the 20- to 40-Hz range (top), and the averaged ITPC in the 8- to 40-Hz range (below) plotted for all channels from the same rat and conditions of ***A***. The dotted vertical line at 0.08 s indicates the mean onset of HF suppression across rats during propofol anesthesia. The mean HF power (in time range: 0.08–0.18 s; ***C***) and the duration (***D***) of phase-locking across trials (time of ITPC drop) for all animals (*n* = 9) during wakefulness (W) and propofol anesthesia (P). The floating axis on the right of each simple group comparison (panels ***C***, ***D***) shows the mean difference between conditions. The effect size is reported as bootstrap resampling distribution of mean difference, with 95% CI represented by the bold black vertical line. See Extended Data Figures 2-1, 2-2 for examination of single trial responses and SC of ERPs in propofol and wakefulness conditions, respectively. See Extended Data Figure 2-3 for in depth examination of HF suppression, comparing propofol anesthesia to wakefulness conditions.

10.1523/ENEURO.0343-20.2021.f1-2 Extended Data Figure 1-2 Single pulse electrical stimulation of M2 triggered reproducible ERPs that were reliable across different recording sessions. ***B***, Example of five consecutive ERPs (grey traces) with ensemble average (black) during wakefulness, in response to single pulse electrical stimulations of M2 (dashed line, 50 μA, 1 ms) from the same rat and channel, in different recording sessions, performed in different days. Below, The relative spectrograms are shown, with the ITPC averaged across frequencies (range 8–40 Hz). ITPC quantify the degree of reproducibility of the ERPs (phase-locking across trials). The drop time of ITPC is highlighted by a black line and indicates the duration of the phase-locked response induced by the stimulus (ITPC drop). ***B–D***, Quantification of the reliability of ERPs in response to single pulse stimulations from *n* = 8 rats, across 3 recording sessions (s1, s2, s3) performed in different days (∼4 d between each session). ***B***, the relative mean spectral power of the ERPs (up to 0.5 s) differed across frequency bands (Friedman test, *p *=* *1.189 × 10^−5^), with a peak in alpha (8–14 Hz) and beta (15–25 Hz) ranges, but no difference across sessions was identified (Friedman test, *p *=* *0.088). ***C***, The cortical excitation in response to stimulation was measured as the rms amplitude of the first deflection of the mean ERPs (Early ERP rms, up to 0.05 s from stimulus onset) and no variation across different days was detected (Friedman test, *p *=* *0.687). ***D***, Likewise, no significant variation across sessions was detected in the time of ITPC drop (Friedman test, *p *=* *0.072). Download Figure 1-2, TIF file.

**Figure 3.**
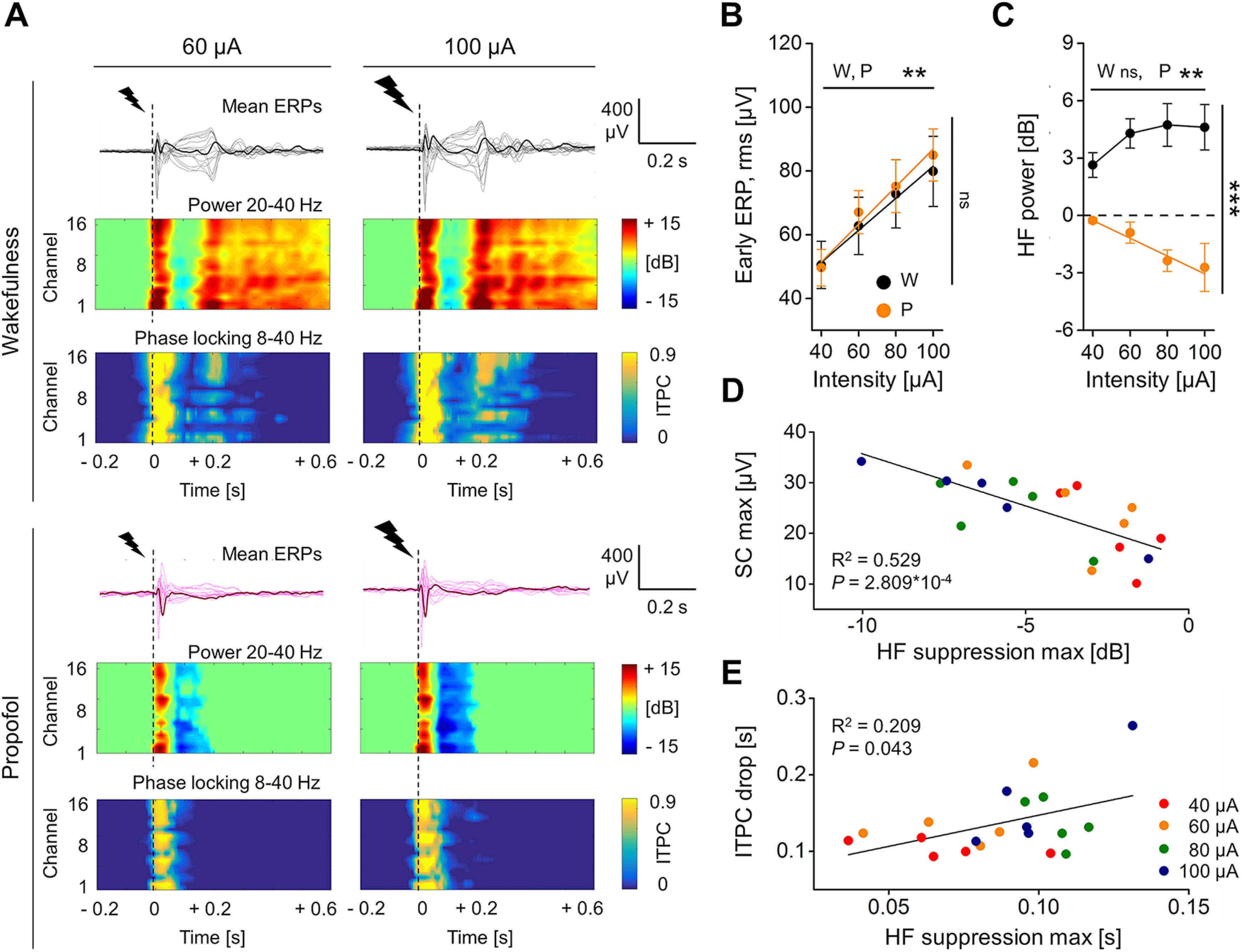
During propofol anesthesia, the HF suppression was deeper after stronger stimulation, and correlated with the SC of the ERP and with the drop in phase-locking. ***A***, Mean ERPs from the same rat in response to single pulse electrical stimulations (dashed lines) at two different intensities [60 μA (left); 100 μA (right)], during wakefulness (top) and during propofol anesthesia (bottom). The butterfly plots show averaged ERPs from all recording electrodes superimposed, with one mean ERP from the same parietal channel (PA) shown in bold for clarity. Below, The HF power (20–40 Hz) and the ITPC (8–40 Hz) for all channels are shown. ***B***, Quantification of early ERP rms amplitude (up to 0.05 s; ***B***) and HF power (in range 0.08–0.18 s; ***C***) as a function of increasing stimulation intensity during both wakefulness (W) and propofol anesthesia (P). Values are averaged across channels and animals (*n* = 5). ***D***, During propofol anesthesia, the maximal value of HF suppression (negative peak of HF power in range 0–0.3 s) correlated in magnitude with the maximal absolute amplitude of the SC of the ERP (in range 0–0.6 s). ***E***, The maximal HF suppression also correlated in time with the drop of phase-locking across trials (ITPC drop). The averaged values across channels are plotted for each rat and stimulus intensity (color-coded). The coefficient of determination R^2^ and the *p* value are reported. See Extended Data Figure 3-1 for examination of correlations between ITPC drop time and different time points of HF suppression. See Extended Data Figure 3-2 for analysis of PCI*^ST^* and ITPC drop time in relation to increasing stimulus intensity.

10.1523/ENEURO.0343-20.2021.f1-3 Extended Data Figure 1-3 Amplitude of ERPs and duration of phase-locked response were not related with the stimulus location in M2. ***A***, Representation of coronal sections of rat brain with actual position of chronically implanted bipolar electrodes in right M2 from eight rats (dots represent the two poles of the bipolar electrode and each rat is color coded in each slice). Coordinates have been measured from Nissl-stained coronal sections of rat brains. Among the tested animals, stimulating electrodes covered a cortical area from ∼5 to ∼3 mm with respect to bregma in rostral-caudal direction (R-C, *y*-axes, mean position 4.38 ± 0.25 mm) and a depth range from ∼0.1 to ∼0.8 mm calculated from cortical surface (Z axes, mean position 0.47 ± 0.09 mm, averaged values for each electrode between the two poles), mainly corresponding to Layer II/III. On average the 2 electrode poles were separated by 0.46 ± 0.03 mm in the medial-lateral direction (*x*-axes). ***B***, ***C***, mean rms amplitude of the first deflections of the ERPs (up to 0.05 s from stimulus onset, *up*) and mean duration of phase-locking among subsequent ERPs (ITPC drop, 8–40 Hz, bottom) are plotted for all rats (*n* = 7) and recording sessions (1 to 3 recordings for each rat) during wakefulness against the position of the stimulating electrode along the *y*-axes (***B***) and along the *z*-axes (***C***). Possible correlations with the position of stimulating electrode have been tested and no significant relation was detected between the Early ERP rms amplitude and the position of electrodes along the *y*-axes (***B***, up; linear fit, *p *=* *0.121, *R*^2^ = 0.144) or along the Z axes (***C***, up; linear fit, *p *=* *0.122, *R*^2^ = 0,143). No significant relation was identified between the ITPC drop and the position of electrodes along the *y*-axes (***B***, bottom; linear fit, *p *=* *0.35, *R*^2^ = 0.055) or along the Z axes (***C***, middle; linear fit, *p *=* *0.122, *R*^2^ = 0.143). Download Figure 1-3, TIF file.

**Figure 4.**
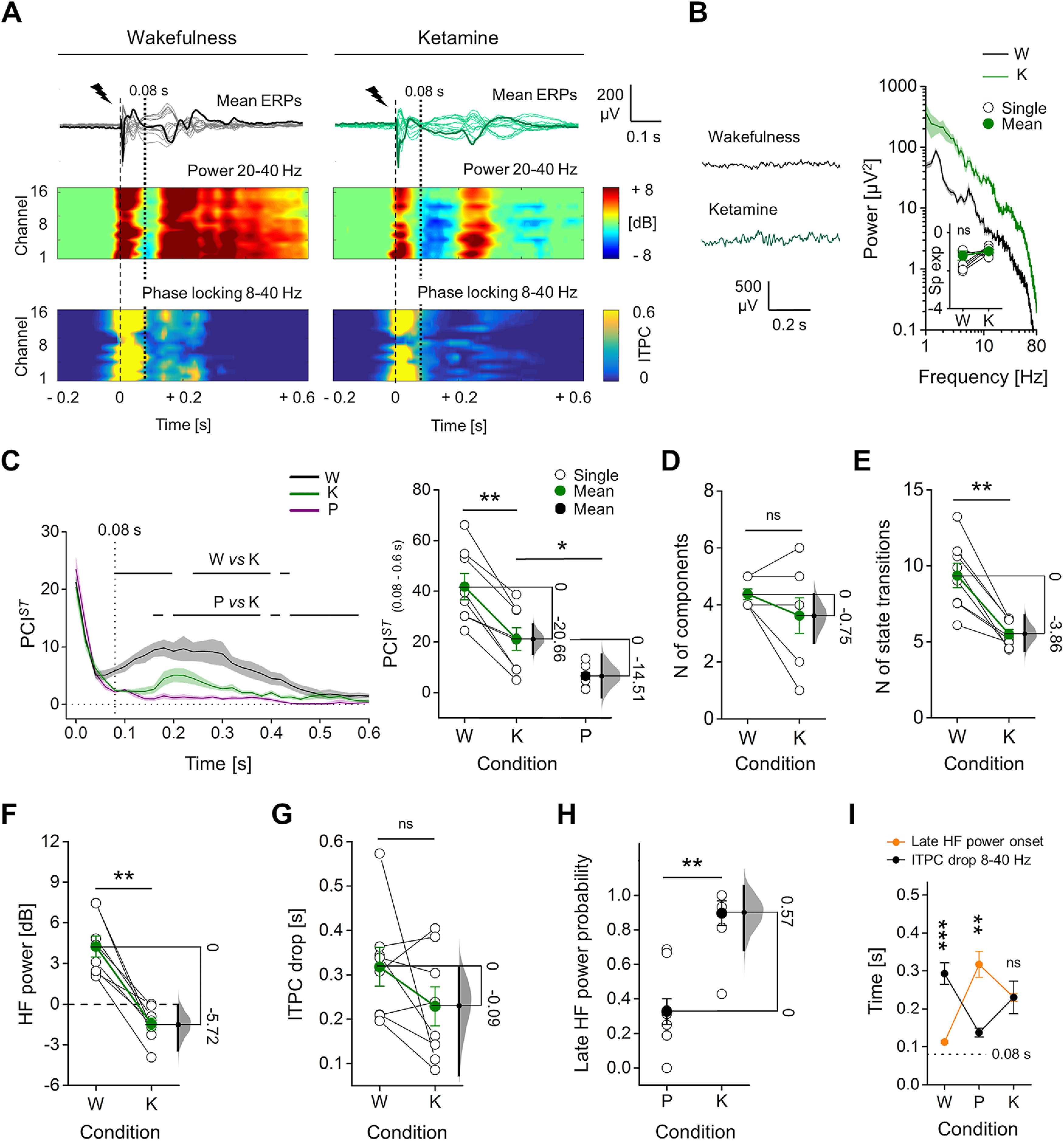
ERPs with ketamine showed intermediate PCI*^ST^*, with HF suppression, but sustained ITPC. ***A***, Mean ERPs from all electrodes in response to single pulse stimulation (1 ms, 50 μA; dashed line) shown superimposed, from the same rat during wakefulness and ketamine anesthesia. One averaged ERP from the same channel (M2) is in bold for clarity. Spectrograms of HF power and ITPC for all channels are shown below. Vertical dotted line at 0.08 s indicates the average time of onset of HF suppression. ***B***, Spontaneous EEG (left) and relative mean periodograms (shades represent SEM; right) are shown from one rat during wakefulness (W) and ketamine anesthesia (K). Spectral exponents from all rats are also shown (inset). ***C***, left, Time courses of mean PCI*^ST^* in wakefulness, ketamine, and propofol (P) anesthesia (shades represent SEM; horizontal lines indicate periods of statistical difference, *p *<* *0.05). Right, PCI*^ST^*in range 0.08–0.6 s is shown for each rat. Propofol data are the same as in Figure 1. ***D***, Number of principal components and ***E***, average state transitions of EEG response are shown for all rats. Mean HF power (in range 0.08–0.18 s; ***F***) and time of ITPC drop averaged (***G***) across channels are shown for all animals during wakefulness and ketamine anesthesia. ***H***, Ratio between the number of electrodes (channels) with a late increase in HF power (after 0.08 s) and the total number of channels. ***I***, Temporal differences between the onset of the late HF power and ITPC drop are shown. The floating axis on the right of each simple group comparison (panels ***C***, right, to ***H***) shows the mean difference between conditions. The effect size is reported as bootstrap resampling distribution of mean difference, with 95% CI represented by the bold black vertical line. See Extended Data Figure 4-1 for in depth comparison with sevoflurane anesthesia.

10.1523/ENEURO.0343-20.2021.f1-4 Extended Data Figure 1-4 PCI*^ST^* can be decomposed in average NST and number of principal components (N_C_). ***A***, ERPs and corresponding PCI*^ST^* decomposition into principal components and state transitions (NST) during wakefulness and during propofol, ketamine and sevoflurane anesthesia (top to bottom) from one animal. Left, Butterfly plots with evoked responses to electrical stimulation of right M2. Right, PCI*^ST^* can be decomposed as the product between the number of principal components (N_C_), an estimate of the spatial diversity of the signal, and the average NST, corresponding to the temporal differentiation of the signal, i.e., PCI*^ST^* = average NST x NC. The panel shows each principal component of the ERPs that together account for 99% of the variance in the response (time range: 0.08–0.6 s), with the corresponding values of state transitions in the legend box; above it, the PCI*^ST^* value computed as the product of average NST and number of principal components (N_C_). ***B***, Scatter plots with average NST and number of principal components for all animals with corresponding group average, and 5 and 95 percentiles (N_C_). Left, Rats during wake (*n* = 9), propofol (*n* = 9), and sevoflurane (*n* = 10). Right, Rats during wake (*n* = 8) and ketamine (*n* = 8). Also shown for reference are the contour lines (dotted gray lines) with different PCI*^ST^* values. Download Figure 1-4, TIF file.

We performed electrophysiological recordings in nine rats during wakefulness and propofol anesthesia (∼1.1 mg/kg/min, i.v.) at a depth that produced spontaneous, slow, high-amplitude EEG oscillations and was sufficient to abolish any detectable motor response to pain stimuli. The redistribution of EEG power from high to low frequencies was confirmed by a reduced spectral exponent of the periodrogram (range: 20–40 Hz) from wakefulness to propofol anesthesia (wakefulness: −1.44 ± 0.12, propofol: −3.12 ± 0.09; Wilcoxon S-R test, *p *=* *0.004; Fig. 1*B*). During wakefulness, single pulse stimulation (1 ms, 50 μA) triggered long-lasting ERPs, including an early, fast, high-voltage response followed by multiple changes in polarity over time and across cortical areas. During anesthesia, however, the same stimulation produced only a similar initial activation, followed by fewer polarity changes (Fig. 1*C*,*D*). The ERP complexity was quantified by PCI*^ST^*, a version of PCI based on the state transitions of principal components of the EEG response (Comolatti et al., 2019; Fig. 1*E*; see Materials and Methods). We initially assessed the PCI*^ST^* time course, using sliding windows of 0.1 s. Immediately after stimulation, PCI*^ST^* was similar across conditions and quickly decayed. Soon afterward, however, complexity (PCI*^ST^*) built up reaching a maximum at 0.21 ± 0.04 s during wakefulness, and differed significantly from propofol anesthesia starting from 0.08 s until the ERP ended (Wilcoxon S-R test; from 0.62 s, the PCI*^ST^*values alternated between being significantly different and not significantly different, while from 0.84 s, we found no further period with statistically significant differences until 1.1 s, with the exception of a transient difference at 0.94 s; Fig. 1*F*). Thus, in the time window 0.08–0.6 s, PCI*^ST^*showed a clear reduction from wakefulness to propofol anesthesia, for both single rats and the population (wakefulness: 42.35 ± 3.47, propofol: 6.63 ± 1.38; mean difference = −35.7 [95% CI: −41.16, −29.93]; Wilcoxon S-R test, *p *=* *0.004; Fig. 1*F*). In the same time range, the reduced PCI*^ST^*was determined both by a reduced number of principal components (wakefulness: 4.44 ± 0.18, propofol: 1.55 ± 0.24; mean difference = −2.89 [95% CI: −3.44, −2.33]; Wilcoxon S-R test, *p *=* *0.004; Fig. 1*G*) and a reduced NST over time (wakefulness: 9.42 ± 0.48, propofol: 4.14 ± 0.57; mean difference = −5.28 [95% CI: −6.25, −4.46]; Wilcoxon S-R test, *p *=* *0.004; Fig. 1*H*; Extended Data Fig. 1-4).

### A period of HF suppression preceded the early interruption of deterministic response during propofol anesthesia

Next, we quantified the changes in spectral power caused by the stimulation, within a HF range (20–40 Hz) that has been shown to maximize the difference between active and silent periods of a neuronal network that oscillates between depolarized and hyperpolarized states (Steriade et al., 1993, 1996, 2001; Mukovski et al., 2007), as typically occurs during NREM sleep and general anesthesia (Steriade et al., 1993, 2001; Volgushev et al., 2006). We also considered the responses as “deterministic,” i.e., reliably driven by the stimulation, if the evoked potentials were largely reproducible in phase when the same stimulation was repeated (David et al., 2006). Consequently, the duration of the deterministic neuronal response was defined as the last time point of the phase-locked component of the ERP measured as ITPC (Cohen, 2014), in 8- to 40-Hz frequency range (Pigorini et al., 2015; D’Andola et al., 2018; Rosanova et al., 2018).

Coherently with the PCI*^ST^*time course, the ERP showed an early, transient increase in HF power during both wakefulness and propofol anesthesia, which was followed by a SC that corresponded to a transient increase in LF power (1–4 Hz; Fig. 2*A*; Extended Data Figs. 2-1, 2-2). A later HF activation, sustained until the end of the response in all channels, was detected only during wakefulness. By contrast, propofol anesthesia induced a deep HF suppression in most of the channels at 0.08 ± 0.01 s (Fig. 2*A*,*B*). When averaging the relative HF power during the suppression (0.08–0.18 s), across channels and rats, we found that it decreased from wakefulness to propofol anesthesia, to values below the baseline in all rats (wakefulness: 4.13 ± 0.59 dB, propofol: −0.79 ± 0.25 dB; mean difference = −4.92 [95% CI: −6.68, −3.77]; Wilcoxon S-R test, *p *=* *0.004; Fig. 2*C*). Traces of HF suppression after stimulation were observed also during wakefulness, but these were briefer and shallower than with propofol (Extended Data Fig. 2-3), and were not seen after averaging (Fig. 2*B*,*C*). Moreover, during wakefulness the stimulation evoked durable, phase-locked responses in all electrodes. In contrast, during propofol anesthesia the period with HF suppression preceded an earlier drop of ITPC in all channels, followed by transient and not phase-locked HF activations in few cortical areas (Fig. 2*A*,*B*). Consequently, the phase-locked response, measured by averaging the ITPC drop time across channels and rats, was significantly briefer during propofol (0.13 ± 0.01 s) than during wakefulness (0.32 ± 0.04 s; mean difference = −0.19 [95% CI: −0.25, −0.14]; Wilcoxon S-R test, *p *=* *0.004; Fig. 2*D*).

10.1523/ENEURO.0343-20.2021.f2-1 Extended Data Figure 2-1 Single trials and SC of the ERP during propofol anesthesia. Example of raw EEG responses to single pulse electrical stimulation (1 ms, 50 μA; dashed line at 0 s; ***A***) and corresponding low-passed responses (<4 Hz, Butterworth filter, 3^rd^ order; right; ***B***) from one rat during propofol anesthesia. The mean ERP (bold) and all 90 single trials from the same frontal channel (M2) are overlaid and shown at the top, while 11 consecutive single trials are shown below for clarity. The responses are from the same animal and channel as in Figure 2. The low-passed responses are used to visualize and quantify the SC (<4 Hz) of the ERP, which was consistently evoked by the stimulus, in nine rats. ***C***, The spontaneous slow wave activity before the stimulation might influence the SC of the response, since we found a linear, strong, positive correlation between the amplitude of the spontaneous SC and the amplitude of the evoked SC (linear fit, *p *=* *3.562 × 10^−5^, *R*^2^ = 0.914). The amplitude of the spontaneous SC (sp SC) for each single trial was quantified with the rms of 2-s baseline (range –2 to 0 s). The values of single trials were then averaged across trials and channels, obtaining estimates for all rats, which were used for assessing the correlation. The same procedure was adopted to quantify the amplitude of the evoked SC (ev SC), within a shorter time range after stimulation (from 0 to 0.3 s) to only include the evoked response. ***D***, The evoked SC was slightly, but significantly higher in amplitude than the spontaneous SC (sp SC: 24.88 ± 2.09 μV, ev SC: 26.76 ± 2.40 μV; mean difference = 1.88 [95% CI: 0.83, 3.5]; Wilcoxon S-R test, *p *=* *0.019). ***E***, Consistently, the maximal peak amplitude of the evoked SC (SC max) was linearly and positively correlated with the maximal increase of LF power (1–4 Hz) with respect to baseline (linear fit, *p *=* *0.046, *R*^2^ = 0.454). SC max was computed for each rat, from the mean low-passed ERP of each channel, as the maximal absolute amplitude value after the stimulation (time range 0–0.6 s), and then averaged across channels. While the maximal LF power was obtained from the relative spectral powers of the ERP averaged in LF range (1–4 Hz; wavelet convolution, see Materials and Methods) and was the maximal positive value of relative LF power after the stimulation (max dB > 0 in time range 0–0.6 s), then averaged across channels. ***F***, The latency from stimulation of SC max was also positively correlated with the time point of the maximal ITPC value in LF range (1–4 Hz, LF ITPC max), averaged across channels (linear fit, *p *=* *0.034, *R*^2^ = 0.495). This indicated a deterministic relation between the stimulus and the SC of the ERP. ***G***, The mean onset time of the SC max across channels was found to be significantly later than the starting time of the HF suppression period (20–40 Hz, dB < 0) of the ERP, averaged across channels (SC max: 0.13 ± 0.02 s, HF sup. start: 0.08 ± 0.01 s; mean difference = –0.05 [95% CI: –0.10, –0.02]; Wilcoxon S-R test, *p *=* *0.027). ***H***, The mean latency of SC max was still later, but closer to the onset time of the maximal HF suppression (most negative dB peak of averaged power in ranges 20–40 Hz, 0–0.3 s), and the statistical difference between these timings was slightly below the threshold for significance (SC max: 0.13 ± 0.02 s, HF sup. max: 0.09 ± 0.01 s; mean difference = –0.036 [95% CI: –0.08, –0.01]; Wilcoxon S-R test, *p *=* *0.055). ***I***, Finally, on average across channels and rats, the onset time of SC max was almost coincident with the end time of the HF suppression period (last time point with mean power < 0 dB, in ranges 20–40 Hz, 0–0.3 s) , and no statistical difference was detected (SC max: 0.13 ± 0.02 s, HF sup. end: 0.13 ± 0.01 s; mean difference = 0.002 [95% CI: –0.03, 0.02]; Wilcoxon S-R test, *p *=* *0.652). The floating axis on the right of each group comparison (panels ***D***, ***G***, ***H***, ***I***) shows the mean difference between conditions. The effect size is reported as bootstrap resampling distribution of mean difference, with 95% CI represented by the bold black vertical line. Download Figure 2-1, TIF file.

10.1523/ENEURO.0343-20.2021.f2-2 Extended Data Figure 2-2 Single trials and SC of the ERP during wakefulness. Example of raw EEG responses to single pulse electrical stimulation (1 ms, 50 μA; dashed line at 0 s; ***A***) and corresponding low-passed responses (<4 Hz, Butterworth filter, 3rd order; right; ***B***) from one rat during wakefulness. The mean ERP (bold) and all 90 single trials from the same frontal channel (M2) are overlaid and shown at the top, while 11 consecutive single trials are reported below for a better representation. The responses are from the same animal and channel reported in Figure 2. The low-passed responses are used to visualize and quantify the SC (<4 Hz) of the ERP, which was consistently evoked by the stimulus, in nine rats. ***C***, The spontaneous activity before the stimulation might influence the SC of the response, since we found a linear, positive correlation between the amplitude of the spontaneous SC and the amplitude of the evoked SC (linear fit, *p *=* *0.033, *R*^2^ = 0.499). The amplitude of the spontaneous SC (sp SC) for each single trial was quantified with the rms of 2-s baseline (range –2 to 0 s). The values of single trials were then averaged across trials and channels, obtaining an estimation for all rats, which were used for assessing the correlation. The same procedure was adopted to quantify the amplitude of the evoked SC (ev SC), within a shorter time range after stimulation (from 0 to 0.3 s) to only include the evoked response. ***D***, The evoked SC was significantly higher in amplitude than the spontaneous SC (sp SC: 16.96 ± 1.20 μV, ev SC: 39.00 ± 4.82 μV; mean difference = 22.0 [95% CI: 15.4, 30.6]; Wilcoxon S-R test, *p *=* *0.004). The floating axis on the right shows the mean difference between conditions and the effect size is reported as bootstrap resampling distribution of mean difference, with 95% CI represented by the bold black vertical line. ***E***, Consistently, the maximal peak amplitude of the evoked SC (SC max) was linearly and positively correlated with the maximal increase of LF power (1–4 Hz) with respect to baseline (linear fit, *p *=* *5.255 × 10^−4^, *R*^2^ = 0.839). SC max was computed for each rat, from the mean low-passed ERP of each channel, as the maximal absolute amplitude value after the stimulation (time range 0–0.6 s), and then averaged across channels. While the maximal LF power was obtained from the relative spectral powers of the ERP, averaged in LF range (1–4 Hz; wavelet convolution, see Materials and Methods), and was the maximal positive value of relative LF power after the stimulation (max dB >0 in time range 0–0.6 s), then averaged across channels. ***F***, However, the latency of SC max from stimulation was not correlated with the time point of the maximal ITPC value in LF range (1–4 Hz, LF ITPC max), averaged across channels (linear fit, *p *=* *0.176, *R*^2^ = 0.245). Download Figure 2-2, TIF file.

10.1523/ENEURO.0343-20.2021.f2-3 Extended Data Figure 2-3 The suppression of HF power during wakefulness was weaker and briefer than the one with propofol, and was not affected by increasing stimulus intensity. ***A***, Epidural EEG activity in response to single pulse electrical stimulation (1 ms, 50 μA; dashed line) of the right M2, from the same rat during wakefulness (up) and during propofol anesthesia (bottom). The electrophysiological traces in the butterfly plot represent the superimposition of ensemble averages of ERPs (*n* = 90 trials) from all recording electrodes (mean ERPs from three channels are in bold for highlighting difference in complexity, same channels between wakefulness and propofol conditions). Below, The temporal dynamic of HF power (averaged in the range 20–40 Hz) from all the channels is also reported (dotted horizontal line indicates 0 dB). The color maps show the color-coded interpolation of HF power during the suppression period (window of time when HF power is <0 dB) over the scalp in a rostro-caudal orientation (R-C). Orange lines represent the isodistance lines that connect channels with similar spatial distance from the stimulating electrode (orange dot; M2 right). To assess the relation between HF suppression and the spatial distance from the stimulus, we averaged values from channels that were clustered in four isodistance lines, at 1.5 ± 0, 4.38 ± 0.47, 7.8 ± 0.17, and 10.8 ± 0.40 mm far from the site of stimulation. ***B***, Quantification of the negative peak of HF power during suppression (deepest suppression, HF suppression max; left) and duration of the suppression of HF power (right), averaged within isodistance lines and across rats (*n* = 9 animals) during wakefulness (W) and propofol anesthesia (P). During wakefulness, HF suppression max linearly approaches to 0 dB from more negative values while increasing the distance from the site of stimulation (Friedman test, *p *=* *6.022 × 10^−5^; linear fit, *p *=* *0.021, *R*^2^ = 0.959). Also, during propofol anesthesia a relation between HF suppression max and distance from stimulation could be detected (Friedman test, *p *=* *8.694 × 10^−5^), but this was not linear (linear fit, *p *=* *0.103, *R*^2^ = 0.805). Overall, HF suppression max was closer to 0 dB during wakefulness then during propofol anesthesia (Friedman test, *p *=* *4.817 × 10^−6^). Likewise, the duration of HF suppression linearly decreased approaching 0 s by increasing the distance from the stimulus site during wakefulness (Friedman test, *p *=* *2.458 × 10^−5^; linear fit, *p *=* *0.001, *R*^2^ = 0.997). A relation between HF suppression duration and distance from stimulus could be also detected during propofol anesthesia, but this was not linear (Friedman test, *p *=* *2.734 × 10^−4^; linear fit, *p *=* *0.213, *R*^2^ = 0.618). Overall, HF suppression duration was shorter during wakefulness then during propofol anesthesia (Friedman test, *p *=* *2.205 × 10^−4^). ***C***, In addition, both HF suppression max (left) and HF suppression duration (right; averaged across all channels) were found to change differently in relation to the intensity of stimulation between wakefulness and propofol anesthesia in *n* = 5 rats. No significant trends were found during wakefulness (HF suppression max, Friedman test, *p *=* *0.145; HF suppression duration, Friedman test, *p *=* *0.472). Otherwise, during propofol anesthesia, HF suppression max significantly decreased by increasing intensity of stimulation with a trend that could be linearly fitted (Friedman test, *p *=* *0.007; linear fit, *p *=* *0.035, *R*^2^ = 0.932) and HF suppression duration linearly increased by increasing stimulus intensity (Friedman test, *p *=* *0.005; linear fit, *p *=* *0.024, *R*^2^ = 0.953). Overall, also in relation to stimulus intensity, HF suppression max was lower and HF suppression duration was higher during propofol anesthesia than during wakefulness (HF suppression max, Friedman test, *p *=* *0.037; HF suppression duration, Friedman test, *p *=* *0.003). Download Figure 2-3, TIF file.

Next, we increased the stimulation intensity from 40 to 100 μA (1 ms; five rats), attempting to compensate for the inhibiting effect of propofol (Ouyang et al., 2003; Bieda and MacIver, 2004; Fig. 3*A*). The resulting neuronal excitation, quantified by the rms amplitude of the first deflections of mean ERPs (early ERP rms, up to 0.05 s), increased linearly with stimulus intensity, during both wakefulness (Friedman test, *p *=* *0.002; linear fit, *p *=* *0.006, *R*^2^ = 0.988; Fig. 3*B*) and propofol anesthesia (Friedman test, *p *=* *0.002; linear fit, *p *=* *0.016, *R*^2^ = 0.968). Overall, no significant differences in Early ERP rms were detected between wakefulness and anesthesia (Friedman test, *p *=* *0.917). With propofol, the increased excitation was accompanied by a deeper HF suppression as indicated by the linear decrease of HF power as function of stimulus intensity (Friedman test, *p *=* *0.005; linear fit, *p *=* *0.015, *R*^2^ = 0.969; Fig. 3*A*,*C*) and the most negative peak within the period of HF suppression (HF suppression max) highly correlated in magnitude with the maximal amplitude of the SC of the ERP (SC max; Fig. 3*D*; linear fit, *p *=* *2.809 × 10^−4^, *R*^2^ = 0.529; average values across channels for each rat and stimulus intensity). In contrast, during wakefulness, the mean HF power was always above baseline, thus higher than during propofol (Friedman test, *p *=* *1.767 × 10^−7^; Fig. 3*C*), and no change with stimulus intensity was detected (Friedman test, *p *=* *0.178; Fig. 3*C*). During propofol anesthesia, higher stimulus intensities were also linearly related to prolonged periods of HF suppression (Fig. 3*A*; Extended Data Fig. 2-3; Friedman test, *p *=* *0.005; linear fit, *p *=* *0.024, *R*^2^ = 0.953). In contrast, we did not find intensity dependent change in the brief HF suppressions during wakefulness (Extended Data Fig. 2-3). We then used the changes in duration seen with propofol to assess a temporal relation between the interruption of ITPC and HF suppression. We found a first significant correlation with the latency of HF suppression max (linear fit, *p *=* *0.043, *R*^2^ = 0.209; average values across channels for each rat and stimulus intensity; Fig. 3*E*) and a stronger temporal correlation with the end of HF suppression (linear fit, *p *=* *0.008, *R*^2^ = 0.333; average values across channels for each rat and stimulus intensity; Extended Data Fig. 3-1). Interestingly, on average across channels and rats, the end time point of HF suppression was coincident with the latency of SC max (Extended Data Fig. 2-1). Finally, PCI*^ST^* was always higher and ITPC more long-lasting during wakefulness than with propofol, regardless of the stimulus intensity, in all tested animals (Extended Data Fig. 3-2).

10.1523/ENEURO.0343-20.2021.f3-1 Extended Data Figure 3-1 ITPC drop time better correlated with the end phase of HF suppression. ***A***, Mean ERPs from the same rat in response to single pulse electrical stimulations (1 ms, 60 μA; dashed lines) during propofol anesthesia (up), with corresponding phase-locking temporal dynamics averaged in broad frequency range, 8–40 Hz (ITPC; middle), and temporal dynamic of HF power, averaged in range 20–40 Hz (bottom). The three plots report the superimposed activity from all 16 recording electrodes, with the dynamics from the same parietal channel (RS) in bold for clarity. Letters B, C, D, and E, respectively, approximate the onset of the first HF power <0 dB (HF suppression, start), the latency of the most negative peak of HF power (HF suppression, max), the time point corresponding to the 75% of the HF suppression duration (HF suppression, 75% duration), and the last time point of HF power <0 dB (HF suppression, end). These time points have been calculated within the time range 0–0.3 s, averaged across channels for each rat (*n* = 5) and stimulation intensity (40, 60, 80, 100 μA), and plotted against the respective mean ITPC drop time to assess correlations in panels ***B****–****E*** (values from different stimulus intensities are color coded). ***B***, The ITPC drop time did not correlate with the onset of HF suppression (linear fit, *p *=* *0.312, *R*^2^ = 0.057). However, linear positive correlations were found with HF suppression, max (***C***; linear fit, *p *=* *0.043, *R*^2^ = 0.209), with HF suppression, 75% duration (***D***; *p *=* *0.023, *R*^2^ = 0.257) and with the HF suppression, end (***E***; *p *=* *0.008, *R*^2^ = 0.333). It should be noted how both the statistical significance and the coefficient of determination gradually increased by assessing the correlation of ITPC drop time against later time points of HF suppression (lower *p* and higher *R*^2^). Thus, the best temporal correlation was between the ITPC drop and the end of the HF suppression. Download Figure 3-1, TIF file.

10.1523/ENEURO.0343-20.2021.f3-2 Extended Data Figure 3-2 Perturbational complexity in relation to incremental intensity of stimulation. ***A***, epidural EEG activity in response to single pulse electrical stimulations of increasing intensities (1 ms, 40, 60, 80, and 100 μA; dashed line) of the right M2 from the same rat during wakefulness (up) and during the exposure to propofol anesthesia (middle). The electrophysiological traces represent the superimposition of ensemble averages of evoked related potentials from all the 16 recording electrodes distributed all over the skull. One averaged ERP from the same channel across condition has been highlighted in bold for better illustrating the difference in cortical complexity. The vertical dotted lines indicate 0.08 s, which approximates the rising of the HF suppression duration. PCI*^ST^* scores obtained from the whole window of the evoked response (0–0.6 s) are shown for all animals (white dots) and averaged across rats (*n* = 5 rats; purple dots) for all intensities of stimulation and condition (below). PCI*^ST^* always decreased in all animals, from wakefulness (W) to propofol anesthesia (P), regardless of the intensity of stimulation. The floating axis on the right of each group comparison shows the mean difference between conditions. The effect size is reported as bootstrap resampling distribution of mean difference, with 95% CI represented by the bold black vertical line. ***B***, We otherwise observed a significant variation of PCI*^ST^* (in full-time window 0–0.6 s; left) during propofol anesthesia in relation to stimulus intensity (Friedman test, *p *=* *0.029), which was not detectable during wakefulness (Friedman test, *p *=* *0.062). Overall PCI*^ST^* during wakefulness was higher than during propofol anesthesia (Friedman test, *p *=* *1.55 × 10^−5^). No relation with stimulus intensity was detected by calculating PCI*^ST^*from 0.08 to 0.6 s and therefore by excluding the first response to the electrical stimulation, before the rising of the HF suppression (right). No significant variation of PCI*^ST^* has been observed during propofol anesthesia (Friedman test, *p *=* *0.782), but a dim variation seemed to be present during wakefulness even if it did not reach statistical significance (Friedman test, *p *=* *0.0503). Overall perturbational complexity during wakefulness was higher than during propofol anesthesia (Friedman test, *p *=* *1.767 × 10^−7^). ***C***, Quantification of ITPC drop time (8–40 Hz; right) as a function of increasing intensity of stimulation during both wakefulness and propofol anesthesia. Values are averaged across channels and animals. No statistically significant variation was detected in wakefulness condition (Friedman test, *p *=* *0.077). Otherwise, during propofol anesthesia, we identified a significant variation of ITPC drop time (Friedman test, *p *=* *0.02) as a function of stimulus intensity. This was in line with the correlation observed with the end of the HF suppression duration (see Extended Data Fig. 3-1). Overall, ITPC drop time values obtained during wakefulness were higher than during propofol anesthesia (ITPC drop, Friedman test, *p *=* *7.082 × 10^−6^). ***D***, The quantification of the amplitude of a later deflection of ERPs in relation to stimulus intensity, between wakefulness and anesthesia is reported. RMS amplitude has been measured around 0.2 s (in time window 0.175–0.225 s, black arrowheads in ***a***) of the mean ERP from all channels and then averaged for each animal (*n* = 5 rats) and condition. As for the early evoked response (see Fig. 3), a linear increase of the amplitude of the later deflection of ERPs has been detected as a function of stimulus intensity in wakefulness (Friedman test, *p *=* *0.013; linear fit, *p *=* *0.032, *R*^2^ = 0.936). Also, in propofol anesthesia, the amplitude of the later deflection of the ERPs changed as a function of stimulus intensity, but it was not linear (Friedman test, *p *=* *0.04; linear fit, *p *=* *0.14, *R*^2^ = 0.739). Overall, differently from the early evoked response (Fig. 3), the amplitude of the later deflection of ERPs has been found to be significantly higher in wakefulness than during propofol anesthesia (Friedman test, *p *=* *1.767 × 10^−7^). Download Figure 3-2, TIF file.

### ERPs during ketamine anesthesia showed intermediate complexity

In order to test whether the PCI*^ST^* reduction with propofol might be related to behavioral unresponsiveness per se, we repeated single pulse stimulations (1 ms, 50 μA; eight rats) during ketamine anesthesia, which was found to maintain high brain complexity in humans (Sarasso et al., 2015), at a dose that abolished all motor responses to painful stimuli (∼1.8 mg/kg/min i.v.; Fig. 4*A*). Similarly to wakefulness, the spontaneous EEG activity with ketamine showed fast, shallow oscillations, with similar spectral exponent (seven rats; wakefulness: −1.21 ± 0.24, ketamine: −0.97 ± 0.09; Wilcoxon S-R test, *p *=* *0.156; Fig. 4*B*). The PCI*^ST^* time course revealed a similar initial complexity of the ERP across conditions that quickly decayed. Like wakefulness, but unlike propofol, during ketamine anesthesia PCI*^ST^* increased soon after the initial decay, reaching a peak at ∼0.2 s and gradually decreased until the ERP end. From 0.08 s the complexity level with ketamine was lower than during wakefulness, but transient periods of similar PCI*^ST^* were detected (Wilcoxon S-R test; from 0.44 s we found no significant differences between wakefulness and ketamine conditions until 1.1 s, except an isolated moment of difference at 0.62 s; Fig. 4*C*). Similarly, from 0.16 s, PCI*^ST^* with ketamine was significantly higher than during propofol anesthesia and only brief periods of similar complexity were identified until the end of ERP (Mann–Whitney test; Fig. 4*C*). Coherently, PCI*^ST^* differed between wakefulness and ketamine anesthesia for the 0.08–0.6 s period (wakefulness: 41.80 ± 5.17, ketamine: 21.14 ± 4.48; mean difference = −20.66 [95% CI: −26.60, −14.80]; Wilcoxon S-R test, *p *=* *0.008; Fig. 4*C*), but also between ketamine and propofol anesthesia (mean difference = −14.51 [95% CI: −23.02, −6.13]; Mann–Whitney test, *p *=* *0.024; Fig. 4*C*). The intermediate PCI*^ST^* with ketamine was explained by a similar number of ERP components compared with wakefulness (wakefulness: 4.37 ± 0.18, ketamine: 3.62 ± 0.62; mean difference = −0.75 [95% CI: −1.87, 0]; Wilcoxon S-R test, *p *=* *0.25; Fig. 4*D*), with a reduced NST (wakefulness: 9.36 ± 0.81, ketamine: 5.50 ± 0.31; mean difference = −3.86 [95% CI: −5.00, −2.63]; Wilcoxon S-R test, *p *=* *0.008; Fig. 4*E*; see also Extended Data Fig. 1-4). After the similarly complex initial response, also with ketamine a period of HF suppression occurred at 0.08 ± 0.01 s (wakefulness: 4.25 ± 0.78 dB, ketamine: −1.47 ± 0.44 dB, mean difference = −5.72 [95% CI: −7.71, −4.25]; Wilcoxon S-R test, *p *=* *0.008; Fig. 4*F*), but no consistent changes in ITPC drop time were found (wakefulness: 0.32 ± 0.04 s, ketamine: 0.23 ± 0.04 s; mean difference = −0.09 [95% CI: −0.25, 0]; Wilcoxon S-R test, *p *=* *0.195; Fig. 4*G*). After the period of HF suppression, a late increase in HF power occurred in a higher proportion of channels during ketamine anesthesia than with propofol (propofol, 0.33 ± 0.07; ketamine, 0.90 ± 0.07; mean difference = 0.57 [95% CI: 0.35, 0.72]; Mann–Whitney test, *p *=**0.001; Fig. 4*H*), while in wakefulness, it was seen in all channels in all 12 animals. We then assessed whether this resumption of HF activity was still associated with the deterministic neuronal activation by comparing the time of ITPC drop with the onset of late HF power increment. Only channels that presented a late increase in HF power were included for this comparison. During wakefulness, the resumption of HF activity was largely deterministic, occurring within a period of significant ITPC (Wilcoxon S-R test, *p *=* *4.883 × 10^−4^; Fig. 4*I*), whereas during propofol the late HF power was not phase-locked as it occurred after the fading of ITPC (Wilcoxon S-R test, propofol, *p *=* *0.008; Fig. 4*I*). Coherently with the intermediate complexity level during ketamine, the late HF activity observed in this condition occurred with a variable relationship with respect to the drop of ITPC (Wilcoxon S-R test, *p *=* *1; Fig. 4*I*).

10.1523/ENEURO.0343-20.2021.f4-1 Extended Data Figure 4-1 PCI*^ST^*, suppression of high frequencies and phase-locking of ERPs in response to electrical stimulation during sevoflurane anesthesia. ***A***, Superimposition of mean ERPs from all electrodes in response to single pulse stimulation (1 ms, 50 μA; dashed line) from the same rat during wakefulness (W) and sevoflurane (S; ∼2.6%) anesthesia (same rat from Fig. 4 to facilitate comparisons). One averaged ERP from the same channel (M2) is in bold to highlight differences in complexity. Spectrograms of HF power and ITPC for all channels are shown below. Vertical dotted line at 0.08 s indicates the average time of HF suppression onset across rats. ***B***, Spontaneous EEG (left) and relative mean periodograms (shades represent SEM; right) are reported from one rat during wakefulness and sevoflurane anesthesia. Spectral exponents from all rats are also reported (inset). The spectral exponent dropped from wakefulness to sevoflurane anesthesia, highlighting a redistribution of power towards slow frequencies (*n* = 9 rats; wakefulness: –1.71 ± 0.15, sevoflurane: –2.81 ± 0.04; Wilcoxon S-R test, *p *=* *0.004). ***C***, left, Time courses of mean PCI*^ST^* in conditions of wakefulness and sevoflurane anesthesia (*n* = 10 rats; shades represent SEM; horizontal lines indicate time periods with statistical differences between conditions, Wilcoxon S-R test *p *<* *0.05; from 0.58 s, the PCI*^ST^*values alternated between being significantly different and not significantly different between conditions, while from 0.8 s, we found no further period with statistically significant differences until 1.1 s). Right, PCI*^ST^*in range 0.08–0.6 s is reported for each rat and condition. PCI*^ST^*_(0.08–0.6 s)_ significantly dropped from wakefulness to sevoflurane anesthesia (wakefulness: 32.50 ± 4.60, sevoflurane: 8.57 ± 1.85; mean difference = –23.93 [95% CI: –31.79, –16.47]; Wilcoxon S-R test, *p *=* *0.002). Moreover with sevoflurane, PCI*^ST^*_(0.08–0.6 s)_ was similar to what we obtained with propofol, but significantly lower compared to the ketamine condition (Mann–Whitney test; propofol vs sevoflurane, *p *=* *0.775; ketamine vs sevoflurane, *p *=* *0.018). Like for propofol anesthesia, the decreased PCI*^ST^*_(0.08–0.6 s)_ compared to wakefulness was explained by both a reduced number of principal components (***D***, wakefulness: 3.80 ± 0.42, sevoflurane: 1.80 ± 0.29; mean difference = –2 [95% CI: –2.6, –1.4]; Wilcoxon S-R test, *p *=* *0.004) and reduced averaged NST (***E***, wakefulness: 8.33 ± 0.47, sevoflurane: 4.38 ± 0.36; mean difference = –3.95 [95% CI: –5.11, –2.69]; Wilcoxon S-R test, *p *=* *0.002) of the EEG response to the stimulation. ***F***, During sevoflurane anesthesia, the electrical stimulation triggered a first response followed by a profound HF suppression (20–40 Hz) in all animal tested that started at 0.075 ± 0.005 s and ended at 0.132 ± 0.008 s (average across channels and rats). The latencies of the start and of the end of HF suppression were similar to what observed with propofol (Mann–Whitney test; HF suppression start, propofol vs sevoflurane, *p *=* *0.596; HF suppression end, propofol vs sevoflurane, *p *=* *1). By averaging HF power across channels, in time range 0.08–0.18 s, a significant difference from wakefulness was detected (wakefulness: 4.01 ± 0.81 dB, sevoflurane: –1.04 ± 0.40 dB, mean difference = –5.06 [95% CI: –7.62, –3.28]; Wilcoxon S-R test, *p *=* *0.002), but not from propofol condition (Mann–Whitney test; propofol vs sevoflurane, *p *=* *0.838). ***G***, The mean ITPC drop time (8–40 Hz) across channels also significantly decreased from wakefulness to sevoflurane anesthesia (wakefulness: 0.27 ± 0.03 s, sevoflurane: 0.12 ± 0.01 s; mean difference = –0.15 [95% CI: –0.23, –0.11]; Wilcoxon S-R test, *p *=* *0.002) indicating shorter phase-locked ERPs in the latter condition, similarly to what seen with propofol (Mann–Whitney test; ITPC drop time, propofol vs sevoflurane, *p *=* *0.713). ***H***, With sevoflurane, the ratio between the amount of channels that presented a later increase in HF power and the total number of electrodes (late HF power probability, 0.45 ± 0.10) was similar to what observed with propofol, and significantly lower than during ketamine anesthesia (Mann–Whitney test, sevoflurane vs propofol, *p *=* *0.46; sevoflurane vs ketamine, *p *=* *0.006). ***I***, Timings of late HF power onset and ITPC drop (8–40 Hz) are shown for all conditions. Like for propofol anesthesia, with sevoflurane the increment of HF power occurred later then ITPC drop time in 8- to 40-Hz range (Wilcoxon S-R test, sevoflurane *p *=* *0.002), thus indicating a not phase-locked activity. Propofol and ketamine data are the same from Figure 4, reported here to allow comparisons. The floating axis on the right of each simple group comparison (panels ***C*,** middle, to ***G***) shows the mean difference between conditions. The effect size is reported as bootstrap resampling distribution of mean difference, with 95% CI represented by the bold black vertical line. Download Figure 4-1, TIF file.

We also used another general anesthetic, the volatile sevoflurane, to test whether periods of HF suppression combined with reduced ITPC and low PCI*^ST^* were not specific to propofol. Sevoflurane anesthesia in 10 rats produced results that resembled what we observed with propofol. Thus, the PCI*^ST^* value was lower than during wakefulness and ketamine anesthesia, but no statistical difference was found when compared with propofol, and this low PCI*^ST^* was explained by a reduced number of both principal ERP components and state transitions compared with wakefulness. A period of deep HF suppression (−1.04 ± 0.40 dB) occurred at 0.075 ± 0.005 s from stimulation and the phase-locked response dropped soon afterward, at 0.12 ± 0.01 s. As for propofol and differently from wakefulness and ketamine conditions, only few channels showed later increments of HF power, which were consistently not phase-locked (Extended Data Figs. 1-4, 4-1).

### Functional connectivity and diversity of response were conserved during wakefulness and ketamine anesthesia but collapsed with propofol and sevoflurane

**Figure 5.**
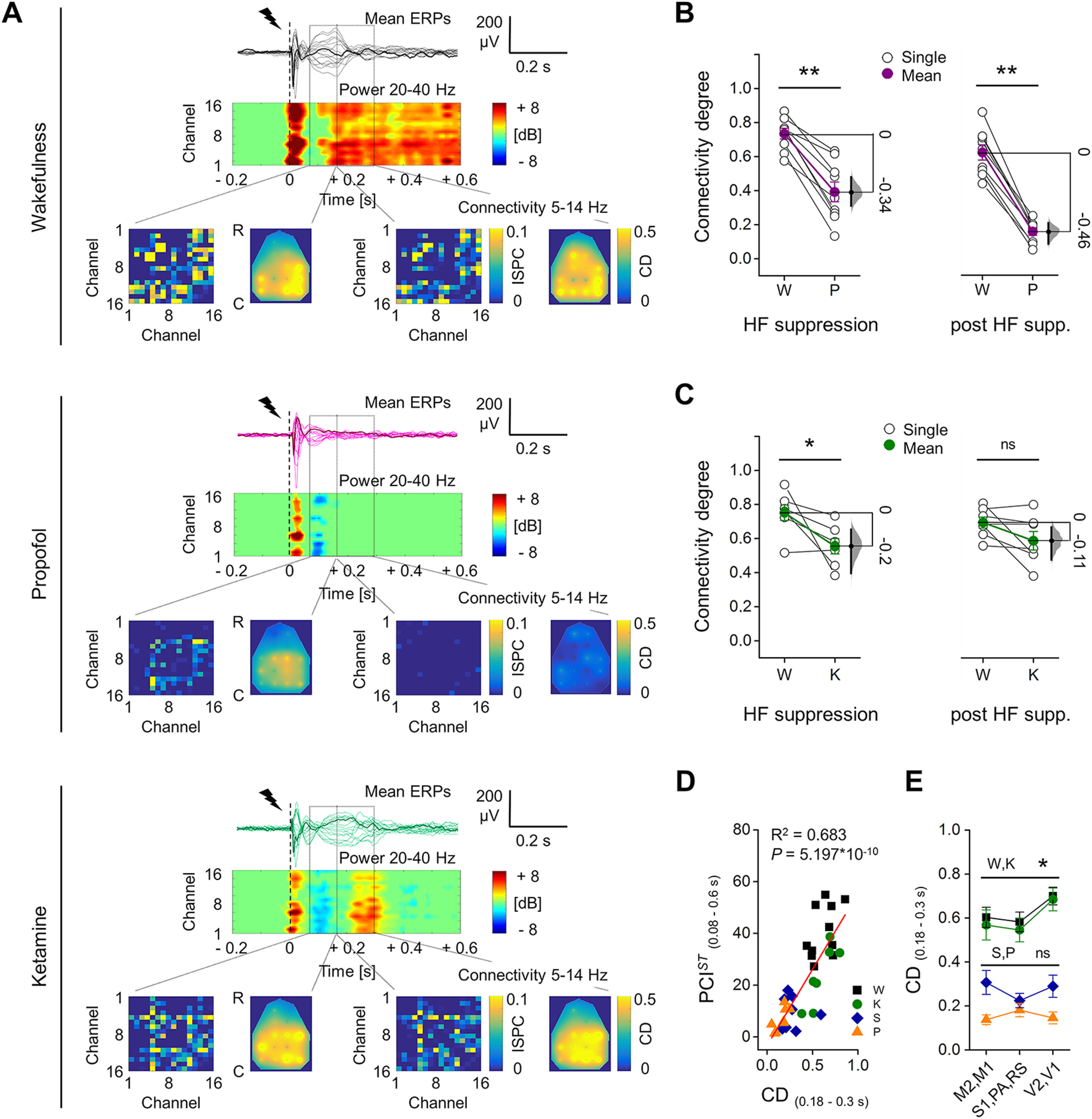
Functional cortical connectivity after perturbation was reduced during propofol or sevoflurane anesthesia compared with wakefulness, while was conserved with ketamine. ***A***, Superimposition of mean ERPs from all electrodes in response to single pulse stimulation (1 ms, 50 μA; dashed line) from the same rat during wakefulness (W) and propofol (P) and ketamine (K) anesthesia (from up to bottom). One averaged ERP is shown in bold for clarity. Spectrograms of HF power are shown for each channel, below the butterfly plots. The bottom part of each inner panel reports increments in functional connectivity compared with baseline in two time windows: during HF suppression, 0.08–0.18 s (left) and after HF suppression, 0.18–0.3 s (right; rectangles indicate the time windows). For each window, the connectivity matrix based on mean ISPC (5–14 Hz) is reported on the left and the topographical distribution (R-C: rostral-caudal) of CD for each channel is interpolated and shown on the right. ***B***, ***C***, Mean CD across channels during HF suppression (left) and post-HF suppression (right) from rats during wakefulness and propofol (***B***, *n* = 9), and ketamine (***C***, *n* =7) anesthesia. ***D***, Mean CD (range: 0.18–0.3 s) across channels from all animals and conditions are plotted against PCI*^ST^* (range: 0.08–0.6 s) and linearly fitted (coefficient of determination *R*^2^ and *p* value are reported). ***E***, Mean CD (range: 0.18–0.3 s) across rats and across channels organized in three cortical regions are shown for each condition. In ***D***, ***E***, sevoflurane condition (S) is also reported (*n* = 9). The floating axis on the right of each simple group comparison (panels ***B***, ***C***) shows the mean difference between conditions. The effect size is reported as bootstrap resampling distribution of mean difference, with 95% CI represented by the bold black vertical line. See Extended Data Figure 5-1 for analysis of functional connectivity with sevoflurane anesthesia and Extended Data Figure 5-2 for analysis of functional connectivity across conditions for each frequency band of interest.

In principle, PCI estimates integration and differentiation in a neuronal network (Casali et al., 2013; Comolatti et al., 2019). Thus, high PCI value should indicate a highly connected network with diversified connectivity patterns. In order to test this, we assessed the functional connectivity across cortical regions following electrical stimulation by computing the ISPC (Cohen, 2014) for each channel pair, averaged in the θ-α frequency range (5–14 Hz), which includes the frequency bands that showed more long-lasting ITPC in wakefulness (mean ITPC drop time across channels and 12 rats, for each frequency band >4 Hz: θ = 0.34 ± 0.02 s; α = 0.29 ± 0.03 s; β = 0.19 ± 0.02 s; γ = 0.13 ± 0.02 s). We then averaged the resulting ISPC in two time windows of interest: during the HF suppression (0.08–0.18 s) and afterward, until the mean ITPC drop time in wakefulness, post-HF suppression (0.18–0.3 s). We considered the increase in ISPC above the baseline and computed the CD for each electrode, as the ratio between the number of significantly synchronized channel pairs over the total number of channels (Fig. 5*A*). During wakefulness, the averaged CD was sustained up to 0.3 s and was higher than during propofol anesthesia in both time windows (nine rats; HF suppression, wakefulness: 0.73 ± 0.03, propofol: 0.39 ± 0.06, mean difference = −0.34 [95% CI: −0.42, −0.25]; post-HF suppression, wakefulness: 0.62 ± 0.04, propofol: 0.16 ± 0.02, mean difference = −0.46 [95% CI: −0.54, −0.41]; Wilcoxon S-R test, HF suppression: *p *= 0.002; post-HF suppression: *p *=* *0.004; Fig. 5*B*). Sevoflurane gave similar results (nine rats; Extended Data Fig. 5-1). In contrast, ketamine anesthesia induced a significant drop of CD compared with wakefulness only during the HF suppression (seven rats; wakefulness: 0.75 ± 0.04, ketamine: 0.55 ± 0.04; mean difference = −0.20 [95% CI: −0.36, −0.10]; Wilcoxon S-R test, *p *=* *0.031; Fig. 5*C*). After the period of HF suppression, no significant difference was identified (wakefulness: 0.69 ± 0.03, ketamine: 0.59 ± 0.05; mean difference = −0.11 [95% CI: −0.22, −0.03]; Wilcoxon S-R test, *p *=* *0.094). The absence of statistical difference in the θ-α frequency range was mainly explained by the connectivity in the θ band (5–7 Hz; Extended Data Fig. 5-2). Besides, we did not find statistical difference between wakefulness and ketamine conditions also in the LF range (1–4 Hz; Extended Data Fig. 5-2). Coherently, CD after the period of HF suppression was higher with ketamine than with both propofol and sevoflurane, while no difference was detected between these latter conditions (Mann–Whitney test, ketamine vs propofol, *p *=* *0.001; ketamine vs sevoflurane, *p *=* *0.004; Wilcoxon S-R test, propofol vs sevoflurane, *p *=* *0.375). We then identified a highly significant positive correlation between mean CD_(0.18–0.3 s)_ across channels and PCI*^ST^*_(0.08–0.6 s)_, thus revealing a connection between functional connectivity and perturbational complexity (linear fit, *p *=* *5.197 × 10^−10^, *R*^2^ = 0.683; Fig. 5*D*).

10.1523/ENEURO.0343-20.2021.f5-1 Extended Data Figure 5-1 Functional connectivity after cortical perturbation during sevoflurane anesthesia. ***A***, Example of epidural EEG activity in response to electrical stimulation (1 ms, 50 μA; dashed lines) from the same rat during wakefulness and during sevoflurane anesthesia (same rat of Fig. 5 to allow comparisons). The electrophysiological traces represent the superimposition of ensemble averages of ERPs from all electrodes. One channel is highlighted in bold for better illustrating difference in complexity. Below each butterfly plot, the spectrogram of mean relative HF power (20–40 Hz) is shown for each channel. The bottom part of each inner panel shows the increments in cortical connectivity compared to baseline for each condition in two time windows: during HF suppression, 0.08–0.18 s (left) and post-HF suppression, 0.18–0.3 s (right; rectangles indicate the two time windows). For each window, the connectivity matrix based on ISPC (5–14 Hz) is reported on the left and the topographical distribution (rostro-caudal orientation, R-C) of the CD for each channel is interpolated and shown on the right. ***B***, Mean CD across channels in the HF suppression period (left) and post-HF suppression (right) during wakefulness and sevoflurane anesthesia (*n* = 9 rats). During wakefulness CD was significantly higher than what observed during sevoflurane anesthesia in both time windows (HF suppression period, wakefulness: 0.71 ± 0.05, sevoflurane: 0.35 ± 0.05, mean difference = –0.37 [95% CI: –0.46, –0.23]; Wilcoxon S-R test, *p *=* *0.008; post-HF suppression, wakefulness: 0.60 ± 0.06, sevoflurane: 0.27 ± 0.04; mean difference = –0.33 [95% CI: –0.45, –0.21]; Wilcoxon S-R test, *p *=* *0.004). The floating axis on the right of each group comparison shows the mean difference between conditions. The effect size is reported as bootstrap resampling distribution of mean difference, with 95% CI represented by the bold black vertical line. Download Figure 5-1, TIF file.

10.1523/ENEURO.0343-20.2021.f5-2 Extended Data Figure 5-2 Functional connectivity across conditions for each frequency band, ***A–C***, Mean CD across channels following the period of HF suppression (0.18–0.3 s), from rats during wakefulness and ketamine anesthesia (***A***, *n* = 7), wakefulness and propofol anesthesia (***B***, *n* = 9), and wakefulness and sevoflurane anesthesia (***C***, *n* = 9). All comparisons are reported for each frequency band of interest (columns; from 1 to 40 Hz). During wakefulness, CD was significantly higher than what was observed during all anesthesia conditions, for each frequency band, with the exceptions of δ (1–4 Hz) and θ (5–7 Hz) bands with ketamine, for which no significant difference was identified. The mean difference between conditions is reported for each frequency band, below each group comparison. The effect size is reported as bootstrap resampling distribution of mean difference, with 95% CI represented by the bold black vertical line. Wilcoxon S-R test was adopted to assess statistical significance, which is reported in the figure as follows: *p <* *0.05, **p <* *0.01, ***p <* *0.001, *p *≥* *0.05 ns (not significant). Download Figure 5-2, TIF file.

Not only the overall amount of connectivity differed between conditions; averaging CD_(0.18–0.3 s)_ across channels organized in cortical regions (frontal: M2, M1; parietal: S1, PA, RS; occipital: V2, V1), revealed an uneven spatial distribution of CD during both wakefulness and ketamine anesthesia (wakefulness, 11 rats, Friedman test, *p *=* *0.019; ketamine, 7 rats, Friedman test, *p *=* *0.018; Fig. 5*E*), indicating a peak of connectivity in the occipital region. For each region, CD was similar between wakefulness and ketamine anesthesia (Mann–Whitney test, frontal, *p *=* *0.717; parietal, *p *=* *0.526; occipital, *p *=* *0.856) and no relation with cortical areas was detected with both sevoflurane and propofol (nine rats, sevoflurane, Friedman test, *p *=* *0.368; propofol, Friedman test, *p *=* *0.459). This suggested a similar degree of diversity in the evoked response among cortical areas during both wakefulness and ketamine conditions, which collapsed with sevoflurane and propofol.

**Figure 6.**
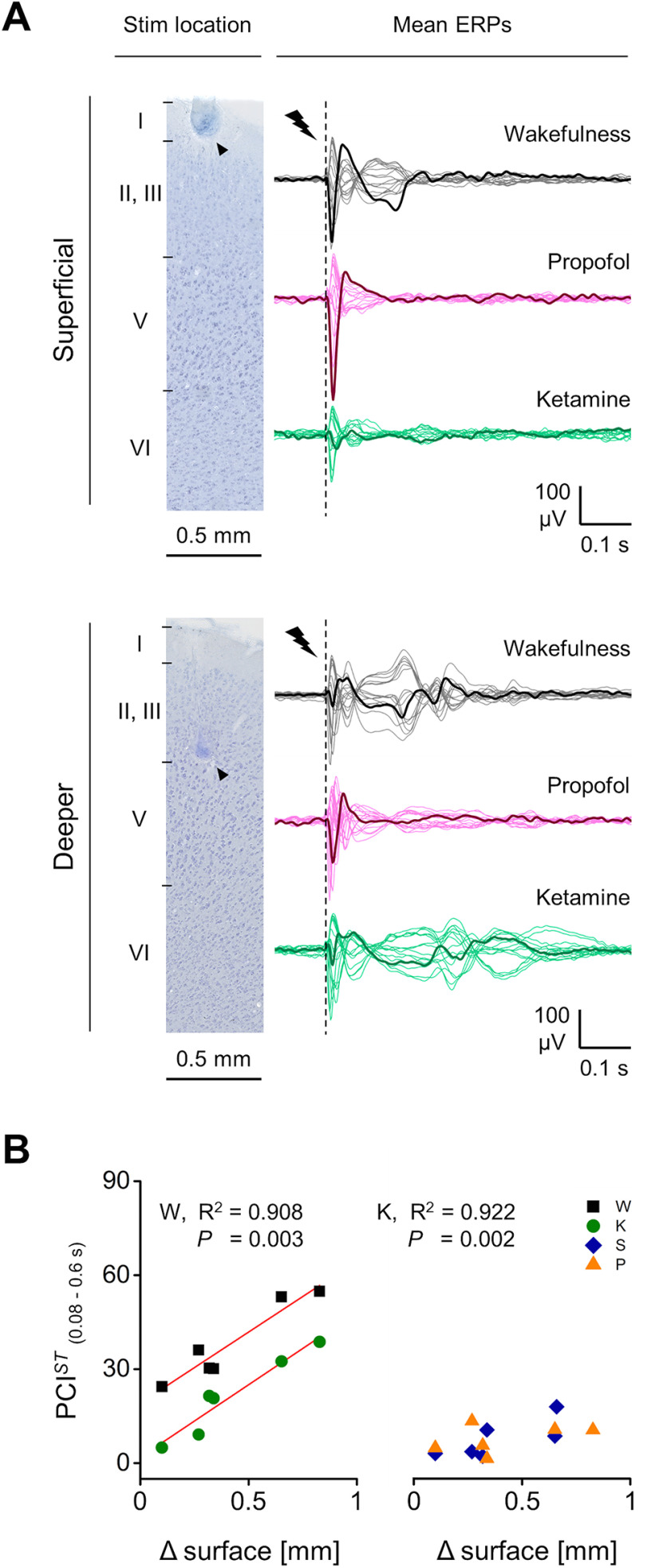
PCI*^ST^* positively correlated with the depth of the stimulation site within the M2 cortex during wakefulness and ketamine anesthesia, but not with propofol or sevoflurane. ***A***, left, Coronal cortical sections (Nissl staining) showing the location of the electrode for electrical stimulation in the right M2 cortex from one rat with the tip of the electrode positioned close to the cortical surface (top panel, superficial) and from another animal with the tip of the electrode deeper implanted in the cortex (bottom panel: deeper). Black arrowheads indicate the marks of one pole of the stimulating electrode. Right, Superimposition of mean ERPs from all recording electrodes in response to single pulse stimulation (1 ms, 50 μA; dashed line) from the same two rats shown on the left, during wakefulness (W) and propofol (P) and ketamine (K) anesthesia. One averaged ERP from the same channel (S1) is shown in bold to highlight differences across conditions. ***B***, Values of PCI*^ST^* (in time range: 0.08–0.6 s) from six rats and for all conditions are plotted against the corresponding distances of the stimulating electrode tips from the cortical surface and linearly fitted (coefficient of determination *R*^2^ and *p* value are reported if *p *<* *0.05). Strong positive correlations were identified for wakefulness and ketamine conditions with similar slopes (45.35 and 46.30, respectively), but not for propofol and sevoflurane (S) anesthesia. See Extended Data Figure 6-1 for in depth examination of correlation of PCI*^ST^* with stimulus location in wakefulness (examples of ERPs with sevoflurane are also shown). See Extended Data Figure 6-2 for correlations with stimulus location of number of principal components and state transitions, HF power and ITPC drop time, in all conditions. See Extended Data Figure 6-3 for in depth analysis across conditions, with only those rats with confirmed colocalization of stimulating electrodes in Layer II/III.

A complementary way to conceive the diversity in activity patterns induced by a stimulus is in relation to the specific site of stimulation. We took advantage of the variability, across rats, in the precise location of the stimulating electrode within M2, and tested for possible correlations with PCI*^ST^*. We did not find correlation with the position of the stimulating electrode along the rostro-caudal axis (in range: 3–5 mm from bregma; Extended Data Fig. 6-1), but we detected a strong and positive correlation of PCI*^ST^* with the location along the dorso-ventral axis (range: 0.1–0.83 mm from cortical surface, from Layers I to upper Layer V, mainly within Layer II/III; Extended Data Fig. 6-1). Specifically, during wakefulness PCI*^ST^*_(0.08–0.6 s)_ positively correlated with the depth of the stimulation (linear fit, *p *=* *0.003, *R*^2^ = 0.908; Fig. 6*A*,*B*) among six rats and a similar correlation was detected with ketamine (linear fit, *p *=* *0.002, *R*^2^ = 0.922). This was explained by the increasing number of both principal components and state transitions with stimulation depth, in both wakefulness and ketamine conditions (Extended Data Fig. 6-2). By contrast, we did not find any significant correlation between PCI*^ST^*_(0.08–0.6 s)_ and stimulation depth during both propofol and sevoflurane anesthesia (linear fit, propofol: *p *=* *0.387, sevoflurane: *p *=* *0.082; Fig. 6*A*,*B*). Finally, we repeated the main analysis within only the group of rats that had the stimulating electrodes located within the same cortical Layer II/III (Extended Data Fig. 6-3). Also, within this subgroup of five animals, we found the same relations across conditions as shown for the group of all animals (Extended Data Fig. 6-3). This result indicates that the observed dependency between stimulation depth and PCI*^ST^* did not affect the comparisons between wakefulness and anesthesia of our experiments.

10.1523/ENEURO.0343-20.2021.f6-1 Extended Data Figure 6-1 Correlation of PCI*^ST^* with the stimulus location in M2 during wakefulness. ***A***, The same representation of coronal sections of rat brain with the actual position of chronically implanted bipolar electrodes in right M2 has already been shown in Extended Data Figure 1-3 and is reported here for clarity. Dots represent the two poles of the bipolar electrodes chronically implanted in eight rats (for each section, bipolar electrodes from different rats are color coded). Stimulating electrodes covered a cortical area from ∼5 to ∼3 mm with respect to bregma in rostro-caudal direction (R-C, *y*-axis) and a depth range from ∼0.1 to ∼0.8 mm calculated from cortical surface (*z*-axis), mainly corresponding to Layer II/III. In one rat the electrode was found to be placed in Layer I, while in another animal it was positioned at the edge between Layers III and V. ***B***, ***C***, PCI*^ST^* from time range 0–0.6 s (up) and from time window 0.08–0.6 s (bottom) are plotted for each rat (*n* = 7) and recording session during wakefulness (one to three recordings for each rat) against the position of the stimulating electrode along the *y*-axis (***B***) and along the *z*-axis (***C***). Possible correlations with the position of stimulating electrode have been tested and no significant relation was detected between PCI*^ST^* and the position of stimulating electrodes along the *y*-axes (***B***; up, PCI*^ST^*_(0–0.6 s)_, linear fit, *p *=* *0.155, *R*^2^ = 0.122; bottom, PCI*^ST^*_(0.08–0.6 s)_, linear fit, *p *=* *0.129, *R*^2^ = 0.138). Otherwise, a highly significant and strong positive correlation between PCI*^ST^* (in both time windows) and the depth of the stimulation site have been found (***C***; up, PCI*^ST^*_(0–0.6 s)_, linear fit, *p *=* *9.329 × 10^−4^, *R*^2^ = 0.506; bottom, PCI*^ST^*_(0.08–0.6 s)_, linear fit, *p *=* *2.821 × 10^−5^, *R*^2^ = 0.676). The coefficient of determination *R*^2^ and the *p* value are reported. ***D***, Example of evoked activity from one rat with stimulating electrode implanted close to cortical surface (left) and from other animals with stimulating electrode placed deeper in M2 (right), during wakefulness (up) and sevoflurane anesthesia (bottom). The electrophysiological traces represent the superimposition of mean ERPs from all recording channels. One mean ERP from the same channel (S1) is in bold in each condition to better highlight differences in complexity. Data are from the same animals reported in Figure 6. Download Figure 6-1, TIF file.

10.1523/ENEURO.0343-20.2021.f6-2 Extended Data Figure 6-2 Dependency of ERP features on stimulation depth. We tried to clarify the dependency of PCI*^ST^*on stimulation depth by analyzing the number of principal components (NPC) and NST, since PCI^ST^ is equivalent to the product of NPC and NST, as well as HF power and ITPC drop time in relation to stimulus position in the same six rats of Figure 6, during wakefulness (W), propofol (P), sevoflurane (S), and ketamine (K) conditions. As expected, both the NPC (***A***) and NST (***B***) increased with the stimulation depth in both wakefulness and ketamine conditions (with different slopes). The NPC increased also with sevoflurane as a function of stimulus depth, but this increment did not translate into higher PCI*^ST^* (see Fig. 6). ***C***, The averaged HF power (in range 20–40 Hz, 0.08–0.18 s) became more negative by increasing the stimulation depth in both ketamine and propofol condition. ***D***, The ITPC drop time occurred later with deeper stimulations only with ketamine. Measurements from 6 rats and for all conditions are plotted against the corresponding distances of the stimulating electrode tips from the cortical surface and linearly fitted (fitting function, coefficient of determination *R*^2^ and *p* value are reported if *p *<* *0.05). Download Figure 6-2, TIF file.

10.1523/ENEURO.0343-20.2021.f6-3 Extended Data Figure 6-3 Comparison of ERPs between wakefulness and anesthesia for rats with colocalized stimulating electrode in Layer II/III. ***A–F***, Only rats with confirmed colocalization of stimulating electrodes in Layer II/III were considered for this analysis (*n* = 5 rats). Because of the reduced total amount of animals, to increase the statistical power, data from different recording sessions from the same rats were grouped together in wakefulness condition (W), generating a sampling size of 13 observations (four rats with data from three recordings each, plus one rat with data from one recording). With the same purpose, data from general anesthesia produced by GABAergic drugs (sevoflurane and propofol, P&S) were grouped together, generating a sample size of nine observations (four rats with data from both anesthetic conditions, plus one rat with data only from sevoflurane condition). Data from four rats, each with one observation, belonged to ketamine condition (K). Several features of the evoked response were compared across conditions at the level of observations. ***A***, PCI*^ST^* (in time range 0.08–0.6 s) decreased from wakefulness to all other conditions; however, with ketamine, it was higher than in the propofol and sevoflurane group. ***B***, This was explained by a reduced number of principal components from W to P&S, while no significant difference was detected between W and K. ***C***, Conversely, the NST was reduced from wakefulness to both P&S and K conditions. ***D***, In P&S and K conditions, the mean HF power (in range 20–40 Hz, 0.08–0.18 s) was always below 0 dB, indicating a suppression period. On the contrary, it was always above 0 dB, thus significantly higher during wakefulness. ***E***, The averaged ITPC drop time (in range 8–40 Hz) during wakefulness occurred significantly later than in propofol and sevoflurane anesthesia, while no significant difference was found in comparison to ketamine condition. ***F***, Similarly, the mean CD (in range 5–14 Hz, 0.18–0.3 s) dropped from W to P&S, while no significant difference was found between W and K conditions. The mean differences between P&S and W and between K and W groups are reported for each measure, below each group comparison. The effect size is reported as bootstrap resampling distribution of mean difference, with 95% CI represented by the bold black vertical line. Mann–Whitney test was adopted to assess statistical significance, which is reported in panels as follows: *p <* *0.05, **p <* *0.01, ***p <* *0.001, *p *≥* *0.05 ns (not significant). Download Figure 6-3, TIF file.

## Discussion

In this study, we established a method for reliable, chronic recording of multichannel, epidural EEG in response to local, cortical, electrical stimulation in rats (Fig. 1; Extended Data Figs. 1-1, 1-2, 1-3). We then successfully used this method to quantify the PCI (Casali et al., 2013). PCI is a proposed measure of the brain’s capacity to sustain complex dynamics and conscious brain states (Casali et al., 2013) and here, a new variant of this measure (PCI*^ST^*) has been adopted, since it is more suitable for intracranial electrical stimulation (Comolatti et al., 2019). Both PCI and PCI*^ST^* were extensively validated on humans as reliable consciousness metrics (Casali et al., 2013; Sarasso et al., 2015; Casarotto et al., 2016; Rosanova et al., 2018; Comolatti et al., 2019), and here we directly applied a PCI measure for the first time in a non-human animal species *in vivo*, by using the same parameters adopted for humans (Comolatti et al., 2019), thus allowing direct interspecies comparison and validation.

For measuring PCI*^ST^*, we stimulated the M2 cortex because it is a highly integrated area in rodent neocortex (Zingg et al., 2014; Barthas and Kwan, 2017), suitable for triggering widespread and differentiated responses, and it resembles human premotor cortex, often used for probing PCI (Casali et al., 2013; Sarasso et al., 2015). Within M2, we targeted Layer II/III because it contains mainly cell bodies of pyramidal neurons that project to other ipsilateral and contralateral cortical areas (Petreanu et al., 2007; Harris and Shepherd, 2015; Gerfen et al., 2018), and thus seemed suitable for investigating corticocortical integration. We tested complexity during wakefulness and general anesthesia induced by propofol, sevoflurane, and ketamine, similarly to a previous study in humans (Sarasso et al., 2015), thus allowing a direct comparison across species. For each anesthetic drug, the depth of anesthesia was set at the minimal dose that abolished all motor responses to pain stimulation, and spontaneous movements were also not seen. At the same level of behavioral unresponsiveness, propofol and sevoflurane induced a spontaneous EEG activity characterized by high amplitude, LF waves, while ketamine anesthesia yielded sustained, fast, desynchronized EEG activity, resembling the awake state. In agreement with previous studies in humans (Colombo et al., 2019; Lendner et al., 2019), the spectral exponent of the relative periodogram (20–40 Hz) was reduced by propofol and sevoflurane, but with ketamine it remained similar to wakefulness (Figs. 1, 4; Extended Data Fig. 4-1). During wakefulness, the electrical stimulation produced a long-lasting and complex ERP with a sequence of reproducible voltage inversions in time and across cortical areas. The ERP had a peak power in the 8- to 25-Hz range, but reached up to 40 Hz and remained phase-locked for ∼0.3 s. Conversely, in propofol anesthesia, the ERP was short-lasting, with few polarity changes, and after a brief HF response, ITPC dropped within ∼0.1 s (Figs. 1, 2). Thus, PCI*^ST^* was reduced from wakefulness to propofol anesthesia in all rats (Fig. 1; Extended Data Fig. 3-2), in agreement with previous results from humans (Sarasso et al., 2015).

Next, we investigated possible mechanisms underlying the reduced complexity. It has been hypothesized that cortical bistability may be important for preventing complex cortical dynamics (Tononi et al., 2016; Sanchez-Vives et al., 2017; Storm et al., 2017). Specifically, when the arousing input from the brainstem and thalamus is reduced or counteracted, such as during NREM sleep, general anesthesia, or deafferentation, cortical circuits tend to become bi-stable and fall into synchronized down-states (neuronal hyperpolarization) following previous activations, or up-states (Steriade et al., 1993, 2001; Sanchez-Vives and McCormick, 2000; Timofeev et al., 2000; Volgushev et al., 2006). At the network level, these changes give rise to slow wave oscillations in the spontaneous EEG observed during NREM sleep and general anesthesia, where ON-periods of HF activity and firing alternate with OFF-periods of HF suppression and neuronal silence (Steriade et al., 1993, 1996, 2001; Mukovski et al., 2007; Vyazovskiy et al., 2009a,b). Neuronal down-states are thought to be generated mainly by activity-dependent K^+^ currents (Timofeev et al., 2001; Compte et al., 2003) and/or synaptic fatigue and inhibition (Esser et al., 2007; Funk et al., 2017), and it has been suggested that the same mechanisms may be triggered by the initial response to cortical stimulation (Tononi et al., 2016; Sanchez-Vives et al., 2017). The “induced down-state” would then interrupt the deterministic and long-lasting sequence of complex neuronal interactions that are thought to give high PCI (Tononi et al., 2016; Sanchez-Vives et al., 2017). Indirect evidence for this mechanism was recently observed in humans during NREM sleep and UWS, when an OFF period characterized by HF suppression in the EEG was found to follow the initial response to cortical stimulation, and to correlate with the interruption of the phase-locked activation (Pigorini et al., 2015; Rosanova et al., 2018). Similar results were obtained in cortical slices *in vitro,* with pharmacological reduction of bistability (D’Andola et al., 2018). In agreement with these results, we found that propofol anesthesia induced a profound and widespread HF suppression, after the initial response, ∼0.08 s after the stimulation, in all animals, suggesting the presence of an OFF period (Fig. 2; Extended Data Fig. 2-3).

Previous experiments in rats showed that during NREM sleep, OFF periods of transient and synchronized neuronal silence coincided with spontaneous slow waves in the EEG (Vyazovskiy et al., 2009b). Moreover, it has been shown that electrical, cortical stimulation in rats during NREM sleep evoked slow waves that were highly similar to the spontaneous slow activity (Vyazovskiy et al., 2009a). Coherently, the electrical stimulation during propofol anesthesia induced an ERP characterized by a phase-locked, SC (<4 Hz), with a higher amplitude that was closely related to the amplitude of the ongoing spontaneous slow oscillation, and which, on average, coincided in time with the end phase of the HF suppression (Extended Data Fig. 2-1).

Importantly, by increasing the stimulus intensity during propofol anesthesia, we were always able to obtain a similar increased cortical excitation as during wakefulness, but the putative OFF period was never abolished. Instead, both the magnitude and duration of the HF suppression increased linearly with the stimulus intensity (Fig. 3; Extended Data Fig. 2-2), suggesting that the OFF period likely reflected an adaptation-like induced down-state. This is in line with the idea that when bistability dominates, a stronger activation leads to a more hyperpolarized state, and is compatible with results from slow waves in cortical slices, where an increased firing rate during the up-state was followed by longer and more hyperpolarized down-state (Sanchez-Vives et al., 2010). The same mechanism might underlie previous findings in rats, showing that ON periods with higher neuronal firing rate were followed by longer OFF periods with slow waves of higher amplitude, during early NREM sleep (Vyazovskiy et al., 2009b). Consistently with these results, by increasing stimulus intensity, we found a highly significant correlation between the maximal amplitude of the slow ERP component and the magnitude of the HF suppression (Fig. 3). Importantly, also the latency of maximal HF suppression and the time of the ITPC drop were positively correlated (Fig. 3; Extended Data Fig. 3-1), supporting the idea that the OFF period can indeed interrupt the deterministic response to the stimulation, as previously observed in UWS and NREM sleep in humans (Pigorini et al., 2015; Rosanova et al., 2018). Interestingly, we found that the ITPC drop was better temporally related to the end phase of the HF suppression rather than to the time of deepest HF suppression (Extended Data Fig. 3-1) as suggested in previous works (Pigorini et al., 2015; Rosanova et al., 2018). This could be explained either by a temporal imprecision of the wavelet convolution, or by different timing of the termination of firing of the involved neurons within the OFF period. However, also the averaged peak latency of the slow ERP component was found to coincide in time with the end phase of the HF suppression period (Extended Data Fig. 2-1), thus suggesting a common physiological mechanism.

With propofol, the HF response was not simply abolished after the OFF period: stimulation-induced HF activations were still detectable later, even if they were sparse in space and time, and not phase-locked, more resembling a modulation of ongoing activity (David et al., 2006). In contrast, during wakefulness, later HF activations were still phase-locked, sustained and widespread, indicating strong deterministic responses (Figs. 2, 4). However, traces of HF suppression were detected also during wakefulness in response to stimulation, but these never interrupted long-lasting, phase-locked activations. Compared with propofol anesthesia, the HF suppression during wakefulness was shallower, briefer and insensitive to variations in stimulus intensity (Extended Data Fig. 2-3), suggesting a different mechanistic origin. A similar effect during wakefulness was also previously observed in humans, from electrodes close to the stimulation site (Borchers et al., 2011; Pigorini et al., 2015), resembling a momentarily increased recruitment of inhibition caused by vigorous electrical stimulation (Borchers et al., 2011; Pigorini et al., 2015). This is also in line with previous experiments involving electrical stimulation of the cortex of awake rats (Vyazovskiy et al., 2013): a first brief activation in response to stimulation was followed by increased firing of putative inhibitory interneurons, which preceded a period of synchronized neuronal silence (Vyazovskiy et al., 2013). However, in the same experiments, the OFF period was more long-lasting and synchronous after longer periods of continuous wakefulness, thus showing a modulatory effect of sleep pressure on neuronal responsiveness (Vyazovskiy et al., 2013).

It therefore seems important in the future to compare EEG signatures of bistability and PCI*^ST^* also between more graded brain states or sub-states within wakefulness and NREM sleep. In light of our and previous results (Vyazovskiy et al., 2013), one would expect that the tendency of cortical circuits to fall into an OFF period after a brief activation should increase, and PCI*^ST^* should decrease, as a function of the time spent in wakefulness, with the maximal neuronal bistability and minimal PCI*^ST^* during the early stage of NREM sleep (Vyazovskiy et al., 2009a,b; Pigorini et al., 2015; Casali et al., 2013). This would be consistent with the local sleep events, with localized OFF periods and EEG slow waves, that were found to spontaneously occur in rats during long periods of sleep deprivation (Vyazovskiy et al., 2011). Similarly, one may expect that a gradually reduced level of general anesthesia will reduce the magnitude and duration of the evoked OFF period, with a gradual restoration of PCI*^ST^*. Indeed, Mégevand et al. (2008) found that lighter compared with deeper anesthesia induced a higher number of components in the EEG response to sensory stimulation in mice, with an increased propagation of electrical activation, and recently, a variant of perturbational complexity adapted from an *in vitro* study was found to increase with reduced anesthesia in mice (Dasilva et al., 2021). Also, within the same level of sleep pressure during wakefulness, neuronal responsiveness and cortical complexity might change substantially depending on the state of activity and arousal. Indeed, it was shown in mice that moderate arousal or alert wakefulness was associated with a stable, hyperpolarized membrane potential in cortical neurons and a better behavioral performance, compared with quiet and active wakefulness (McGinley et al., 2015). Thus, a careful control of these parameters, in association with PCI*^ST^*, may provide important insights into the neuronal basis for integration of information in cortical circuits.

It is likely that a cortical network that can sustain the propagation of long-lasting, complex sequences of neuronal activations has a high and diversified level of connectivity (Massimini et al., 2009). To test this idea, we examined the functional connectivity following the stimulation within the frequency range that corresponded to the more long-lasting phase-locked response (θ-α, 5–14 Hz), which has also been implicated in long-range interactions across brain regions (Lisman and Jensen, 2013; Hyafil et al., 2015). Available evidence from mice (Tamura et al., 2017) and monkeys (Liebe et al., 2012) support the role of θ-α oscillations in organizing HF activity and coupling occipital-frontal regions during wakefulness and cognitive tasks. Thus, the θ-α range seemed well suited for testing the long-range functional network connectivity that is particularly relevant for PCI. We used ISPC as a measure of the consistency of phase-based connectivity across trials, to capture the spatial representation of the deterministic sequence of neuronal events that generates the complexity of the response in time. Consistently, we found a strong positive correlation between the derived CD and PCI*^ST^* (Fig. 5). Indeed, the response during wakefulness showed a high degree of connectivity with a peak in occipital areas, highlighting a certain diversity in the evoked neuronal activations. In contrast, propofol anesthesia reduced cortical connectivity, and the spatial distribution of the remaining functional connections became more uniform (Fig. 5; Juel et al., 2018), resembling a reduction of both integration and differentiation of the neuronal response. Even if we cannot exclude some persisting information flow between distant neurons, which can be even enhanced during NREM sleep in rats after a behavioral task (Olcese et al., 2018), our finding fits with the known effect of propofol in suppressing long-range spontaneous network interactions (Lewis et al., 2012). Indeed, we found a reduced connectivity for each of the frequency bands examined (from 1 to 40 Hz; Extended Data Fig. 5-2). Our results are also consistent with the reduced variability in the complexity of evoked responses to stimulations at different cortical depths, observed here (Fig. 6). PCI*^ST^* was indeed found to linearly increase with the cortical depth of stimulation during wakefulness, but not with propofol, within the tested range across rats (from Layer I to upper part of Layer V, with most positions within Layer II/III; Fig. 6; Extended Data Fig. 6-1). This linear relation has to our knowledge not been reported before, presumably because most PCI experiments were done in humans with TMS, and indicates a conserved differentiation during wakefulness that fades with propofol anesthesia.

We next used the same approach during sevoflurane anesthesia. Sevoflurane and propofol are known to share some molecular mechanisms, such as enhancement of GABA_A_ receptors (Bieda and MacIver, 2004; Sebel et al., 2006) and inhibition of voltage-gated Na^+^ channels (Ouyang et al., 2003, 2009), but there are also important differences. Thus, sevoflurane opens TASK two-pore-domain (2P) K^+^ channels, unlike propofol (Putzke et al., 2007), and differently affects sensory processing (Arena et al., 2017). Nevertheless, sevoflurane produced effects that resembled those with propofol. Soon after the initial response to stimulation, a period of deep HF suppression occurred, preceding an early drop of the phase-locked response. Later on, only sparse and not phase-locked HF activations were detected and both functional connectivity and diversity were reduced compared with wakefulness, leading to low PCI*^ST^* (Extended Data Figs. 4-1, 5-1, 5-2; Figs. 5, 6). Taken together, these results support the hypothesis that the down-state may serve as a general mechanism for interrupting complex cortical dynamics. This mechanism seems indeed to be shared across anesthetic drugs, animal species, and brain states, including NREM sleep (Pigorini et al., 2015) and UWS (Rosanova et al., 2018) in humans.

Nevertheless, our results with ketamine may seem to challenge this idea. Ketamine anesthesia is known to produce a state of behavioral unresponsiveness with “dream-like,” vivid experiences (Collier, 1972). A previous study in humans found no significant difference in PCI between wakefulness and ketamine anesthesia (Sarasso et al., 2015), but signs of bistability were not examined in this condition (Sarasso et al., 2015). Here, we found that ERPs during ketamine showed mixed features: the initial brief response was rapidly followed (∼0.08 s later) by an OFF period, resembling propofol anesthesia, but this did not prevent long-lasting deterministic activations in nearly half the animals, and the duration of the resulting phase-locked response was close to that of wakefulness (Fig. 4). After the OFF period, a later increase in HF activity was highly frequent, but neither consistently phase-locked and deterministic, like in wakefulness, nor consistently not phase-locked, like with propofol and sevoflurane (Fig. 4). During ketamine anesthesia, the time course of PCI*^ST^* revealed similarities to wakefulness, but resulting in an overall reduction of complexity. However, PCI*^ST^*from the onset of the OFF period was significantly higher than in propofol/sevoflurane anesthesia, thus indicating an intermediate level of perturbational complexity with ketamine, regardless of the similar state of behavioral unresponsiveness (Fig. 4). Consistently, the functional connectivity across cortical regions was reduced compared with wakefulness during the OFF period, but recovered soon afterward in the θ frequency range (5–7 Hz; Extended Data Fig. 5-2) and a wakefulness-like pattern of CD across space was maintained, suggesting conserved integration and diversity (Fig. 5). Another important sign of a conserved differentiation during ketamine anesthesia was the positive linear relation between PCI*^ST^* and the depth of the site of stimulation within M2, which was also observed in wakefulness, with similar slopes (Fig. 6). The mixed results with ketamine may suggest some variability or unstable effects of this drug. In support of this idea, a recent study in humans found that the EEG activity spontaneously fluctuated between states of low and high complexity during ketamine anesthesia, until awakening (Li and Mashour, 2019). Certainly, since our experimental design was adopted to reproduce experiments in humans, we could only indirectly infer signs of down-states by detecting OFF periods of HF suppression in EEG. Indeed, HF suppression is a more direct indication of reduced neuronal activation relative to ongoing activity, and does not necessarily indicate neuronal hyperpolarization, which can be observed only by intracellular recordings. For example, a coordinated reduction in presynaptic release might also produce an OFF period in the EEG, if compared with the enhanced spontaneous activity that is typically induced by ketamine (Brown et al., 2010; Ferro et al., 2017), without implying hyperpolarization. Although our data cannot yet resolve this issue, they illustrate the need for intracellular recordings to determine the role of bistability in the interruption of complex dynamics. Moreover, the novel correlation that we found between PCI*^ST^* and the cortical depth of stimulation represents a promising starting point for studying the roles of specific cortical layers and micro circuitries in sustaining cortical complexity.

In conclusion, we demonstrated that rodent cortical circuits can respond to focal stimulation with long-lasting sequences of deterministic, complex interactions during wakefulness, which are disrupted during general anesthesia, consistently with previous results in humans (Sarasso et al., 2015). In agreement with previous studies (Pigorini et al., 2015; D’Andola et al., 2018; Rosanova et al., 2018), we provided indirect evidence for a connection between cortical bistability, interruption of deterministic responses, and disruption of cortical connectivity and complexity. Our results improve our understanding of the cortical dynamics that in humans have been associated with consciousness, and our method opens a range of future possibilities for more detailed, mechanistic investigations of brain states and their transitions.

## Synthesis

Reviewing Editor: Rae Silver, Columbia University Decisions are customarily a result of the Reviewing Editor and the peer reviewers coming together and discussing their recommendations until a consensus is reached. When revisions are invited, a fact-based synthesis statement explaining their decision and outlining what is needed to prepare a revision will be listed below. The following reviewer(s) agreed to reveal their identity: NONE.

Major comments:

The authors assess the spatiotemporal complexity of cortical responses to local stimulation in rats by using PCIst, a recently established measure of cortical integration and deafferentation developed in humans. As a first step to the use of PCIst in animal models, they apply this measure in waking and 3 anesthesia conditions, and obtain results broadly consistent with the findings in humans. The paper is well written and these results are important as a first validation of the PCI approach to infer the level of consciousness by measuring the complexity of cortical dynamics. It is unfortunate, however, that the scope of the current study is quite limited and several crucial experiments and analyses were not done, starting from the obvious comparison between waking and sleep, as well as between light and deep anesthesia. If these aspects can be aded, the paper would be much strengthened. If not, the consideration of these parameters can be drawn from the literature or mentioned as future directions.

An innovative aspect of the work is the examination of how stimulation depth is reflected in PCI. However, this aspect was minimally addressed in the Discussion. The discussion of related studies in animals should be amplified. Several studies in rodents, in fact, assessed the effect of electrical stimulation across wakefulness and sleep, and applied complexity and connectivity measures to neural and neuronal activity patterns. Examples are Vyazosvkiy at al. 2013, Abasolo et al. 2015, Olcese et. al 2018 (to cite only a few). The authors should expand this section of the discussion (or, better, introduce such a section) to allow a proper comparison with the field.

Additional comments:

As detailed below, there are several points that should be clarified.

1-It seems that anesthesia levels were selected with the goal of “....maintaining loss of righting reflex in 100 % of the tested rats” (page 8). But if the animals were confined inside a plastic tube, how could the loss of the righting reflex be measured? This brings another issue, which is the lack of PCIst measurements under lighter anesthesia, when presumably also cortical bistability was absent. In general, a careful analysis of the behavior of the animal while the stimuli were delivered seems to be missing.

2-(Page 13) “The baseline and response window were defined as (from -0.5 to -0.005 s) and (from 0 to 0.6 s)”. Please clarify why these specific time windows were chosen. Same for Figure 1 F: why does the “response” parameter of PCIst extend to 0.6? Is 0.6 when the temporal progression of PCIst becomes the same between the two conditions (W; P) ...? If so, how was this established? Figure 1F should extend the x axis to the point where W and P meet or to at least until the “response” parameter (0.6). Is this the way Comollatti et al. 2019 defined the “response” parameter for PCIst? It seems that in previous studies different parameters were used for hEEG and intracranial electrical stimulation.

3- Figure 1 C vs 2A: Please clarify why the HF suppression during propofol does not seem to coincide with a slow component of the ERP or the 5 individual traces that are shown. Is this specific of propofol? It would be nice to also see more individual traces shown as supplementary material. The background activity at the time of stimulus delivery is probably important, and the ERP may hide this.

4-Page 19; it is stated that ketamine was used “at a dose that abolished all motor responses to painful stimuli (∼1.8 mg/kg/min i.v; Fig. 4A)”. Does this mean that the animal was immobile at all times when the electrical stimuli were given? The anesthesia may abolish the response to pain stimuli, but spontaneous movements may still happen and make the PCI measure variable.

5- Page 21 “Sevoflurane anesthesia in 10 rats produced results that were indistinguishable from what we observed with propofol (Extended Fig. 1-4 and Extended Fig. 4-1)”. This statement is quite misleading and should be qualified: PCIst values may be similar, but the ERPs are not.

6- Page 21: Why was the ISPC averaged between 5 and 14 Hz and not between other frequencies? The authors state that these frequencies “are implicated in long-range and feedback interactions across visual areas.” But the two papers that they cite are from humans and NHPs. If this translates to the rat, which I am concerned about, they should at least cite rodent studies here.

7- I wonder, in figure 3D, why they correlate ITPC drop with the end of the putative OFF period, rather than the start. Shouldn’t it be the start of the OFF period that causes this drop? Their figures seem to suggest so. I guess it is because previous studies have correlated ITPC drop with the end of the OFF period?

8- The authors should avoid calling their periods of HF suppression “OFF periods” without MUA, though I have no problem if they want to point out the relationship between the two phenomena in the discussion.

9- Perhaps I missed it, but I could not find any discussion of why the authors targeted L2/3 rather than, say, L5. It should be clearly stated that “deeper” in this study still means superficial layers.

10-Page 25 “and it has been suggested that the same mechanisms may be triggered by the initial response to cortical stimulation.” Please provide the references.

## Author Response

POINT-BY-POINT REPLY TO THE REVIEWS

Editorial Suggestion

eNeuro is launching a new effort to improve rigor, transparency, and reproducibility, with a strong

encouragement to use estimation statistics. ... If the use of estimation statistics applies to your

research, you may consider including it for the revision. We will regularly highlight these papers to

the community to show how helpful estimation statistics can be, increasing the exposure of your work.

We would like to thank the Editor for this positive suggestion, and have now added

estimation statistics to the simple group comparisons in our manuscript. We used the website

https://www.estimationstats.com/#/ to create the estimation statistic plots, which were then cropped

and pasted in the original figures, showing the effect size. The 95% confidence intervals are reported

in the text, for the main figures, or in the figure captions, for the Extended Data Figures. We also

mentioned estimation statistics in the method section, page 15.

Manuscript Instructions

Extended Data Figures are not referenced in the legends of the figures they are supporting

We now added references to Extended Data Figures at the end of all captions of the main figures.

Synthesis of Reviews:

Significance Statement Comments for Author (Required): Appropriate

Comments on the Visual Abstract for Author (Required): NA

Synthesis Statement for Author:

Major comments:

1- The authors assess the spatiotemporal complexity of cortical responses to local stimulation in rats

by using PCIst, a recently established measure of cortical integration and deafferentation developed

in humans. As a first step to the use of PCIst in animal models, they apply this measure in waking and

3 anesthesia conditions, and obtain results broadly consistent with the findings in humans. The paper

is well written and these results are important as a first validation of the PCI approach to infer the

level of consciousness by measuring the complexity of cortical dynamics. It is unfortunate, however,

that the scope of the current study is quite limited and several crucial experiments and analyses were

not done, starting from the obvious comparison between waking and sleep, as well as between light

and deep anesthesia. If these aspects can be added, the paper would be much strengthened. If not, the

consideration of these parameters can be drawn from the literature or mentioned as future directions.

Thank you for the comments. As stated, this was a first attempt to directly apply the PCI

techniques from humans to rats. This has not been trivial, since many parameters in rodents differ 2

from humans, starting from the obvious brain dimension to the lack of standards for EEG recording

and electrical stimulations. However, we agree that a comparison between waking and sleep would be

highly relevant and interesting, and we are currently working towards this as well, as several new

experiments and controls are needed in order to obtain a better understanding of the neuronal

mechanisms behind cortical complexity and bistability. For example, we have started new

experiments that combine PCI with unit recordings and we plan to also include NREM and REM

sleep conditions, but we are not ready yet to present these data. As suggested by the Reviewer, we

have now expanded the discussion considerably with new considerations regarding sleep states, sleep

pressure, in addition to light and deep general anesthesia, on pages 27, 28, 29, 30.

2- An innovative aspect of the work is the examination of how stimulation depth is reflected in PCI.

However, this aspect was minimally addressed in the Discussion. The discussion of related studies in

animals should be amplified. Several studies in rodents, in fact, assessed the effect of electrical

stimulation across wakefulness and sleep, and applied complexity and connectivity measures to

neural and neuronal activity patterns. Examples are Vyazosvkiy at al. 2013, Abasolo et al. 2015,

Olcese et. al 2018 (to cite only a few). The authors should expand this section of the discussion (or,

better, introduce such a section) to allow a proper comparison with the field.

Thank you for your comment and also for the references to relevant articles. We agree that the

relation between PCI and the stimulation depth is a novel insight, but minimally addressed. Following

the suggestion by the Reviewer, we have now expanded this section, also proposing a new hypothesis

that can be tested in future experiments, on page 31. We also expanded the discussion comparing our

results with those from other studies in rodents involving both stimulation and recording of

spontaneous activity, including two of the suggested papers. However, we preferred to expand the

already existing sections instead of creating a new one, to maintain the original structure of the

discussion. Specifically, we have included references to spontaneous EEG and unit recordings during

NREM sleep (pages 27, 28), electrical stimulation during NREM sleep (page 27), electrical

stimulation during wakefulness (page 29), spontaneous activity during wakefulness and the influence

of sleep pressure (page 29), stimulation with different anesthetic doses (page 29), different levels of

arousal during wakefulness (page 30), functional connectivity (page 30), and possible specific roles of

layer 5 (page 31).

Additional comments:

As detailed below, there are several points that should be clarified.

1- It seems that anesthesia levels were selected with the goal of “....maintaining loss of righting

reflex in 100 % of the tested rats” (page 8). But if the animals were confined inside a plastic tube, how

could the loss of the righting reflex be measured? This brings another issue, which is the lack of PCIst

measurements under lighter anesthesia, when presumably also cortical bistability was absent. In

general, a careful analysis of the behavior of the animal while the stimuli were delivered seems to be

missing.

We are thankful for this comment. The Reviewer is right that a specific explanation of how

the starting doses of anesthesia were chosen was indeed missing. We have now added a description of 3

our in the Methods section, on page 7. Initial doses were chosen in agreement with the literature

(Idvall et al., 1980; Brammer et al., 1993; Benito et al., 2010; Arena et al. 2017). Next, the doses were

tested in a set of pilot experiments (5 rats), in order to determine which doses were needed to ensure

the loss of righting reflex, and this was achieved in all the pilot animals (n=5). In these experiments,

the rats were tested outside the plastic tube, laid on their back on a heating pad, at a flat surface inside

their cage, and without any physical restraint. We checked for the presence of any attempt to assume

the upright position within about 20 min. If any such movements were detected, the doses were

increased in a stepwise manner. For the remaining experiments, we used the highest dose needed to

ensure the loss of righting reflex among the tested animals. (See page 7).

It might be worth mentioning that only with ketamine anesthesia, we detect increased muscle

tone in some of the rats, soon after anesthesia induction, which was accompanied by small whisker

deflections. This muscular activity spontaneously disappeared within a few minutes of ketamine

infusion or by stepwise incrementing the administration rate.

It’s important to note that the test for loss of righting reflex was only a coarse procedure to

assess the starting dosage of anesthesia, at which reactions to pain stimuli were still present. The

experimental doses were more accurately determined for each single rat, as the minimal doses that

abolished any motor reaction to pain stimulation (tail pinched 3 consecutive times with a forcep), as

also described on page 7. The motor response to tail pinching consisted of a lateral, wide movement

of the tail and/or in altered respiratory rhythm, accompanied by a sudden chest movement that could

be seen through the transparent acrylic cylinder wall and because the tail was left outside. At the

experimental doses used here, we did not see any spontaneous movement, with any of the 3

anesthetic drugs.

We did not test very low doses of anesthesia mainly because in states close to full awakening,

rats can become paradoxically excited, with the consequent difficulty in maintaining them in the

recording setup. However, the suggestion regarding the reduced/absent bistability is certainly

interesting and can be tested in future experiments. We have now addressed the issue about low

anesthesia doses in the Discussion, on page 29, where we cite two papers that provided evidence in

support of reduced bistability and increased complexity at low compared to high anesthetic doses

(Mégevand et al., 2008; Dasilva et al., 2021).

2- (Page 13) “The baseline and response window were defined as (from -0.5 to -0.005 s) and

(from 0 to 0.6 s)”. Please clarify why these specific time windows were chosen. Same for Figure 1 F:

why does the “response” parameter of PCIst extend to 0.6? Is 0.6 when the temporal progression of

PCIst becomes the same between the two conditions (W; P) ...? If so, how was this established?

Figure 1F should extend the x axis to the point where W and P meet or to at least until the “response"

parameter (0.6). Is this the way Comollatti et al. 2019 defined the “response” parameter for PCIst? It

seems that in previous studies different parameters were used for hEEG and intracranial electrical

stimulation.

The time window for the response was chosen in accordance with the response parameter

defined in Comolatti et al. (2019) for intracranial stereo-EEG (SEEG) recordings in humans. In those

human studies, 0.6 s was chosen because this was the approximate duration of the evoked SEEG

responses and of the differences in evoked activity between conditions, as determined by visual

inspection of the data in Comolatti et al. (2019). In order to use as far as possible the same methods in 4

rodents as in humans, for direct inter-species comparison, we used the same parameter, which is also

sufficient for including even the late phase-locked components of our data (see Figures 2A, 3A, 4A).

We have now clarified this point in the Methods section, on page 13. The only requirements for the

baseline window are that it should not be too short and it should be free of activity locked to the

stimulation, with an offset from zero; therefore a window of -0.5 to -0.005 s seemed to be appropriate.

In Comolatti et al. (2019), the baseline window comprised -0.3s to -0.01s, because of the limits given

by the stimulation frequency and the avoidance of the stimulation artifact. Finally, we now have

corrected Figures 1F, 4C and Extended Data 4-1C, in order to show the PCIst time course up to 0.6 s,

as suggested.

3- Figure 1 C vs 2A: Please clarify why the HF suppression during propofol does not seem to

coincide with a slow component of the ERP or the 5 individual traces that are shown. Is this specific

of propofol? It would be nice to also see more individual traces shown as supplementary material.

The background activity at the time of stimulus delivery is probably important, and the ERP may hide

this.

We have now added Extended Data Figures 2-1 and 2-2, showing 11 consecutive individual

traces in conditions of propofol anesthesia and wakefulness, respectively. These new figures show

data from the same animal and channel as Fig. 2A, allowing a direct comparison, and 2 seconds of

background activity is shown. Here we also show new analysis on low-pass filtered EEG responses (<

4 Hz; Butterworth, 3rd order) to visualize and quantify the slow ERP component. A slow component

was evoked by the electrical stimulation in both propofol anesthesia and wakefulness. With propofol,

the magnitude of the evoked slow component was indeed linearly related to the spontaneous slow

activity, but was slightly larger in amplitude. This was also indicated by the increased power of low

frequencies relative to baseline (1-4 Hz, dB > 0) that followed the stimulation (now this is also shown

in the spectrograms of Fig. 2A). In Extended Data Figure 2-1, we tested the temporal relation between

the slow component of the ERP and the HF suppression in propofol anesthesia. On average across

channels and rats, we found a temporal coincidence of the absolute peak of the slow component of the

ERP with the end point of the HF suppression. This result might help explain the stronger correlation

that we found between the ITPC drop time and the end point of HF suppression, also in relation to the

additional comment #7. Thanks to your comment, we also found a strong correlation between the

magnitude of the absolute peak of the slow component of the ERP and the deepest peak of HF

suppression across different intensities of stimulation, which resulted in a new panel in Fig. 3. The

method of analysis is described on pages 10, 11. These results are reported on pages 18, 19, 20. The

explanation of these results is addressed in the Discussion, on pages 27, 28.

4- Page 19; it is stated that ketamine was used “at a dose that abolished all motor responses to

painful stimuli (∼1.8 mg/kg/min i.v; Fig. 4A)”. Does this mean that the animal was immobile at all

times when the electrical stimuli were given? The anesthesia may abolish the response to pain stimuli,

but spontaneous movements may still happen and make the PCI measure variable. 5

Yes, at the experimental ketamine dosage we did not detect any obvious spontaneous

movement by visual inspection. This is now better explained in the Methods section, page 7, and in

the Discussion, page 25.

Although it is beyond the scope of this first paper for reproducing PCI in rats, the idea that

motor activity could influence perturbational complexity is interesting, especially during wakefulness.

Indeed, it has been shown that active, attentive and quiet wakefulness are associated with specific

fluctuation of membrane potential in cortical neurons that can affect performance in behavioral tasks

in mice (McGinley et al. 2015). It is conceivable that also cortical complexity might be influenced

accordingly. We are now planning new experiments with the goal to address the relation between

states of behavioural activity and PCI. This is now mentioned in the Discussion, on page 29.

5- Page 21 “Sevoflurane anesthesia in 10 rats produced results that were indistinguishable from

what we observed with propofol (Extended Fig. 1-4 and Extended Fig. 4-1)”. This statement is quite

misleading and should be qualified: PCIst values may be similar, but the ERPs are not.

We agree that the ERPs with sevoflurane vs. propofol look somewhat different in the

examples shown in the figures. However, there is variability between ERPs from different rats, even

for the same condition, and we could not find any systematic difference between ERPs with

sevoflurane vs. propofol, neither by eye nor by statistics. Thus, in addition to comparing the PCIst

values, we have now also tested several other features of the ERP, such as the magnitude and latencies

(starting and ending time points) of induced HF suppression, duration of the phase-locked response,

and the probability to detect a later increase in HF power and connectivity degree. We did not find

any statistically significant difference for each of these variables between sevoflurane and propofol

conditions. On page 22, we have now replaced the word “indistinguishable” with “similar”:

"Sevoflurane anesthesia in 10 rats produced results that were similar to what we observed with

propofol”. We have now also provided a clearer description of the ERP dynamic with sevoflurane on

page 22 and statistics are reported in the caption of the Extended Data Fig. 4-1. Results regarding

functional connectivity are reported on page 23 and in the caption of Extended Data Fig. 5-1. Results

from sevoflurane anesthesia are also commented on in the Discussion, pages 31, 32.

6- Page 21: Why was the ISPC averaged between 5 and 14 Hz and not between other

frequencies? The authors state that these frequencies “are implicated in long-range and feedback

interactions across visual areas.” But the two papers that they cite are from humans and NHPs. If this

translates to the rat, which I am concerned about, they should at least cite rodent studies here.

We thank the reviewer for pointing out this weak point in our references, and have now

replaced our previous references with articles that review experiments across species, including

rodents (Lisman & Jensen 2013; Hyafil et al. 2015). Such studies on rodents have mostly focused on

communication between hippocampus and frontal cortex, but studies in humans and monkeys also

provide evidence for cortico-cortical communication within the theta or theta-alpha range, in

connection with cognitive performance. Now we have also added more specific references for mice

and monkeys (Tamura et al. 2017; Liebe et al. 2012). 6

Overall, we decided to test phase-based functional connectivity (ISPC) within the theta (5-8

Hz) - alfa (9-14 Hz) ranges for empirical and theoretical reasons. As shown in the example traces

below, the phase-locking across trials in the theta-alpha range was most long-lasting in wakefulness,

thus the frequency range 5-14 Hz was well suited for testing ISPC in a later time window, far from

the stimulus onset (0.18-0.3 s). This rational and the average ITPC drop time across channels and rats

during wakefulness are now reported on page 22 and mentioned in the Discussion, page 30. We did

not use a broad band range, e.g. 5-40 Hz, because ISPC is a score that can assume negative values,

after baseline correction (subtraction of baseline average scores, see Methods, page 12). Thus,

potential negative ISPC scores of higher frequencies might affect positive ISPC scores detected at

lower frequencies, when averaged together in a broad band range. The theoretical reason relates to the

role that theta-alpha oscillations might have in supporting communication across distant brain regions

during wakefulness and cognitive tasks, which has been suggested by several authors, also by nesting

high frequency activity (Lisman & Jensen 2013; Hyafil et al. 2015). For these reasons, the θ-α range

seemed ideal to test the functional connectivity in a network that engages in long-range interactions in

response to stimulation. This rationale is now better explained in the Discussion, page 30.

FIGURE 1: Above, an example of mean ERP in response to electrical stimulation (onset at 0 s), with 5

overlapped single trials, from one channel, during wakefulness. Below. Inter-trial phase clustering

(ITPC) of the ERP reported above (90 trials) is shown for each frequency tested (from 1 to 40 Hz).

The horizontal dotted lines highlight the theta band (5-8 Hz) and alpha band (9-14 Hz). It can be seen

that the last ITPC scores > 0 after stimulation are within the theta-alpha frequency range.

7- I wonder, in figure 3D, why they correlate ITPC drop with the end of the putative OFF

period, rather than the start. Shouldn’t it be the start of the OFF period that causes this drop? Their

figures seem to suggest so. I guess it is because previous studies have correlated ITPC drop with the

end of the OFF period?

We tested the correlation of the ITPC drop time with different time points of the period with

HF suppression. We did not find any significant correlation with the onset of HF suppression, while

the strongest temporal correlation was with the end of the suppression period; therefore this was 7

originally chosen for Fig. 3. We now show all the tested correlations in the new Extended Data Figure

3-1, allowing an in-depth examination. Now, Fig 3 shows the correlation between the ITPC drop time

and the latency of the maximal HF suppression (most negative peak of relative powers in range 20-40

Hz), which is more coherent with the literature (Pigorini et al. 2015; Rosanova et al 2018). For

completeness, we also decided to add a new tested correlation between the magnitudes of the maximal

HF suppression and the peak of the slow component of the ERP (see also response to additional

comment #3). The results of this new analysis are reported on pages 19, 20. Finally we propose to

replace the panel A of figure 3 with a new one that shows the HF spectrogram and the ITPC from all

EEG channels, for a better representation. The new analysis and the correlation between the ITPC

drop time and the latency of the end of HF suppression are discussed on page 28.

8- The authors should avoid calling their periods of HF suppression “OFF periods” without

MUA, though I have no problem if they want to point out the relationship between the two phenomena

in the discussion.

We understand this concern and we have now replaced “OFF period” with “HF suppression

period” in the Methods and Results sections and in the figure captions. The relation between OFF

periods and HF suppression is addressed in the Discussion, on pages 27, 28.

9- Perhaps I missed it, but I could not find any discussion of why the authors targeted L2/3

rather than, say, L5. It should be clearly stated that “deeper” in this study still means superficial

layers.

We targeted layer 2/3 because it mainly contains pyramidal neurons that project to other

ipsilateral and contralateral cortical areas (Petreanu et al., 2007; Harris and Shepherd, 2015; Gerfen et

al., 2018), thus it seemed ideal to investigate cortico-cortical integration. This is now stated in

Discussion, page 25. Along the dorsal-ventral axes, the tips of the bipolar stimulating electrodes were

mainly located within layer 2/3 across rats. However, in one rat, one tip of the bipolar electrode was

placed in layer I (0.05 mm from cortical surface, see Fig. 6), and in another rat one tip of the bipolar

electrode was placed in the upper part of layer 5 (0.92 mm from cortical surface, see figure below).

Thus, across rats, the locations of the stimulating electrodes spanned from layer 1 to layer 5 (upper

part). This is now more clearly stated in the results section, page 16, and is commented in the

Discussion, on page 31.8

FIGURE 2: Coronal brain section (Nissl staining) from the rat with the deepest location of the bipolar

stimulating electrode in the right M2. The right tip of the stimulating electrode was located in the

upper part of layer 5 (layer with bigger cell bodies and lower density). Scale bar = 1 mm.

10- Page 25 “and it has been suggested that the same mechanisms may be triggered by the initial

response to cortical stimulation.” Please provide the references.

Thank you for the comment. References have now been added
