# Supplementary figures and images for: General Anesthesia Disrupts Complex Cortical Dynamics in Response to Intracranial Electrical Stimulation in Rats
Source: eNeuro. 2021 Aug 4;8(4):ENEURO.0343-20.2021. doi: 10.1523/ENEURO.0343-20.2021 (PMC8354715; doi:10.1523/ENEURO.0343-20.2021)

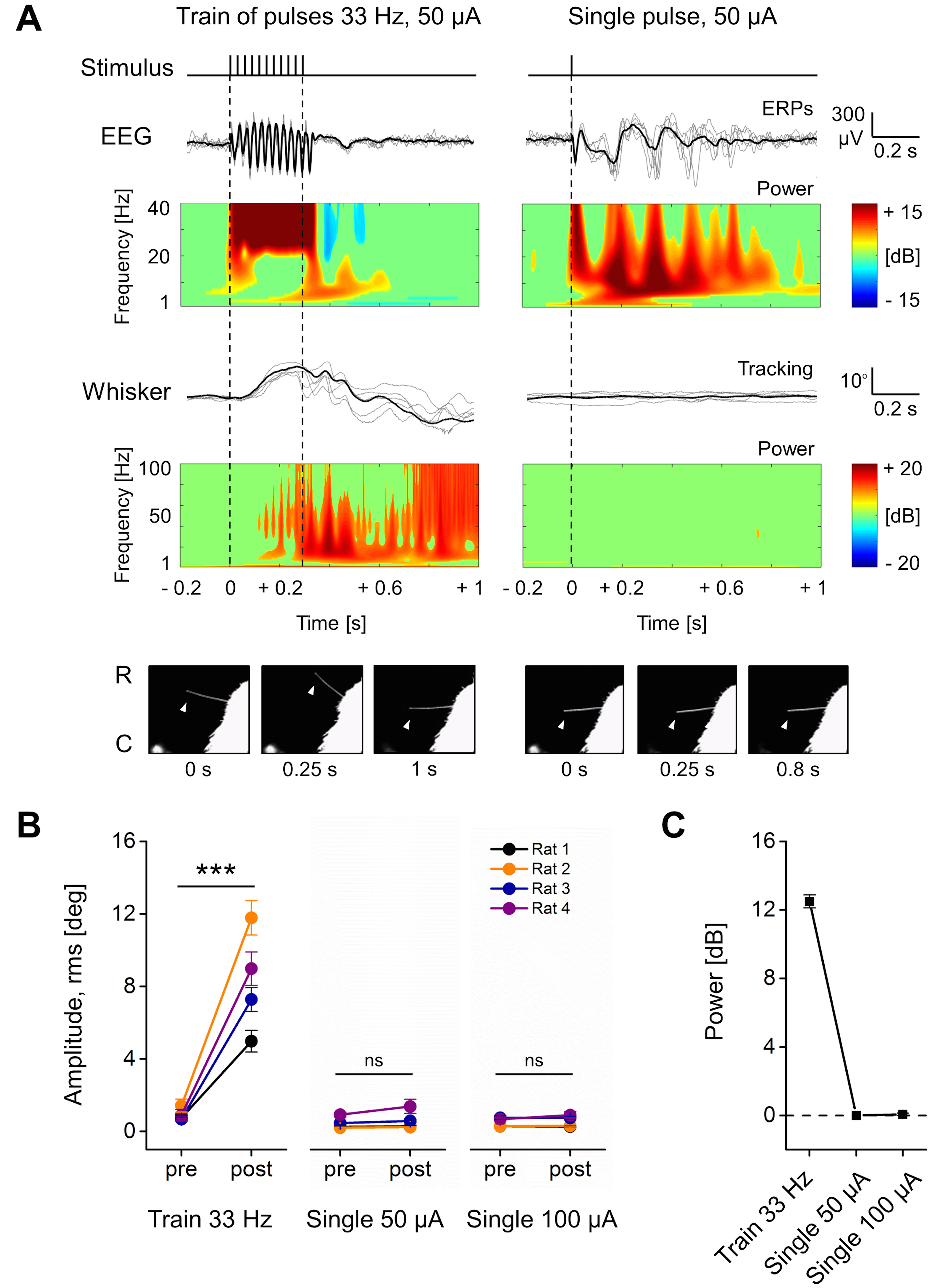

Supplement: Extended Data Figure 1-1 — Single pulse electrical stimulation of M2 did not trigger detectable whisker movements. A, up, Example of five consecutive ERPs (grey traces) with ensemble average (black) during wakefulness, in response to two different electrical stimulations of right M2 (left, train of pulses of 1 ms, 50 μA, rate 33 Hz, train duration 0.3 s; right, single pulse, 1 ms, 50 μA; 21 stimulations delivered at 0.2 Hz) with relative spectrogram below. Middle, Tracking of the angular movement of left C1 whisker from the same rat, in response to stimulations (five consecutive single motor responses in grey and ensemble average in black) with relative spectrogram. Bottom, View from above of the rat’s snout and C1 whisker (R-C: rostral-caudal) at three time points. White arrowheads indicate whisker positions, while the white dot in the bottom-left side represents the onset of the electrical pulse. Only the train stimulation is able to trigger a detectable motor response of the left C1 vibrissae, spanning in a broad frequency range. B, The rms amplitude of the angular motor response to the train stimulation and to the single pulse stimulation at 50 and 100 μA is quantified at the level of each animal (n = 4 rats). The rms amplitudes of 0.25 s after the stimulus offset (post) were compared with those obtained from 0.25 s before the onset of stimulation (pre; baseline, from –0.5 to –0.25 s) for all the 21 evoked responses (Wilcoxon S-R test; Train stimulation, for all rats: p = 5.957 × 10^−5^; single pulse 50 μA, for rat 1: p = 0.715, for rat 2: p = 0.114, for rat 3: p = 0.170, for rat 4: p = 0.068; single pulse 100 μA, for rat 1: p = 0.931, for rat 2 and rat 3: p = 0.664, for rat 4: p = 0.339). Averaged rms values across rats have been also calculated. Train stimulation: prestimulus 0.94 ± 0.17°, poststimulus 8.25 ± 1.43°; single pulse 50 μA: prestimulus 0.46 ± 0.16°, poststimulus 0.62 ± 0.26°; single pulse 100 μA, prestimulus 0.50 ± 0.12°, poststimulus 0.56 ± 0.16°). C, In order to be able to d [file enu-eN-NWR-0343-20-s01.tif]

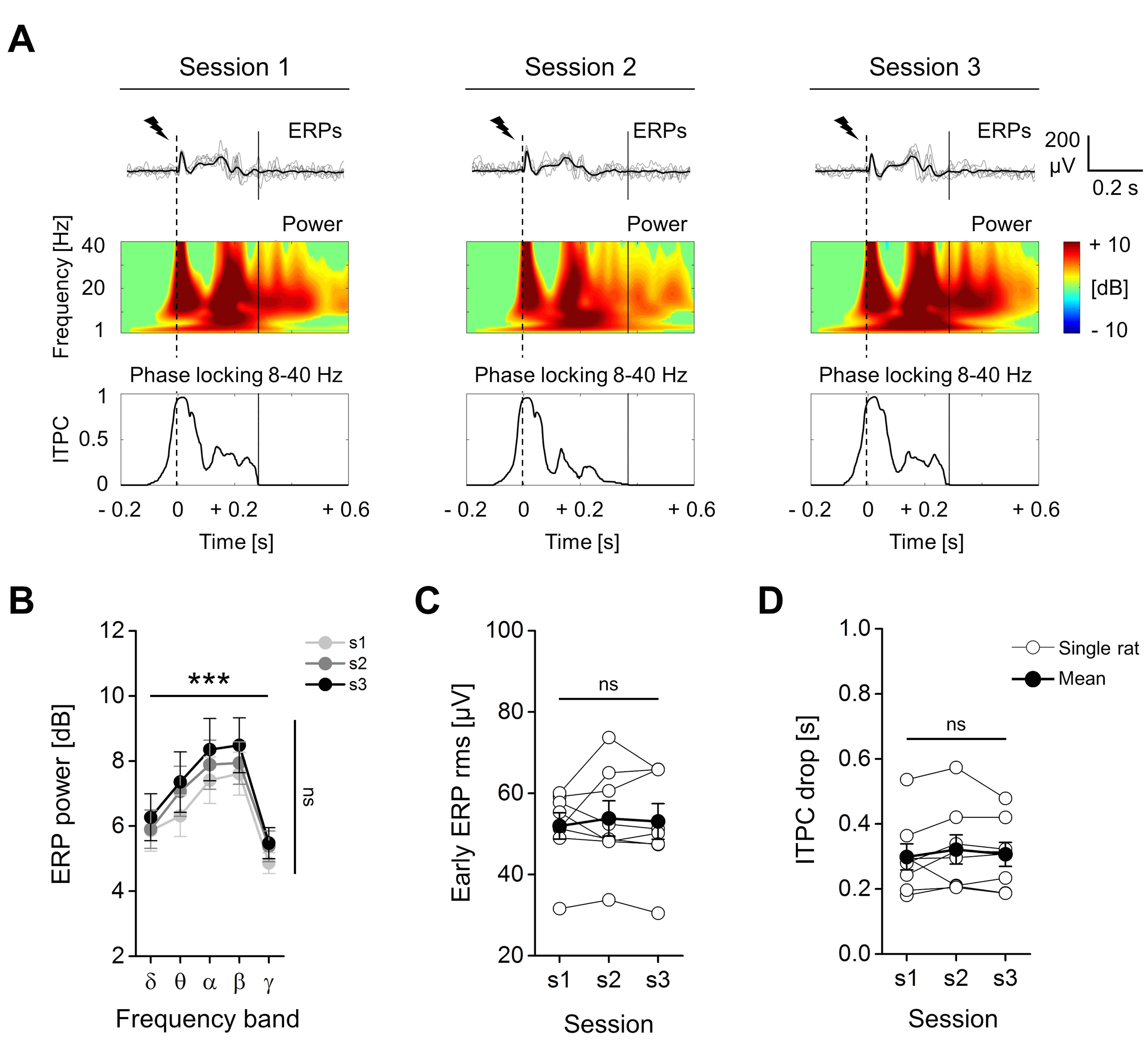

Supplement: Extended Data Figure 1-2 — Single pulse electrical stimulation of M2 triggered reproducible ERPs that were reliable across different recording sessions. B, Example of five consecutive ERPs (grey traces) with ensemble average (black) during wakefulness, in response to single pulse electrical stimulations of M2 (dashed line, 50 μA, 1 ms) from the same rat and channel, in different recording sessions, performed in different days. Below, The relative spectrograms are shown, with the ITPC averaged across frequencies (range 8–40 Hz). ITPC quantify the degree of reproducibility of the ERPs (phase-locking across trials). The drop time of ITPC is highlighted by a black line and indicates the duration of the phase-locked response induced by the stimulus (ITPC drop). B–D, Quantification of the reliability of ERPs in response to single pulse stimulations from n = 8 rats, across 3 recording sessions (s1, s2, s3) performed in different days (∼4 d between each session). B, the relative mean spectral power of the ERPs (up to 0.5 s) differed across frequency bands (Friedman test, p = 1.189 × 10^−5^), with a peak in alpha (8–14 Hz) and beta (15–25 Hz) ranges, but no difference across sessions was identified (Friedman test, p = 0.088). C, The cortical excitation in response to stimulation was measured as the rms amplitude of the first deflection of the mean ERPs (Early ERP rms, up to 0.05 s from stimulus onset) and no variation across different days was detected (Friedman test, p = 0.687). D, Likewise, no significant variation across sessions was detected in the time of ITPC drop (Friedman test, p = 0.072). Download Figure 1-2, TIF file. [file enu-eN-NWR-0343-20-s02.tif]

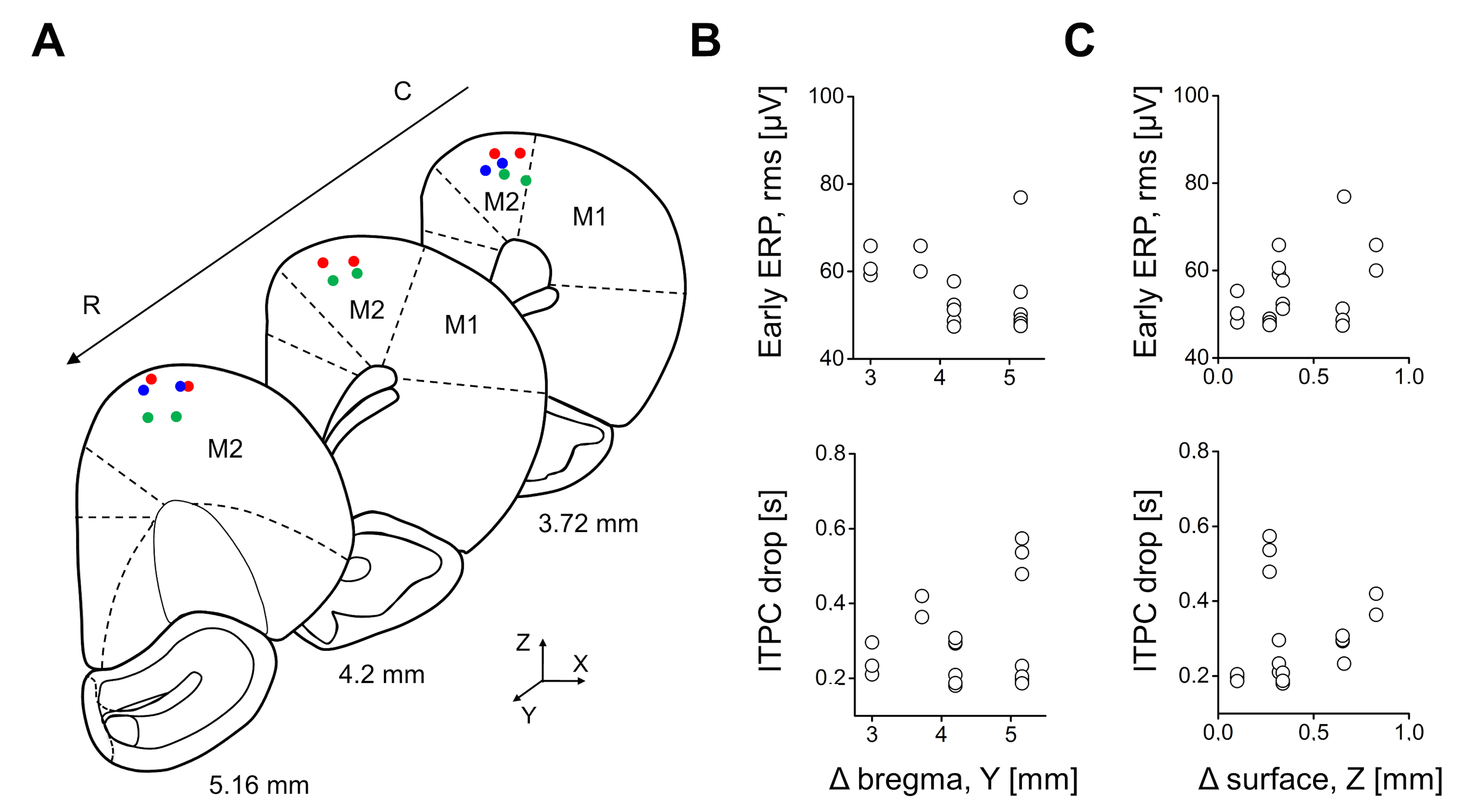

Supplement: Extended Data Figure 1-3 — Amplitude of ERPs and duration of phase-locked response were not related with the stimulus location in M2. A, Representation of coronal sections of rat brain with actual position of chronically implanted bipolar electrodes in right M2 from eight rats (dots represent the two poles of the bipolar electrode and each rat is color coded in each slice). Coordinates have been measured from Nissl-stained coronal sections of rat brains. Among the tested animals, stimulating electrodes covered a cortical area from ∼5 to ∼3 mm with respect to bregma in rostral-caudal direction (R-C, y-axes, mean position 4.38 ± 0.25 mm) and a depth range from ∼0.1 to ∼0.8 mm calculated from cortical surface (Z axes, mean position 0.47 ± 0.09 mm, averaged values for each electrode between the two poles), mainly corresponding to Layer II/III. On average the 2 electrode poles were separated by 0.46 ± 0.03 mm in the medial-lateral direction (x-axes). B, C, mean rms amplitude of the first deflections of the ERPs (up to 0.05 s from stimulus onset, up) and mean duration of phase-locking among subsequent ERPs (ITPC drop, 8–40 Hz, bottom) are plotted for all rats (n = 7) and recording sessions (1 to 3 recordings for each rat) during wakefulness against the position of the stimulating electrode along the y-axes (B) and along the z-axes (C). Possible correlations with the position of stimulating electrode have been tested and no significant relation was detected between the Early ERP rms amplitude and the position of electrodes along the y-axes (B, up; linear fit, p = 0.121, R2 = 0.144) or along the Z axes (C, up; linear fit, p = 0.122, R2 = 0,143). No significant relation was identified between the ITPC drop and the position of electrodes along the y-axes (B, bottom; linear fit, p = 0.35, R2 = 0.055) or along the Z axes (C, middle; linear fit, p = 0.122, R2 = 0.143). Download Figure 1-3, TIF file. [file enu-eN-NWR-0343-20-s03.tif]

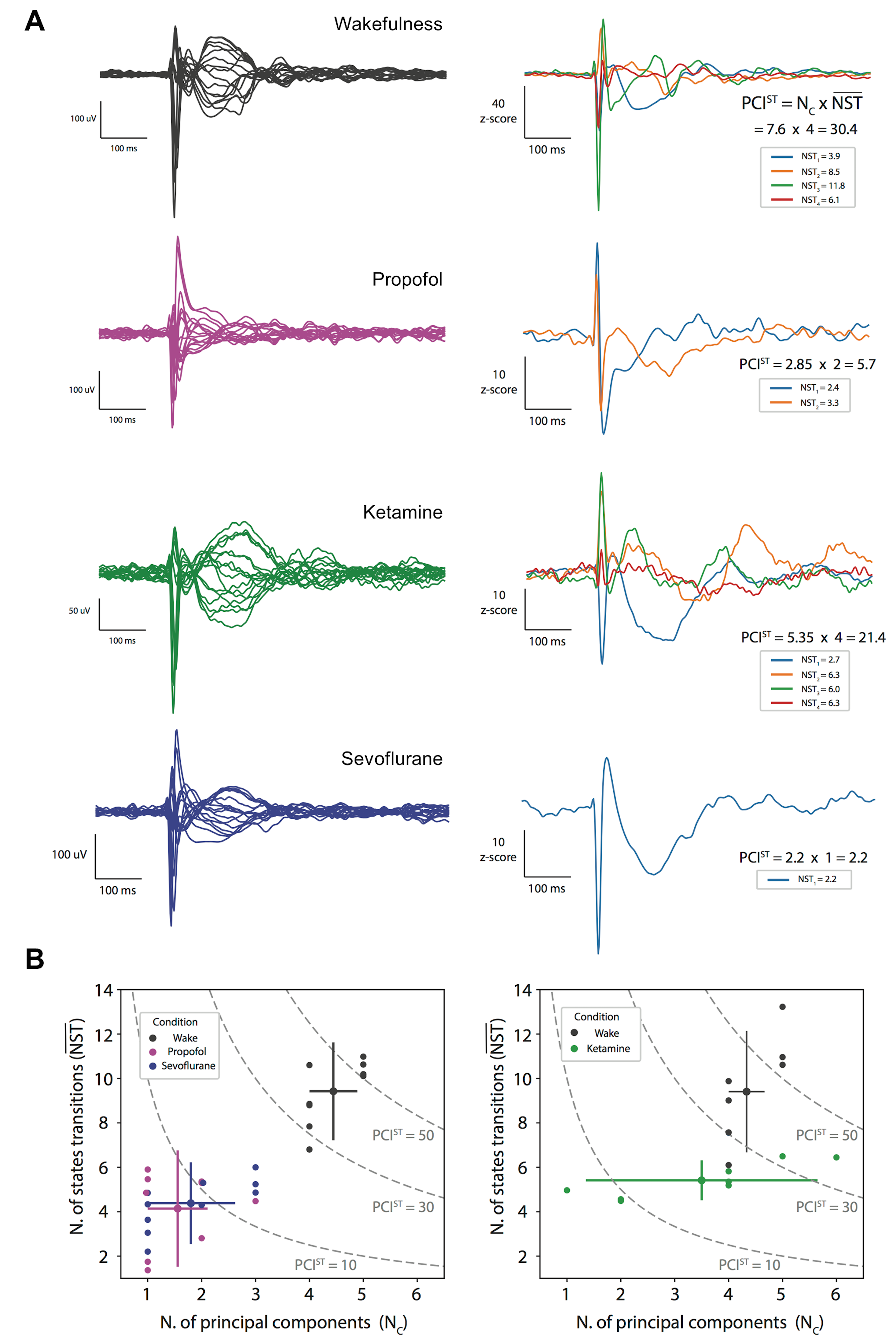

Supplement: Extended Data Figure 1-4 — PCIST can be decomposed in average NST and number of principal components (NC). A, ERPs and corresponding PCIST decomposition into principal components and state transitions (NST) during wakefulness and during propofol, ketamine and sevoflurane anesthesia (top to bottom) from one animal. Left, Butterfly plots with evoked responses to electrical stimulation of right M2. Right, PCIST can be decomposed as the product between the number of principal components (NC), an estimate of the spatial diversity of the signal, and the average NST, corresponding to the temporal differentiation of the signal, i.e., PCIST = average NST x NC. The panel shows each principal component of the ERPs that together account for 99% of the variance in the response (time range: 0.08–0.6 s), with the corresponding values of state transitions in the legend box; above it, the PCIST value computed as the product of average NST and number of principal components (NC). B, Scatter plots with average NST and number of principal components for all animals with corresponding group average, and 5 and 95 percentiles (NC). Left, Rats during wake (n = 9), propofol (n = 9), and sevoflurane (n = 10). Right, Rats during wake (n = 8) and ketamine (n = 8). Also shown for reference are the contour lines (dotted gray lines) with different PCIST values. Download Figure 1-4, TIF file. [file enu-eN-NWR-0343-20-s04.tif]

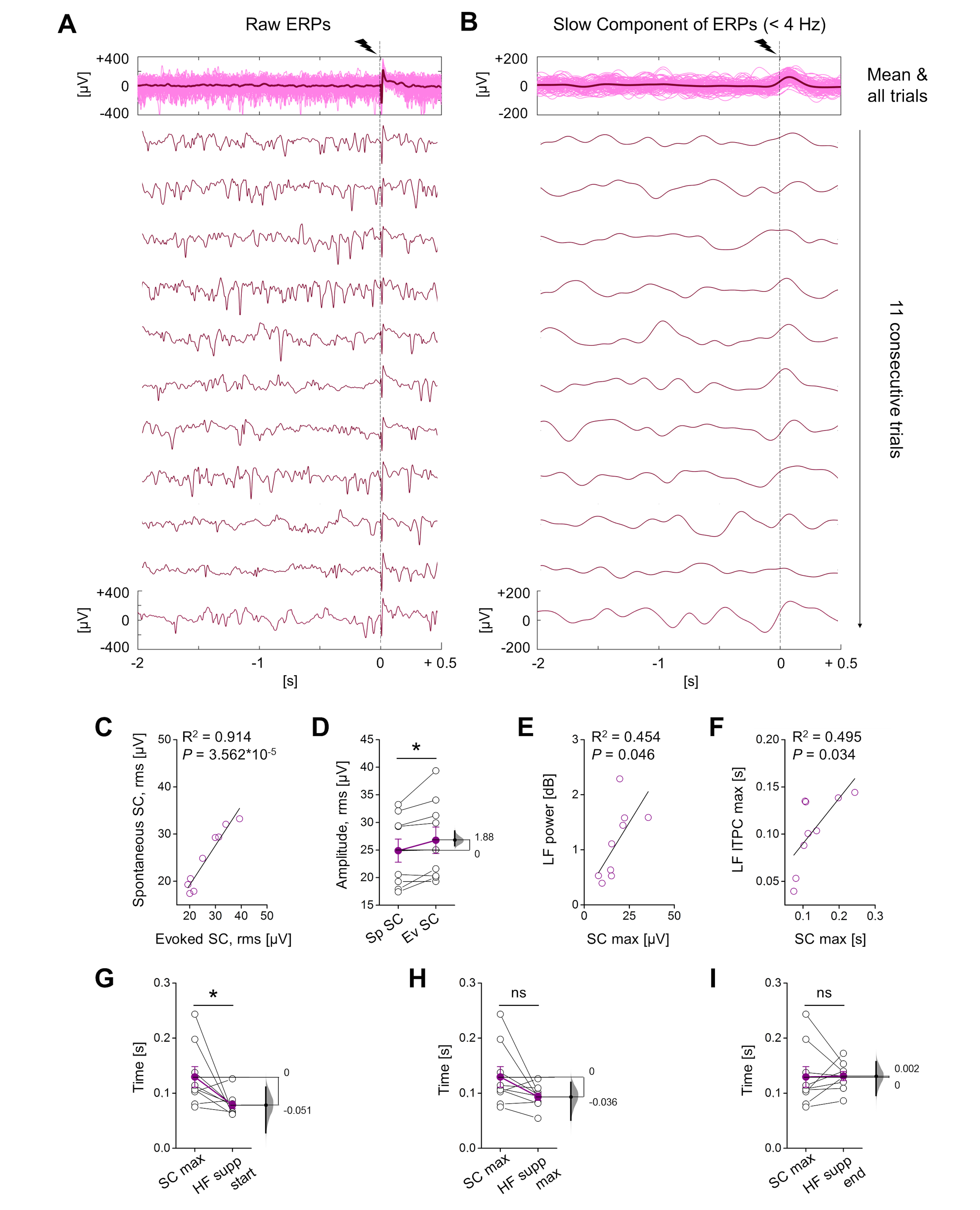

Supplement: Extended Data Figure 2-1 — Single trials and SC of the ERP during propofol anesthesia. Example of raw EEG responses to single pulse electrical stimulation (1 ms, 50 μA; dashed line at 0 s; A) and corresponding low-passed responses (<4 Hz, Butterworth filter, 3rd order; right; B) from one rat during propofol anesthesia. The mean ERP (bold) and all 90 single trials from the same frontal channel (M2) are overlaid and shown at the top, while 11 consecutive single trials are shown below for clarity. The responses are from the same animal and channel as in Figure 2. The low-passed responses are used to visualize and quantify the SC (<4 Hz) of the ERP, which was consistently evoked by the stimulus, in nine rats. C, The spontaneous slow wave activity before the stimulation might influence the SC of the response, since we found a linear, strong, positive correlation between the amplitude of the spontaneous SC and the amplitude of the evoked SC (linear fit, p = 3.562 × 10^−5^, R2 = 0.914). The amplitude of the spontaneous SC (sp SC) for each single trial was quantified with the rms of 2-s baseline (range –2 to 0 s). The values of single trials were then averaged across trials and channels, obtaining estimates for all rats, which were used for assessing the correlation. The same procedure was adopted to quantify the amplitude of the evoked SC (ev SC), within a shorter time range after stimulation (from 0 to 0.3 s) to only include the evoked response. D, The evoked SC was slightly, but significantly higher in amplitude than the spontaneous SC (sp SC: 24.88 ± 2.09 μV, ev SC: 26.76 ± 2.40 μV; mean difference = 1.88 [95% CI: 0.83, 3.5]; Wilcoxon S-R test, p = 0.019). E, Consistently, the maximal peak amplitude of the evoked SC (SC max) was linearly and positively correlated with the maximal increase of LF power (1–4 Hz) with respect to baseline (linear fit, p = 0.046, R2 = 0.454). SC max was computed for each rat, from the mean low-passed ERP of each channel, as the maximal absolute amplitude value after the [file enu-eN-NWR-0343-20-s05.tif]

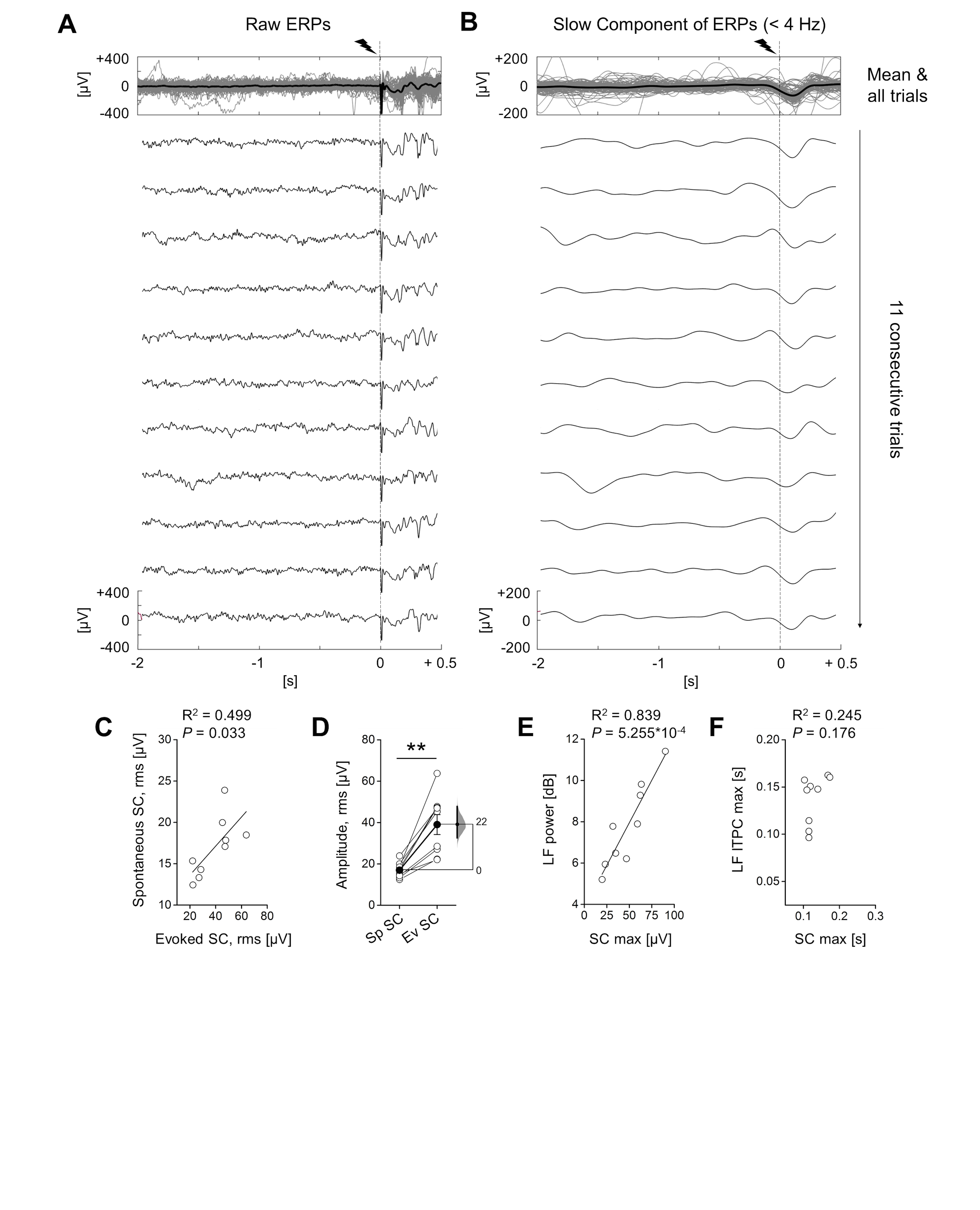

Supplement: Extended Data Figure 2-2 — Single trials and SC of the ERP during wakefulness. Example of raw EEG responses to single pulse electrical stimulation (1 ms, 50 μA; dashed line at 0 s; A) and corresponding low-passed responses (<4 Hz, Butterworth filter, 3rd order; right; B) from one rat during wakefulness. The mean ERP (bold) and all 90 single trials from the same frontal channel (M2) are overlaid and shown at the top, while 11 consecutive single trials are reported below for a better representation. The responses are from the same animal and channel reported in Figure 2. The low-passed responses are used to visualize and quantify the SC (<4 Hz) of the ERP, which was consistently evoked by the stimulus, in nine rats. C, The spontaneous activity before the stimulation might influence the SC of the response, since we found a linear, positive correlation between the amplitude of the spontaneous SC and the amplitude of the evoked SC (linear fit, p = 0.033, R2 = 0.499). The amplitude of the spontaneous SC (sp SC) for each single trial was quantified with the rms of 2-s baseline (range –2 to 0 s). The values of single trials were then averaged across trials and channels, obtaining an estimation for all rats, which were used for assessing the correlation. The same procedure was adopted to quantify the amplitude of the evoked SC (ev SC), within a shorter time range after stimulation (from 0 to 0.3 s) to only include the evoked response. D, The evoked SC was significantly higher in amplitude than the spontaneous SC (sp SC: 16.96 ± 1.20 μV, ev SC: 39.00 ± 4.82 μV; mean difference = 22.0 [95% CI: 15.4, 30.6]; Wilcoxon S-R test, p = 0.004). The floating axis on the right shows the mean difference between conditions and the effect size is reported as bootstrap resampling distribution of mean difference, with 95% CI represented by the bold black vertical line. E, Consistently, the maximal peak amplitude of the evoked SC (SC max) was linearly and positively correlated with the maximal increase of LF power (1–4 [file enu-eN-NWR-0343-20-s06.tif]

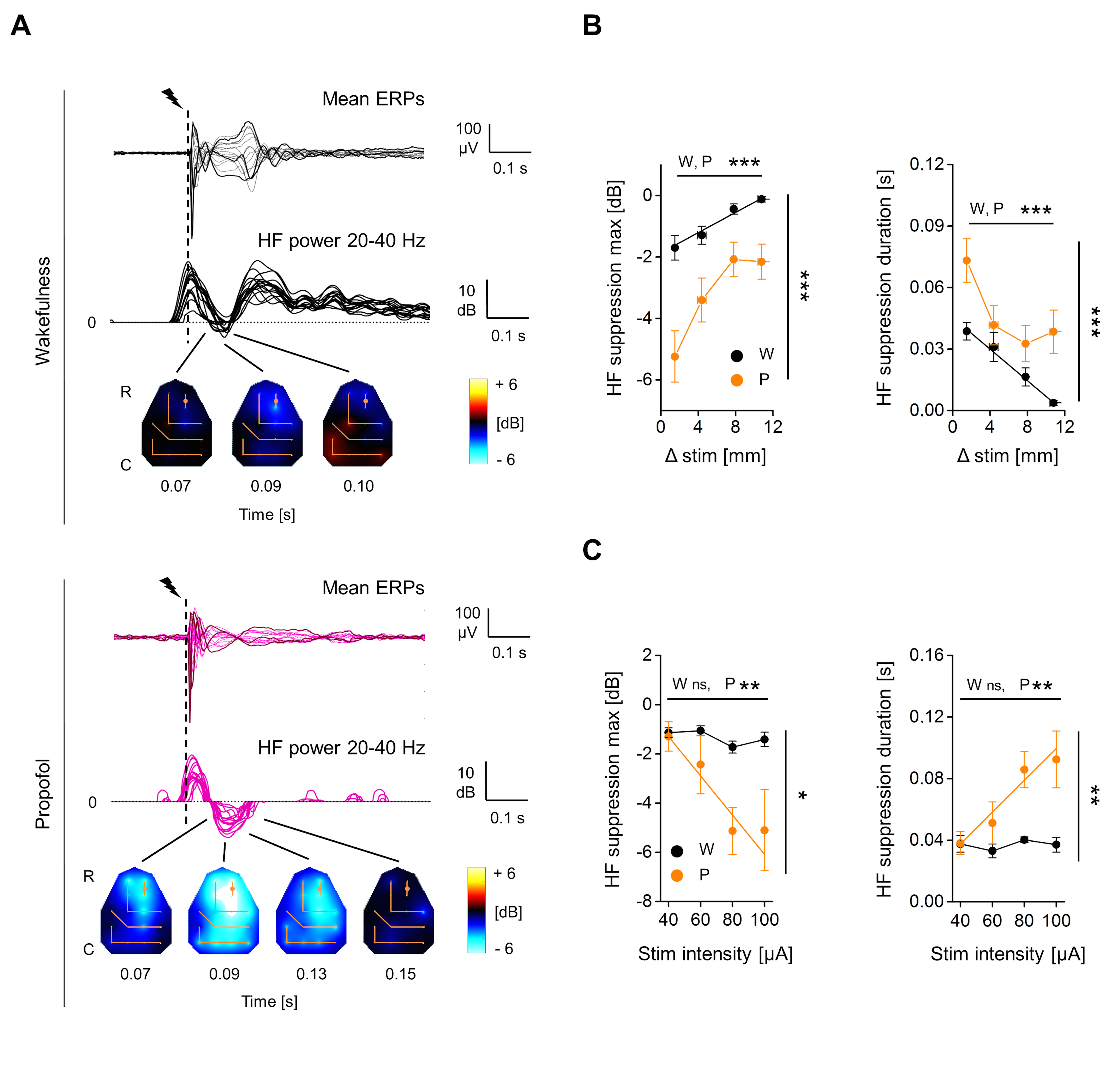

Supplement: Extended Data Figure 2-3 — The suppression of HF power during wakefulness was weaker and briefer than the one with propofol, and was not affected by increasing stimulus intensity. A, Epidural EEG activity in response to single pulse electrical stimulation (1 ms, 50 μA; dashed line) of the right M2, from the same rat during wakefulness (up) and during propofol anesthesia (bottom). The electrophysiological traces in the butterfly plot represent the superimposition of ensemble averages of ERPs (n = 90 trials) from all recording electrodes (mean ERPs from three channels are in bold for highlighting difference in complexity, same channels between wakefulness and propofol conditions). Below, The temporal dynamic of HF power (averaged in the range 20–40 Hz) from all the channels is also reported (dotted horizontal line indicates 0 dB). The color maps show the color-coded interpolation of HF power during the suppression period (window of time when HF power is <0 dB) over the scalp in a rostro-caudal orientation (R-C). Orange lines represent the isodistance lines that connect channels with similar spatial distance from the stimulating electrode (orange dot; M2 right). To assess the relation between HF suppression and the spatial distance from the stimulus, we averaged values from channels that were clustered in four isodistance lines, at 1.5 ± 0, 4.38 ± 0.47, 7.8 ± 0.17, and 10.8 ± 0.40 mm far from the site of stimulation. B, Quantification of the negative peak of HF power during suppression (deepest suppression, HF suppression max; left) and duration of the suppression of HF power (right), averaged within isodistance lines and across rats (n = 9 animals) during wakefulness (W) and propofol anesthesia (P). During wakefulness, HF suppression max linearly approaches to 0 dB from more negative values while increasing the distance from the site of stimulation (Friedman test, p = 6.022 × 10^−5^; linear fit, p = 0.021, R2 = 0.959). Also, during propofol anesthesia a relation between HF suppression max and dis [file enu-eN-NWR-0343-20-s07.tif]

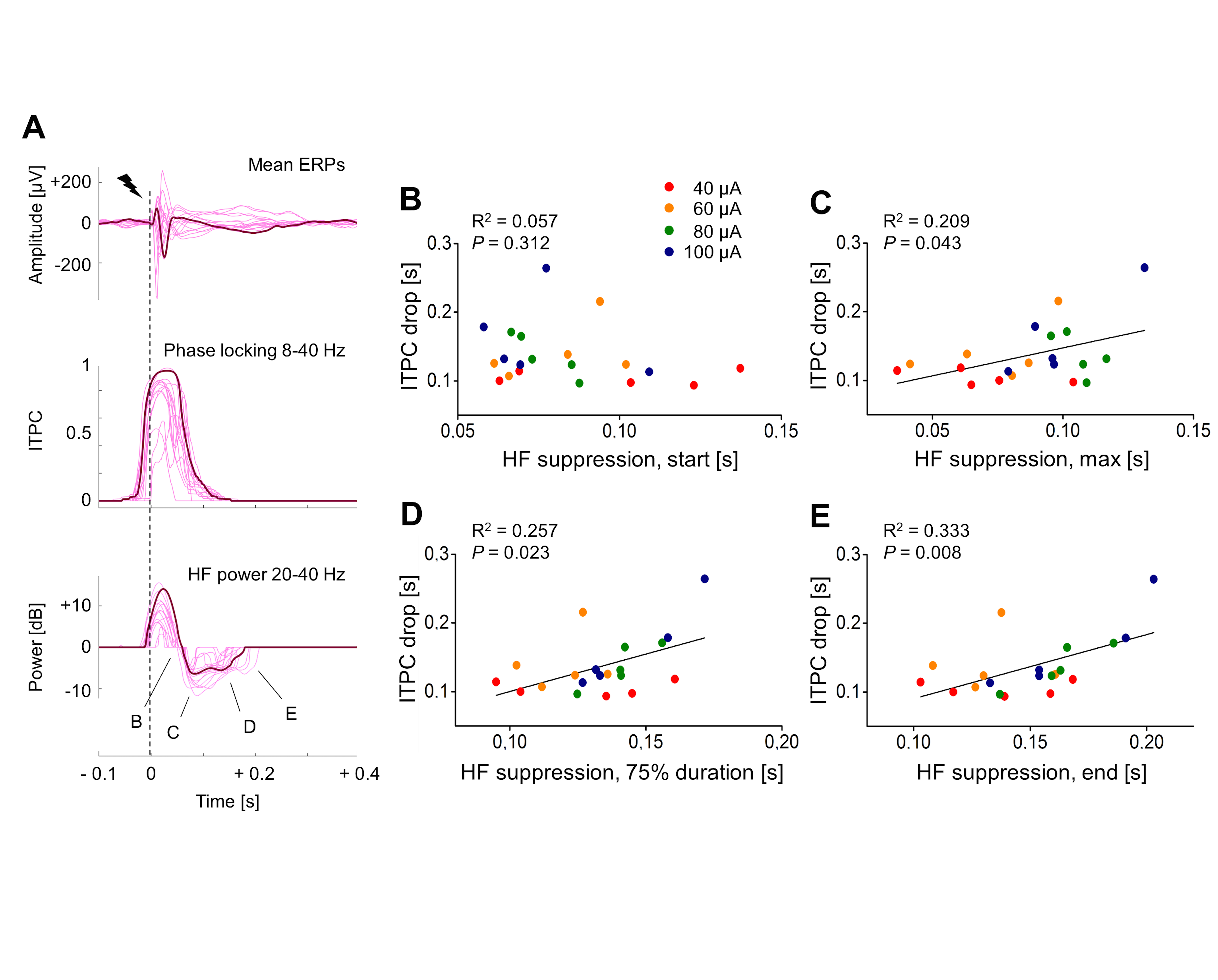

Supplement: Extended Data Figure 3-1 — ITPC drop time better correlated with the end phase of HF suppression. A, Mean ERPs from the same rat in response to single pulse electrical stimulations (1 ms, 60 μA; dashed lines) during propofol anesthesia (up), with corresponding phase-locking temporal dynamics averaged in broad frequency range, 8–40 Hz (ITPC; middle), and temporal dynamic of HF power, averaged in range 20–40 Hz (bottom). The three plots report the superimposed activity from all 16 recording electrodes, with the dynamics from the same parietal channel (RS) in bold for clarity. Letters B, C, D, and E, respectively, approximate the onset of the first HF power <0 dB (HF suppression, start), the latency of the most negative peak of HF power (HF suppression, max), the time point corresponding to the 75% of the HF suppression duration (HF suppression, 75% duration), and the last time point of HF power <0 dB (HF suppression, end). These time points have been calculated within the time range 0–0.3 s, averaged across channels for each rat (n = 5) and stimulation intensity (40, 60, 80, 100 μA), and plotted against the respective mean ITPC drop time to assess correlations in panels B–E (values from different stimulus intensities are color coded). B, The ITPC drop time did not correlate with the onset of HF suppression (linear fit, p = 0.312, R2 = 0.057). However, linear positive correlations were found with HF suppression, max (C; linear fit, p = 0.043, R2 = 0.209), with HF suppression, 75% duration (D; p = 0.023, R2 = 0.257) and with the HF suppression, end (E; p = 0.008, R2 = 0.333). It should be noted how both the statistical significance and the coefficient of determination gradually increased by assessing the correlation of ITPC drop time against later time points of HF suppression (lower p and higher R2). Thus, the best temporal correlation was between the ITPC drop and the end of the HF suppression. Download Figure 3-1, TIF file. [file enu-eN-NWR-0343-20-s08.tif]

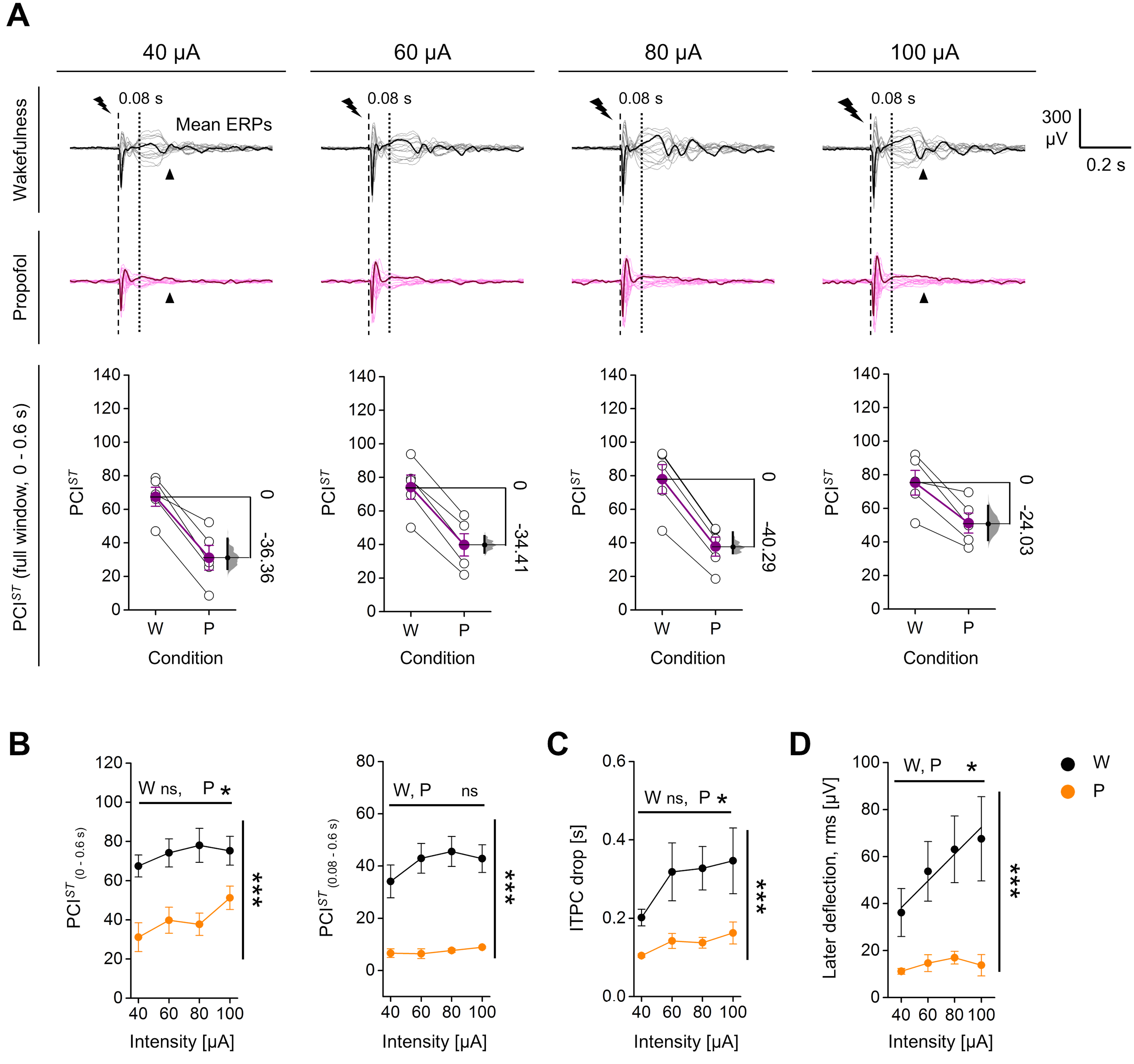

Supplement: Extended Data Figure 3-2 — Perturbational complexity in relation to incremental intensity of stimulation. A, epidural EEG activity in response to single pulse electrical stimulations of increasing intensities (1 ms, 40, 60, 80, and 100 μA; dashed line) of the right M2 from the same rat during wakefulness (up) and during the exposure to propofol anesthesia (middle). The electrophysiological traces represent the superimposition of ensemble averages of evoked related potentials from all the 16 recording electrodes distributed all over the skull. One averaged ERP from the same channel across condition has been highlighted in bold for better illustrating the difference in cortical complexity. The vertical dotted lines indicate 0.08 s, which approximates the rising of the HF suppression duration. PCIST scores obtained from the whole window of the evoked response (0–0.6 s) are shown for all animals (white dots) and averaged across rats (n = 5 rats; purple dots) for all intensities of stimulation and condition (below). PCIST always decreased in all animals, from wakefulness (W) to propofol anesthesia (P), regardless of the intensity of stimulation. The floating axis on the right of each group comparison shows the mean difference between conditions. The effect size is reported as bootstrap resampling distribution of mean difference, with 95% CI represented by the bold black vertical line. B, We otherwise observed a significant variation of PCIST (in full-time window 0–0.6 s; left) during propofol anesthesia in relation to stimulus intensity (Friedman test, p = 0.029), which was not detectable during wakefulness (Friedman test, p = 0.062). Overall PCIST during wakefulness was higher than during propofol anesthesia (Friedman test, p = 1.55 × 10^−5^). No relation with stimulus intensity was detected by calculating PCISTfrom 0.08 to 0.6 s and therefore by excluding the first response to the electrical stimulation, before the rising of the HF suppression (right). No significant variation of PCIST has been ob [file enu-eN-NWR-0343-20-s09.tif]

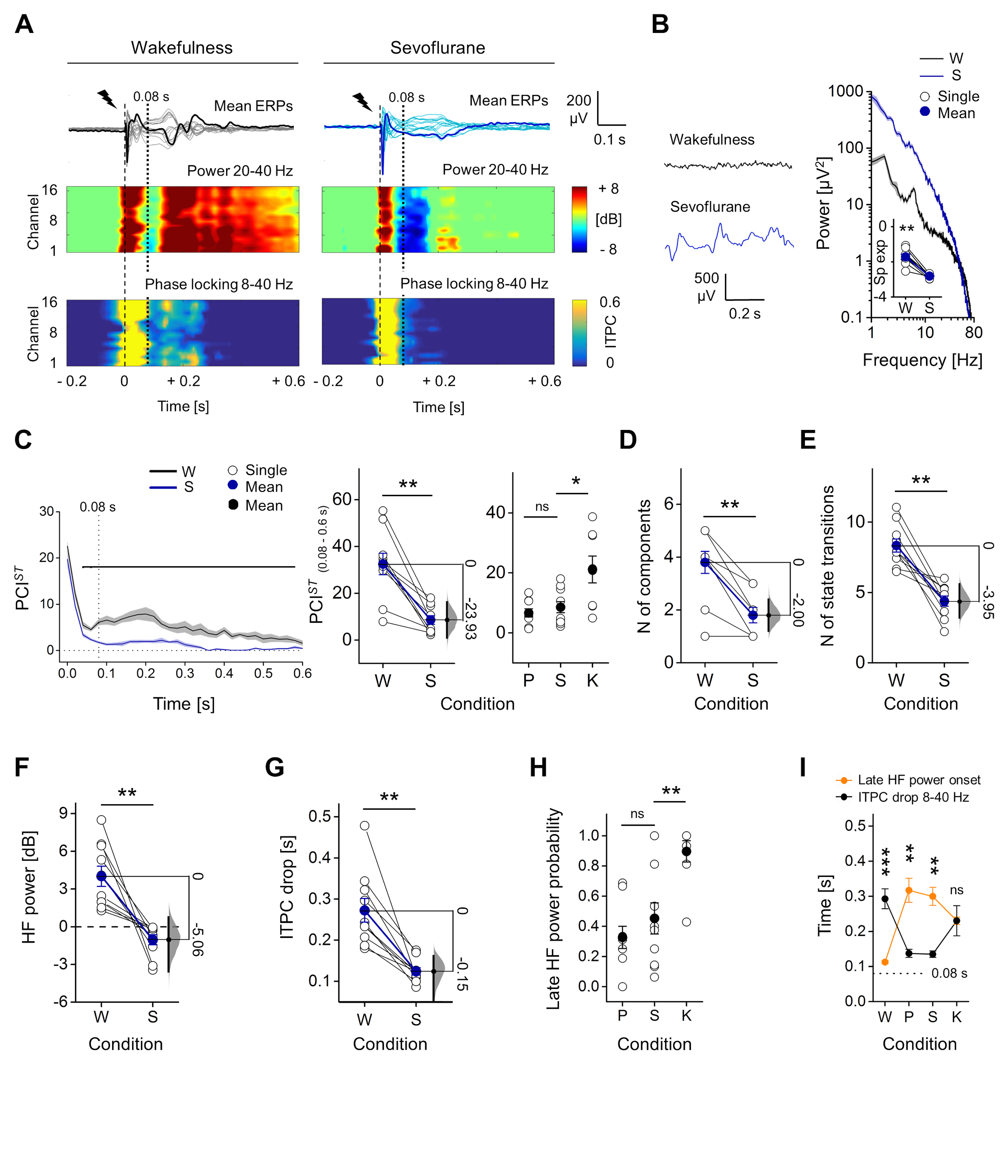

Supplement: Extended Data Figure 4-1 — PCIST, suppression of high frequencies and phase-locking of ERPs in response to electrical stimulation during sevoflurane anesthesia. A, Superimposition of mean ERPs from all electrodes in response to single pulse stimulation (1 ms, 50 μA; dashed line) from the same rat during wakefulness (W) and sevoflurane (S; ∼2.6%) anesthesia (same rat from Fig. 4 to facilitate comparisons). One averaged ERP from the same channel (M2) is in bold to highlight differences in complexity. Spectrograms of HF power and ITPC for all channels are shown below. Vertical dotted line at 0.08 s indicates the average time of HF suppression onset across rats. B, Spontaneous EEG (left) and relative mean periodograms (shades represent SEM; right) are reported from one rat during wakefulness and sevoflurane anesthesia. Spectral exponents from all rats are also reported (inset). The spectral exponent dropped from wakefulness to sevoflurane anesthesia, highlighting a redistribution of power towards slow frequencies (n = 9 rats; wakefulness: –1.71 ± 0.15, sevoflurane: –2.81 ± 0.04; Wilcoxon S-R test, p = 0.004). C, left, Time courses of mean PCIST in conditions of wakefulness and sevoflurane anesthesia (n = 10 rats; shades represent SEM; horizontal lines indicate time periods with statistical differences between conditions, Wilcoxon S-R test p < 0.05; from 0.58 s, the PCISTvalues alternated between being significantly different and not significantly different between conditions, while from 0.8 s, we found no further period with statistically significant differences until 1.1 s). Right, PCISTin range 0.08–0.6 s is reported for each rat and condition. PCIST(0.08–0.6 s) significantly dropped from wakefulness to sevoflurane anesthesia (wakefulness: 32.50 ± 4.60, sevoflurane: 8.57 ± 1.85; mean difference = –23.93 [95% CI: –31.79, –16.47]; Wilcoxon S-R test, p = 0.002). Moreover with sevoflurane, PCIST(0.08–0.6 s) was similar to what we obtained with propofol, but significantly lower compared to the ketam [file enu-eN-NWR-0343-20-s10.tif]

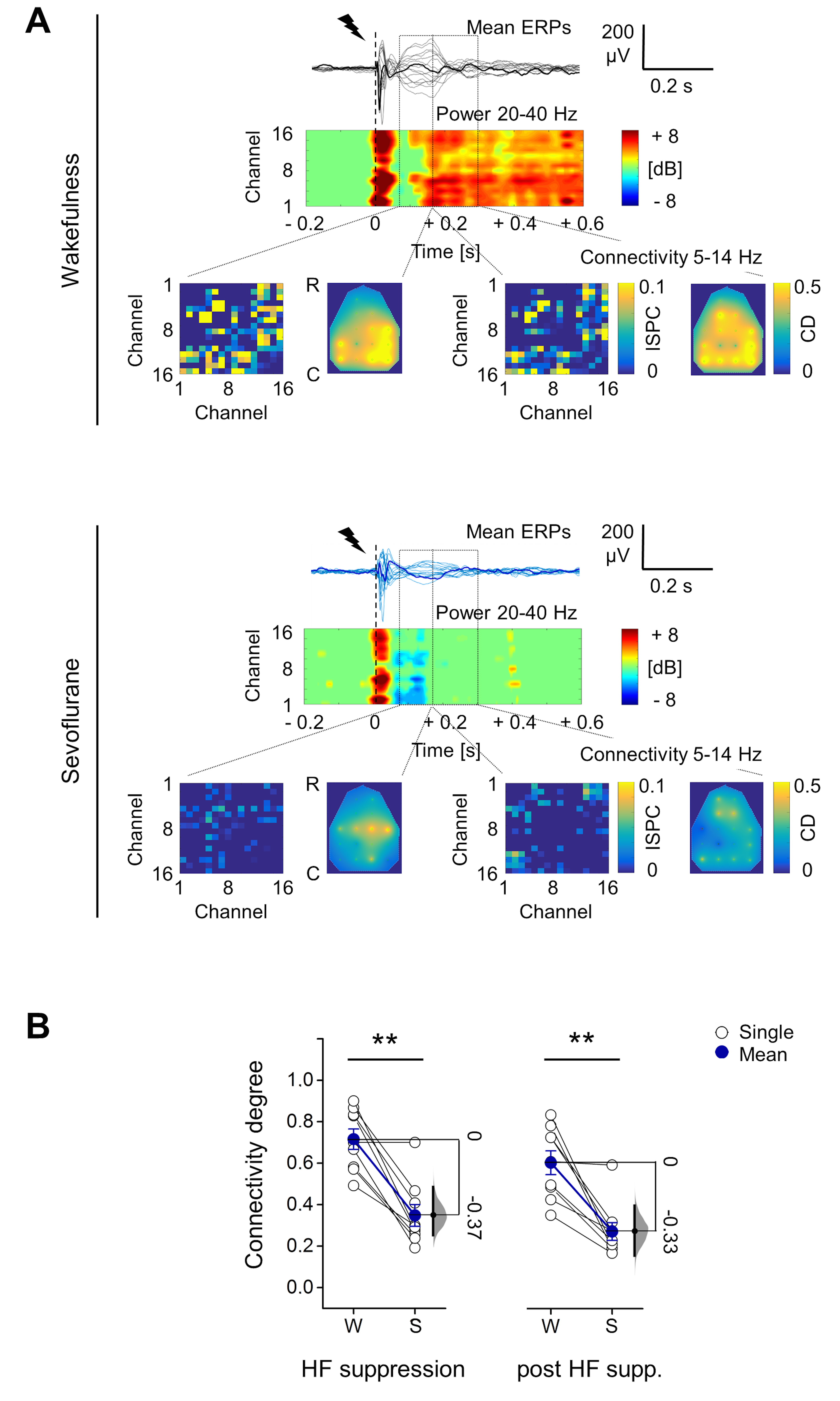

Supplement: Extended Data Figure 5-1 — Functional connectivity after cortical perturbation during sevoflurane anesthesia. A, Example of epidural EEG activity in response to electrical stimulation (1 ms, 50 μA; dashed lines) from the same rat during wakefulness and during sevoflurane anesthesia (same rat of Fig. 5 to allow comparisons). The electrophysiological traces represent the superimposition of ensemble averages of ERPs from all electrodes. One channel is highlighted in bold for better illustrating difference in complexity. Below each butterfly plot, the spectrogram of mean relative HF power (20–40 Hz) is shown for each channel. The bottom part of each inner panel shows the increments in cortical connectivity compared to baseline for each condition in two time windows: during HF suppression, 0.08–0.18 s (left) and post-HF suppression, 0.18–0.3 s (right; rectangles indicate the two time windows). For each window, the connectivity matrix based on ISPC (5–14 Hz) is reported on the left and the topographical distribution (rostro-caudal orientation, R-C) of the CD for each channel is interpolated and shown on the right. B, Mean CD across channels in the HF suppression period (left) and post-HF suppression (right) during wakefulness and sevoflurane anesthesia (n = 9 rats). During wakefulness CD was significantly higher than what observed during sevoflurane anesthesia in both time windows (HF suppression period, wakefulness: 0.71 ± 0.05, sevoflurane: 0.35 ± 0.05, mean difference = –0.37 [95% CI: –0.46, –0.23]; Wilcoxon S-R test, p = 0.008; post-HF suppression, wakefulness: 0.60 ± 0.06, sevoflurane: 0.27 ± 0.04; mean difference = –0.33 [95% CI: –0.45, –0.21]; Wilcoxon S-R test, p = 0.004). The floating axis on the right of each group comparison shows the mean difference between conditions. The effect size is reported as bootstrap resampling distribution of mean difference, with 95% CI represented by the bold black vertical line. Download Figure 5-1, TIF file. [file enu-eN-NWR-0343-20-s11.tif]

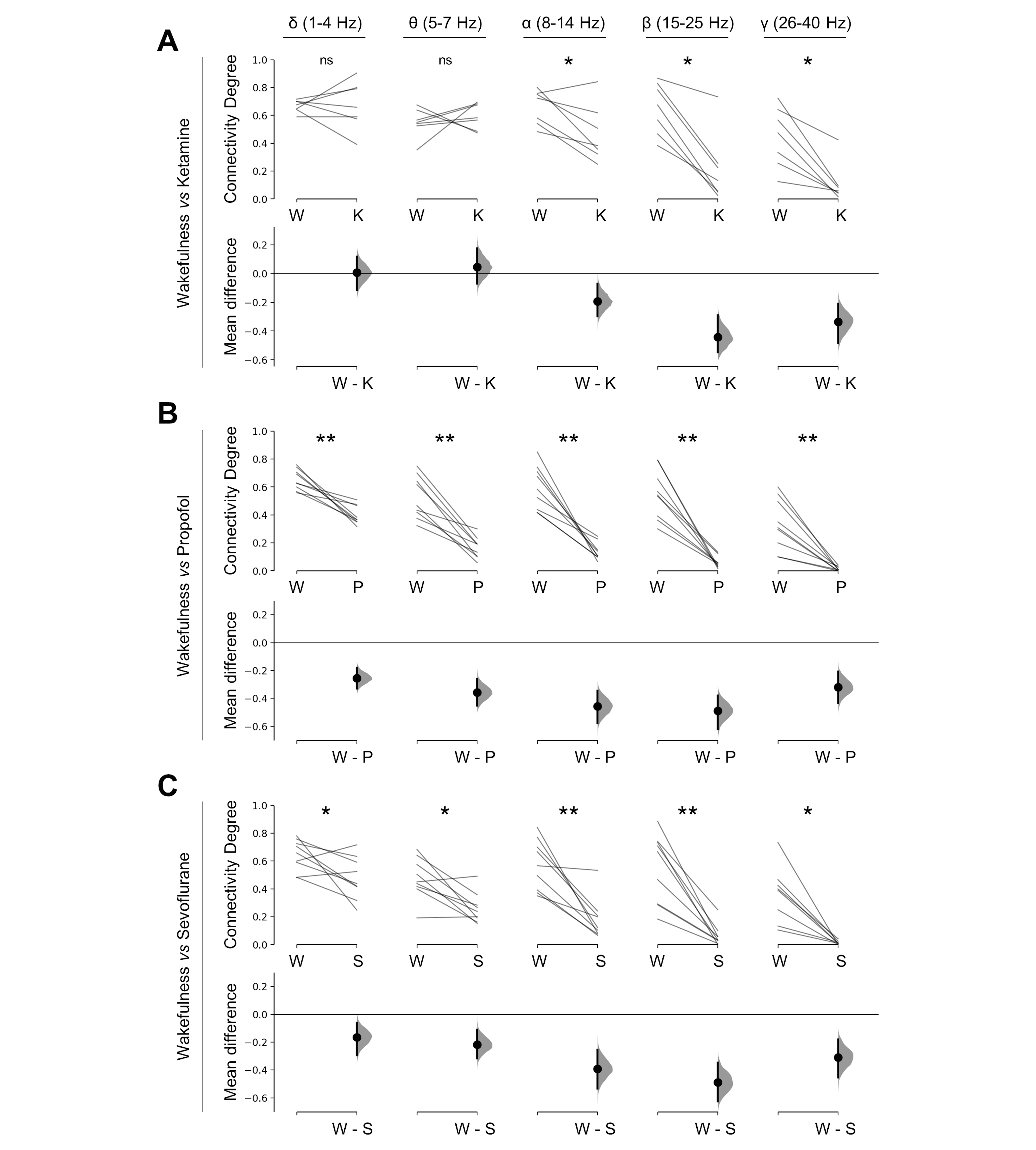

Supplement: Extended Data Figure 5-2 — Functional connectivity across conditions for each frequency band, A–C, Mean CD across channels following the period of HF suppression (0.18–0.3 s), from rats during wakefulness and ketamine anesthesia (A, n = 7), wakefulness and propofol anesthesia (B, n = 9), and wakefulness and sevoflurane anesthesia (C, n = 9). All comparisons are reported for each frequency band of interest (columns; from 1 to 40 Hz). During wakefulness, CD was significantly higher than what was observed during all anesthesia conditions, for each frequency band, with the exceptions of δ (1–4 Hz) and θ (5–7 Hz) bands with ketamine, for which no significant difference was identified. The mean difference between conditions is reported for each frequency band, below each group comparison. The effect size is reported as bootstrap resampling distribution of mean difference, with 95% CI represented by the bold black vertical line. Wilcoxon S-R test was adopted to assess statistical significance, which is reported in the figure as follows: *p < 0.05, **p < 0.01, ***p < 0.001, p ≥ 0.05 ns (not significant). Download Figure 5-2, TIF file. [file enu-eN-NWR-0343-20-s12.tif]

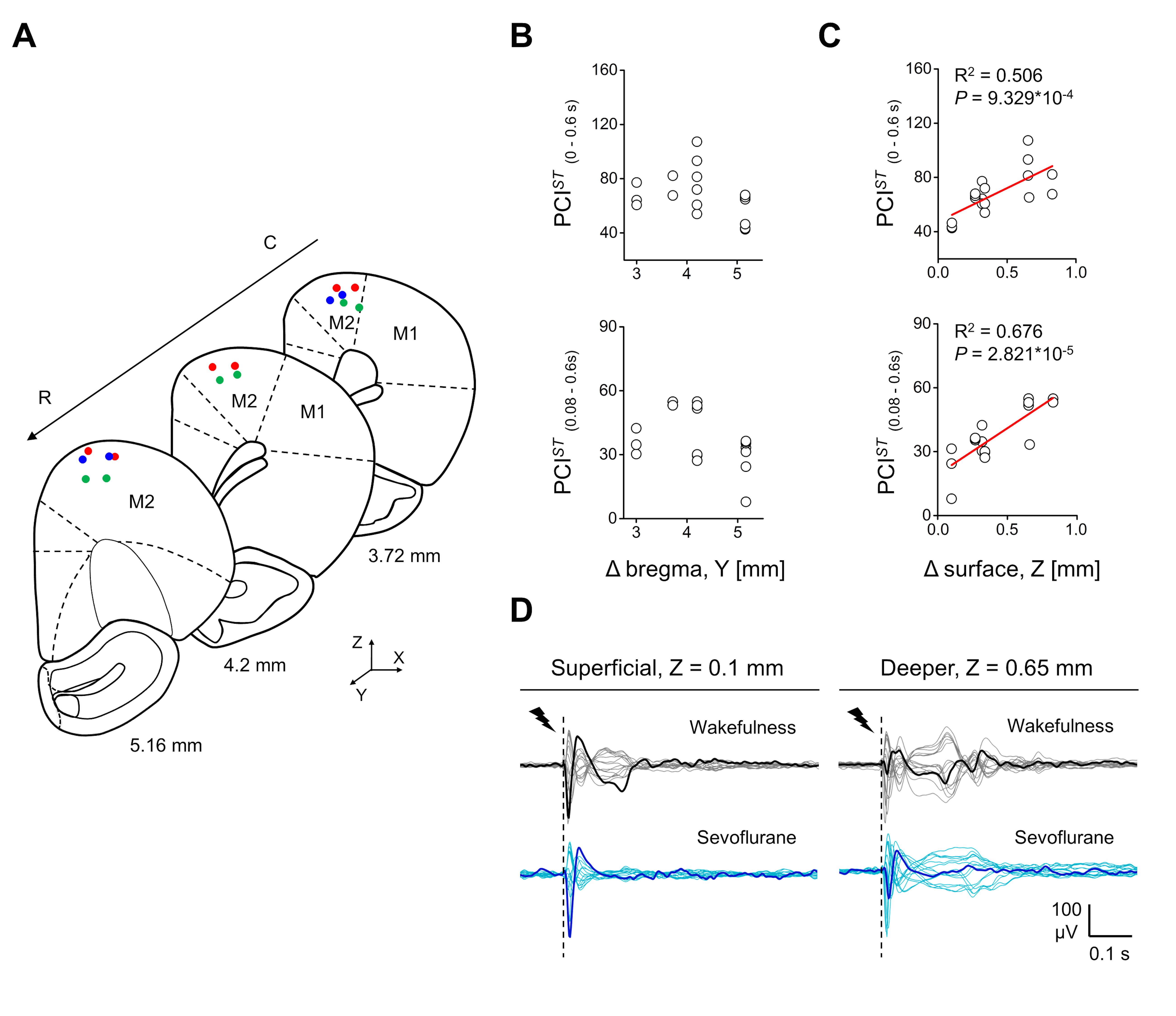

Supplement: Extended Data Figure 6-1 — Correlation of PCIST with the stimulus location in M2 during wakefulness. A, The same representation of coronal sections of rat brain with the actual position of chronically implanted bipolar electrodes in right M2 has already been shown in Extended Data Figure 1-3 and is reported here for clarity. Dots represent the two poles of the bipolar electrodes chronically implanted in eight rats (for each section, bipolar electrodes from different rats are color coded). Stimulating electrodes covered a cortical area from ∼5 to ∼3 mm with respect to bregma in rostro-caudal direction (R-C, y-axis) and a depth range from ∼0.1 to ∼0.8 mm calculated from cortical surface (z-axis), mainly corresponding to Layer II/III. In one rat the electrode was found to be placed in Layer I, while in another animal it was positioned at the edge between Layers III and V. B, C, PCIST from time range 0–0.6 s (up) and from time window 0.08–0.6 s (bottom) are plotted for each rat (n = 7) and recording session during wakefulness (one to three recordings for each rat) against the position of the stimulating electrode along the y-axis (B) and along the z-axis (C). Possible correlations with the position of stimulating electrode have been tested and no significant relation was detected between PCIST and the position of stimulating electrodes along the y-axes (B; up, PCIST(0–0.6 s), linear fit, p = 0.155, R2 = 0.122; bottom, PCIST(0.08–0.6 s), linear fit, p = 0.129, R2 = 0.138). Otherwise, a highly significant and strong positive correlation between PCIST (in both time windows) and the depth of the stimulation site have been found (C; up, PCIST(0–0.6 s), linear fit, p = 9.329 × 10^−4^, R2 = 0.506; bottom, PCIST(0.08–0.6 s), linear fit, p = 2.821 × 10^−5^, R2 = 0.676). The coefficient of determination R2 and the p value are reported. D, Example of evoked activity from one rat with stimulating electrode implanted close to cortical surface (left) and from other animals with stimulating electrode placed deeper [file enu-eN-NWR-0343-20-s13.tif]

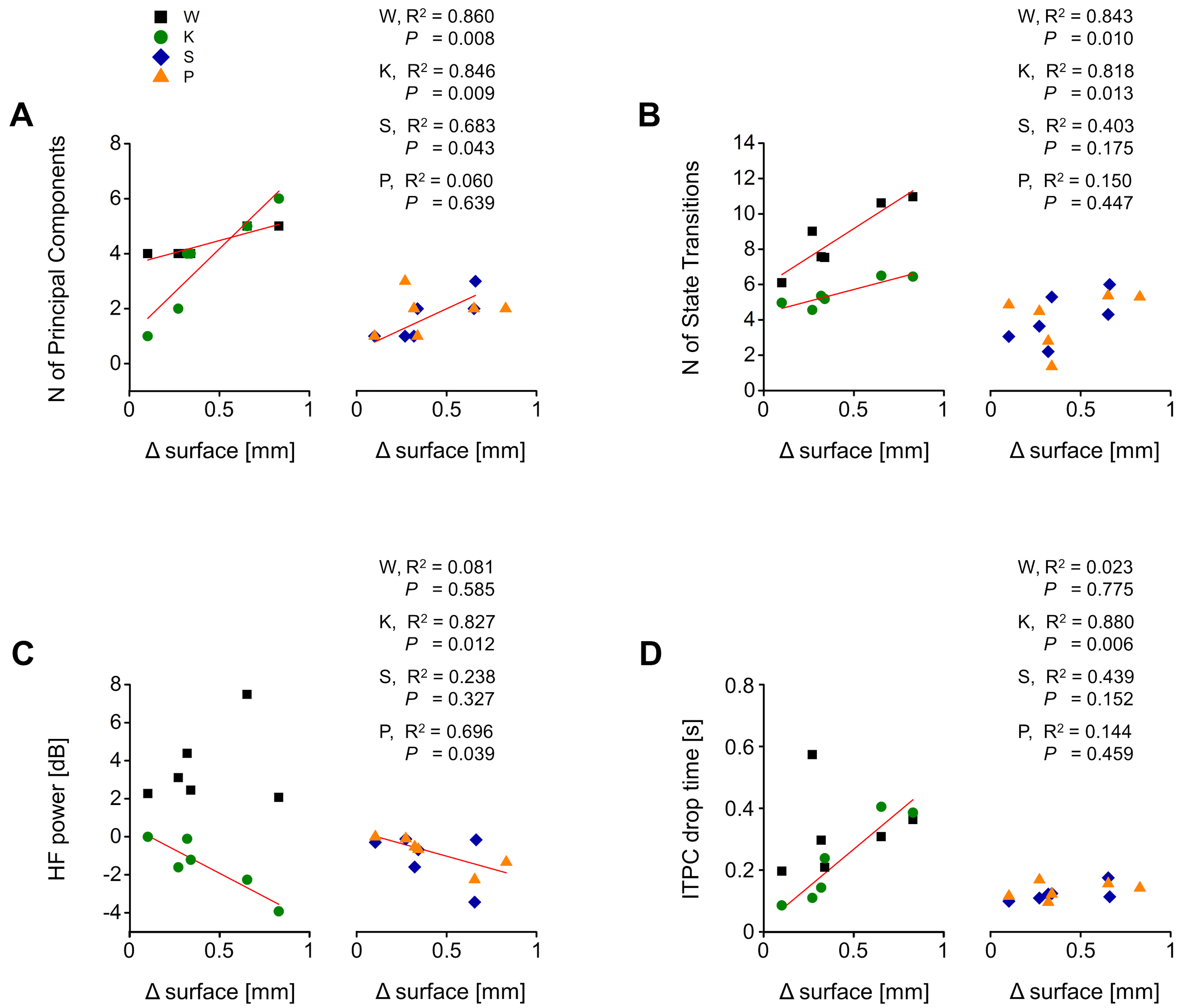

Supplement: Extended Data Figure 6-2 — Dependency of ERP features on stimulation depth. We tried to clarify the dependency of PCISTon stimulation depth by analyzing the number of principal components (NPC) and NST, since PCIST is equivalent to the product of NPC and NST, as well as HF power and ITPC drop time in relation to stimulus position in the same six rats of Figure 6, during wakefulness (W), propofol (P), sevoflurane (S), and ketamine (K) conditions. As expected, both the NPC (A) and NST (B) increased with the stimulation depth in both wakefulness and ketamine conditions (with different slopes). The NPC increased also with sevoflurane as a function of stimulus depth, but this increment did not translate into higher PCIST (see Fig. 6). C, The averaged HF power (in range 20–40 Hz, 0.08–0.18 s) became more negative by increasing the stimulation depth in both ketamine and propofol condition. D, The ITPC drop time occurred later with deeper stimulations only with ketamine. Measurements from 6 rats and for all conditions are plotted against the corresponding distances of the stimulating electrode tips from the cortical surface and linearly fitted (fitting function, coefficient of determination R2 and p value are reported if p < 0.05). Download Figure 6-2, TIF file. [file enu-eN-NWR-0343-20-s14.tif]

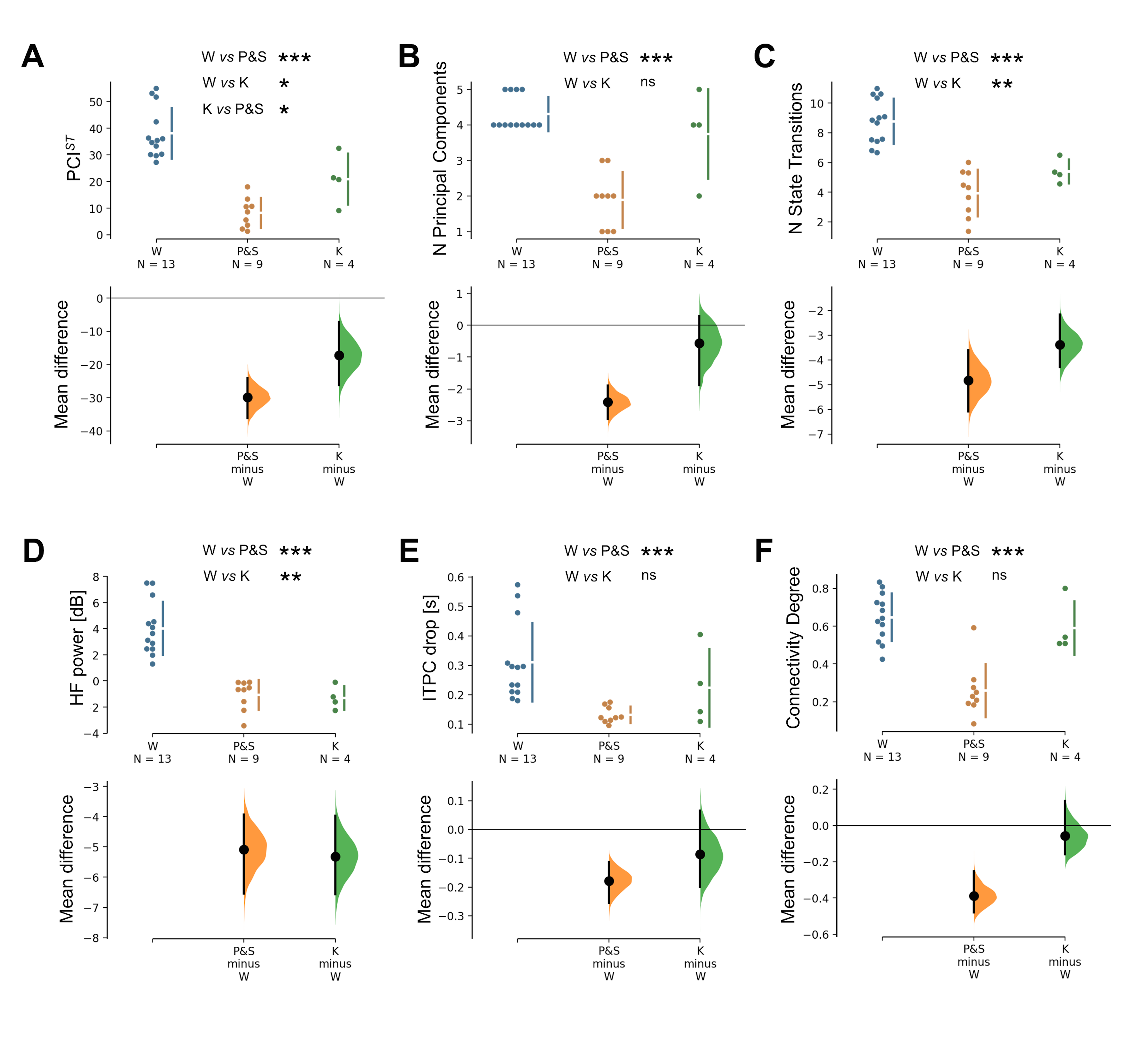

Supplement: Extended Data Figure 6-3 — Comparison of ERPs between wakefulness and anesthesia for rats with colocalized stimulating electrode in Layer II/III. A–F, Only rats with confirmed colocalization of stimulating electrodes in Layer II/III were considered for this analysis (n = 5 rats). Because of the reduced total amount of animals, to increase the statistical power, data from different recording sessions from the same rats were grouped together in wakefulness condition (W), generating a sampling size of 13 observations (four rats with data from three recordings each, plus one rat with data from one recording). With the same purpose, data from general anesthesia produced by GABAergic drugs (sevoflurane and propofol, P&S) were grouped together, generating a sample size of nine observations (four rats with data from both anesthetic conditions, plus one rat with data only from sevoflurane condition). Data from four rats, each with one observation, belonged to ketamine condition (K). Several features of the evoked response were compared across conditions at the level of observations. A, PCIST (in time range 0.08–0.6 s) decreased from wakefulness to all other conditions; however, with ketamine, it was higher than in the propofol and sevoflurane group. B, This was explained by a reduced number of principal components from W to P&S, while no significant difference was detected between W and K. C, Conversely, the NST was reduced from wakefulness to both P&S and K conditions. D, In P&S and K conditions, the mean HF power (in range 20–40 Hz, 0.08–0.18 s) was always below 0 dB, indicating a suppression period. On the contrary, it was always above 0 dB, thus significantly higher during wakefulness. E, The averaged ITPC drop time (in range 8–40 Hz) during wakefulness occurred significantly later than in propofol and sevoflurane anesthesia, while no significant difference was found in comparison to ketamine condition. F, Similarly, the mean CD (in range 5–14 Hz, 0.18–0.3 s) dropped from W to P&S, while no signif [file enu-eN-NWR-0343-20-s15.tif]
